# Characterization of the Resistance to Powdery Mildew and Leaf Rust Carried by the Bread Wheat Cultivar Victo

**DOI:** 10.3390/ijms22063109

**Published:** 2021-03-18

**Authors:** Francesca Desiderio, Salim Bourras, Elisabetta Mazzucotelli, Diego Rubiales, Beat Keller, Luigi Cattivelli, Giampiero Valè

**Affiliations:** 1 CREA Research Centre for Genomics and Bioinformatics, 29017 Fiorenzuola d’Arda, Italy; elisabetta.mazzucotelli@crea.gov.it (E.M.); luigi.cattivelli@crea.gov.it (L.C.); 2 Department of Plant and Microbial Biology, University of Zurich, 8008 Zurich, Switzerland; salim.bourras@slu.se (S.B.); bkeller@botinst.uzh.ch (B.K.); 3 Department of Forest Mycology and Plant Pathology, Swedish University of Agricultural Sciences, 75651 Uppsala, Sweden; 4 Institute for Sustainable Agriculture, CSIC, 14004 Córdoba, Spain; diego.rubiales@ias.csic.es; 5 DiSIT—Dipartimento di Scienze e Innovazione Tecnologica, Università del Piemonte Orientale, 13100 Vercelli, Italy; giampiero.vale@uniupo.it

**Keywords:** *Triticum aestivum*, powdery mildew, leaf rust, molecular marker, QTL

## Abstract

Leaf rust and powdery mildew are two important foliar diseases in wheat. A recombinant inbred line (RIL) population, obtained by crossing two bread wheat cultivars (‘Victo’ and ‘Spada’), was evaluated for resistance to the two pathogens at seedling stage. Upon developing a genetic map of 8726 SNP loci, linkage analysis identified three resistance Quantitative Trait Loci (QTLs), with ‘Victo’ contributing the resistant alleles to all loci. One major QTL (Q*Pm.*gb-7A) was detected in response to *Blumeria graminis* on chromosome 7A, which explained 90% of phenotypic variation (PV). The co-positional relationship with known powdery mildew (*Pm*) resistance loci suggested that a new source of resistance was identified in *T. aestivum*. Two QTLs were detected in response to *Puccinia triticina*: a major gene on chromosome 5D (Q*Lr*.gb-5D), explaining a total PV of about 59%, and a minor QTL on chromosome 2B (Q*Lr*.gb-2B). A positional relationship was observed between the Q*Lr*.gb-5D with the known *Lr*1 gene, but polymorphisms were found between the cloned *Lr*1 and the corresponding ‘Victo’ allele, suggesting that Q*Lr*.gb-5D could represent a new functional *Lr*1 allele. Lastly, upon anchoring the QTL on the *T. aestivum* reference genome, candidate genes were hypothesized on the basis of gene annotation and in silico gene expression analysis.

## 1. Introduction

Powdery mildew and leaf rust, caused by *Blumeria graminis* f. sp. *tritici* and *Puccinia triticina*, respectively, are important and widespread wheat (*Triticum. aestivum* L.) fungal diseases. They can cause up to 40% yield loss in susceptible cultivars by decreasing kernel number and kernel weight [1,2]. Chemical control is extensively used to maintain high yields when susceptible cultivars are grown. Instead, the deployment of genetically resistant cultivars obtained by breeding is considered as a cost-effective and environmentally safe disease control strategy.

Race-specific and race non-specific types of genetic resistance towards pathogens are generally found in plants. The former is qualitative, usually mediated by a single major gene (R), which is active upon recognition of pathogen’s effectors in a gene-for-gene relationship. Race-specific resistance is highly valuable in breeding because it confers near immunity towards specific pathogen races [3]. However, due to the rapid evolution of pathogen populations, major R genes are known to break down readily resulting in a limited lifespan of resistance [4]. Major R genes commonly encode for immune receptors from the Nucleotide-binding Leucine-Rich Repeats (NLR) type, with a few prominent exceptions including *Stb*6, a wheat resistance gene against *Zymoseptoria tritici* identified as a wall-associated receptor kinase-like protein [5]. Gene combinations, strategic gene deployment, and multiline cultivars are suggested strategies to expand the lifespan of race-specific resistance genes in the field [6]. Race-non-specific resistance is generally quantitative, controlled by several genes with major and minor effects, and typically active at the adult stage. While breeding of wheat cultivars with partial resistance is considered a sustainable strategy to control diseases [7], wheat breeding programs typically combine both major R genes and partial resistance in an attempt to achieve high levels of durable resistance.

Approaches commonly used to discover loci associated with complex traits in plants are linkage and association mapping [8]. Although association mapping is a relatively recent approach and found effective to locate genomic loci associated with complex traits in plants [9], linkage mapping has been extensively and successfully used to map and clone genes associated with various traits in plants.

To date, 54 powdery mildew (*Pm*) and 72 leaf rust (*Lr*) resistance genes have been identified and mapped in wheat and its relatives. Most of them are race specific, and only 4 *Pm* and 12 *Lr* confer broader resistance to their respective diseases [10,11]. Additionally, four identified *Pm* genes (*Pm*38, *Pm*39, *Pm*46, and *Pm*48) confer a pleiotropic effect on other disease responses, including leaf rust, stripe rust, and stem rust (*Yr*18/*Lr*34/*Pm*38/*Sr*57, *Yr*29/*Lr*46/*Pm*39/*Sr*58, *Yr*30/*Lr*27/*Pm*48/*Sr*2, and *Yr*46/*Lr*67/*Pm*46/*Sr*55) [12,13,14,15,16]. Thus, such genes are highly valuable for breeding multiple disease resistance in wheat.

Of these mapped genes, only sixteen conferring resistance to powdery mildew (*Pm*3 allelic series, *Pm*8, *Pm*17, *Pm*41, *Pm*24, *Pm*21, *Pm*2, *Pm*60, *Pm*1a, and *Pm*5e) and leaf rust (*Lr*1, *Lr*10, *Lr*21, *Lr*22a, *Lr*34, and *Lr*67) have been cloned so far in wheat [17,18,19,20,21,22,23,24,25,26,27,28,29,30,31,32,33,34], and the majority were identified using the linkage mapping approach.

Most of these gene are race-specific and encode typical NLR-type proteins including *Lr*1, *Lr*21, *Lr*10, *Pm*3b, *Pm*2, *Pm*60, and *Pm*21 in wheat. Others, such as *Lr*34 and *Lr*67, are race-non-specific and encode an ATP-binding cassette (ABC) transporter or a hexose transporter, respectively.

The current study aims to expand the current knowledge on bread wheat resistance to powdery mildew and leaf rust through the identification of novel resistance loci by means of artificial inoculation at the seedling stage of a Recombinant Inbred Line (RIL) population derived from a cross between the cultivars (*cvs*) ‘Victo’ (resistant) and ‘Spada’ (susceptible). The objectives of this work were (i) to develop a high-density genetic map, (ii) to conduct a QTL mapping of the genetic basis of powdery mildew and leaf rust resistances, and (iii) to propose a list of candidate genes that could be involved in the resistance.

## 2. Results

### 2.1. Phenotypic Evaluations of Leaf Rust and Powdery Mildew Resistance

Evaluation of the leaf rust and powdery mildew responses provided a clear discrimination between the two parents. For both isolates of *Puccinia triticina* (*Pt*_Jerez05 and *Pt*_VMC03) ‘Victo’ showed a high level of resistance (Infection Type, IT,0), while ‘Spada’ was clearly susceptible (IT9). Regarding the powdery mildew response, three different isolates (*Bgt*_96224, *Bgt*_94202, and *Bgt*_JIW2) were tested on the parental lines. The bread wheat cultivar ‘Victo’ was resistant toward *Bgt*_JIW2 and *Bgt*_96224, showing a resistance level ranging from IT3 to IT4, while ‘Spada’ was susceptible with values higher than IT7 (Table 1). The 128 RILs were subjected to artificial inoculation with leaf rust (isolate *Pt_*VMC03) and powdery mildew (isolate *Bgt*_JIW2) isolates: IT values for each pathogen and disease severity only for *Pt*_VMC03 were recorded.

**Figure 1:**
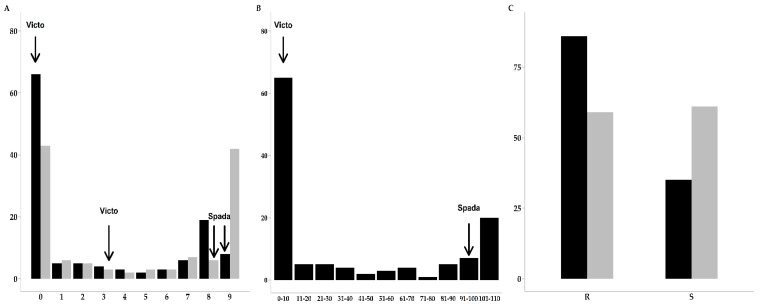
Frequency distribution of phenotypic reaction to leaf rust (black) and powdery mildew (grey) isolates expressed as infection type (**A**) or relative disease severity (**B**). The frequency distribution using the major two classes of infection type were reported (**C**) as resistant (R) and susceptible (S). Reaction scores of the parents are indicated by arrows. The *y*-axis shows the number of lines.

The frequency distribution of phenotypic reactions for RILs is shown in Figure 1. Transgressive segregation was observed for the two disease severity assessments. Lines more resistant than ‘Victo’ (IT higher than 8) were identified toward powdery mildew, and lines more susceptible than ‘Spada’ were retrieved in response to *Blumeria graminis* and *Puccinia triticina* using IT and Relative Disease Severity (RDS) values, respectively. A significant correlation was observed only between RDS and IT values detected for leaf rust response (r = 0.99; *p* < 0.0001).

Disease symptoms, as indicated by the IT values, were used to classify the RILs into two classes for both diseases. For leaf rust reaction, the first class contained resistant RILs showing IT values between 0 and 6, while the susceptible class included lines with IT values from 7 to 9. The classes for powdery mildew instead are composed by resistant lines that showed an IT value ranging from 0 to 5, while the susceptible lines had IT values from 6 to 9. Analysis of the obtained frequency distribution of the phenotypic reactions (Figure 1) indicated a significantly higher number of RILs in the resistant class than in the susceptible one for leaf rust, while a similar number of lines fell into the two major phenotypic classes when considering the powdery mildew data. The observed segregation ratio (*p* < 0.05; Table 2) of susceptible and resistant lines revealed that the resistance associated to powdery mildew could be under control of a major genetic factor, while more loci could be involved in the leaf rust response.

Variation for the phenotypic data for each disease was assessed by ANOVA, considering the effects of genotypes and replications. Highly significant genotypic effect was detected for both diseases and confirmed by the calculated broad sense heritability (Table S1). The estimates of broad sense heritability for powdery mildew and leaf rust resistances were 0.92 and 0.97, respectively, indicating that most of the phenotypic variance was due to genetic effects.

### 2.2. Construction of the Victo x Spada Genetic Linkage Map

Out of 67,799 high-quality SNP markers, 9828 SNPs were polymorphic between the parental lines. After elimination of unlinked loci, the genotype data relating to 8726 informative SNPs were assembled into 30 linkage groups corresponding to the 21 bread wheat chromosomes (Table 3).

More than one linkage group was obtained for chromosomes 2D, 3A, 4A, 4D, 6D, 7A, 7B, and 7D. The overall length of the map was 3291.5 cM with individual chromosome genetic length ranging from 31.8 cM (chromosome 4D) to 323.2 cM (chromosome 5A) and an average of 156.74 cM. The total number of mapped loci per chromosome ranged from 16 (chromosome 4D) to 801 (chromosome 1B) with an average of 415.52 loci per chromosome. Details about the genetic distances between markers are provided in Table S2. The genome-wide mean inter-locus separation was 0.38 cM, varying from 0.23 cM (chromosomes 1A, 1B, 6D) to 1.99 cM (chromosome 4D) (Table 3). Considering the homeologous groups, group 1 showed both the highest number of mapped loci (1651) and the highest marker density (mean of 0.27 cM/marker), while group 5 had the longest map length (660.4 cM) (Table 3). Differences were also observed between the three subgenomes (A, B, and D). The subgenomes A and B showed similar values in map length and marker density, while subgenome D was less polymorphic compared with the other two, as already described previously in bread wheat linkage maps developed using the 90k array [35,36,37,38].

### 2.3. Identification of QPm.gb-7A, a Powdery Mildew Resistance QTL on Chromosome 7AL

The analysis of powdery mildew resistance allowed the identification of one associated region on the long arm of chromosome 7A (named as Q*Pm.*gb-7A; Table 4).

This QTL, with a confidence interval of 1.4 cM, explained 90% of phenotypic variation, and the resistant parent ‘Victo’ contributed the allele with the positive effect. Furthermore, on the basis of the physical position of associated markers on the Chinese Spring (CS) reference genome, we defined that the QTL region extends for 6.35 Mbp, from 724,084,703 to 730,433,215 bp (Table 5).

To date, a number of powdery mildew resistance genes have been identified on chromosome 7AL (Table S3). To compare the Q*Pm.*gb-7A region identified in this work, with known regions controlling resistance to powdery mildew, a physical map was developed using all markers associated to powdery mildew resistance loci retrieved from the genetic map regions previously identified. The molecular markers were then ordered on the basis of their physical position through BLAST search on the CS reference genome.

**Figure 2:**
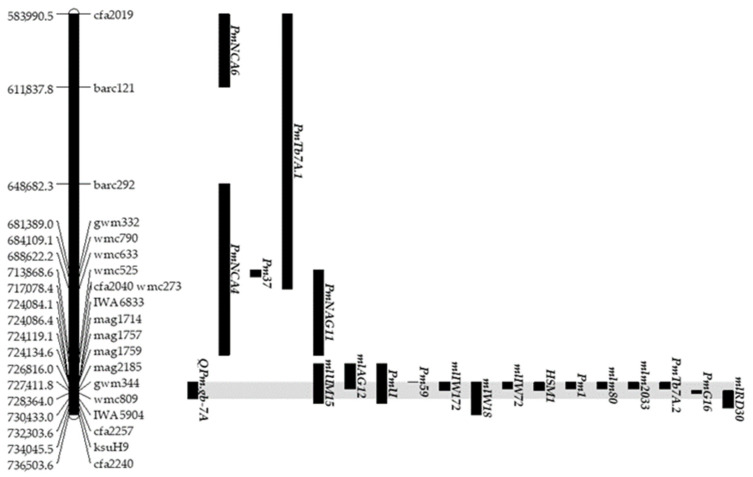
Physical map of Q*Pm.*gb-7A and other regions harboring significant effects for powdery mildew resistance. The physical position, expressed in kilobases, and the flanking molecular markers of each QTL are reported.

Q*Pm.*gb-7A spanned a region of approximately 6.35 Mbp, between marker IWA6833 at position 724.0 Mbp and marker IWA5904 at position 730.4 Mbp (Figure 2).

In the surrounding of Q*Pm.*gb-7A, nineteen powdery mildew resistant loci have been reported.

i. Five loci were located upstream Q*Pm.*gb-7A: *Pm*NCA6 (from 583.9 to 611.8 Mbp), *Pm*Tb7A.1 (from 583.9 to 688.6 Mbp), *Pm*37 (from 681.3 to 684.1 Mbp), *Pm*NAG11 (from 681.3 to 713.8 Mbp), and *Pm*NCA4 (from 590.2 to 713.8 Mbp) [39,40,41,42].
ii. Two loci have an overlapping region with Q*Pm.*gb-7A from 2.73 to 3.01 Mbp: *ml*AG12 (from 717.0 to 726.8 Mbp) and *ml*RD30 (from 727.4 to 734.0 Mbp) [43,44].
iii. Q*Pm.*gb-7A was included completely in the physical regions, from 12.37 Mbp to 15.22 Mbp, identified by *Pm*U (from 717.0 to 732.3 Mbp), *ml*W18 (from 724.1 to 736.5 Mbp), and *ml*UM15 (from 717.0 to 732.3 Mbp) [45,46,47].
iv. Nine additional resistance loci showed a co-positional relationship of physical region with Q*Pm.*gb-7A: *Pm*59 (from 724.0 to 724.1Mbp), *ml*IW172 (from 724.1 to 727.4 Mbp), *Pm*1, *ml*IW72, *mlm*80, *Pm*Tb7A.2 and *mlm*2033.(from 724.1 to 726.8), *HSM*1 (from 724.1 to 727.4 Mbp), and *Pm*G16 (from 727.4 to 728.3 Mbp) [40,48,49,50,51,52,53,54,55].

Overall, these data indicate that the long arm of chromosome 7A is particularly rich in powdery mildew resistance genes that come from different *Triticum* species. Only three of them, *ml*RD30 [44], *Pm*59 [48], and *Pm*1 [49,50], were detected in hexaploid wheat, while the other resistance loci are derived from tetraploids *T. dicoccoides* (*ml*IW72, *ml*IW172, *ml*W18, *HSM*1, and *Pm*G16), *A. neglecta* (*ml*UM15), *T. timopheevi* (*ml*AG12), and from diploids *T. boeticum* (*Pm*Tb7A.2 and *mlm*80), *T. urartu* (*Pm*U), and *T. monococcum* (*mlm*2033) [40,43,45,46,47,51,52,53,54,55].

**Figure 3:**
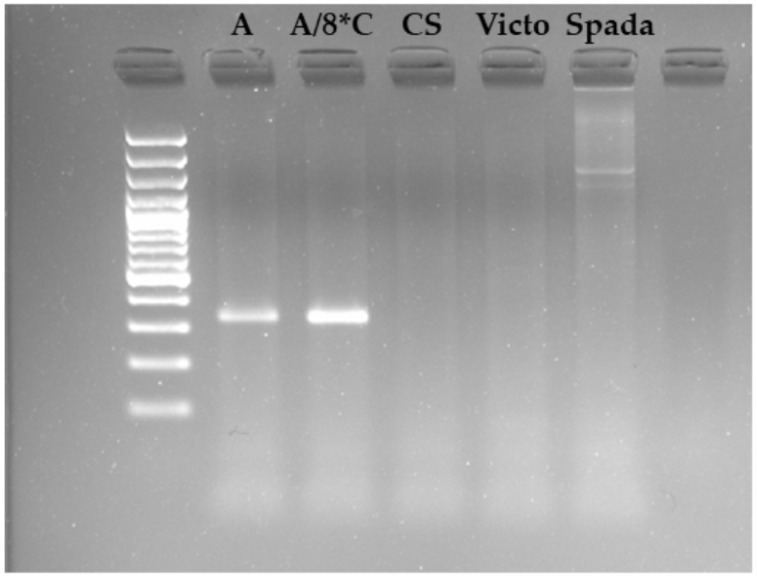
Marker *Pm*1aSTS1 assay showing specificity to *Pm*1a-containing *T. aestivum* lines. Victo, Spada, and Chinese Spring (CS) do not have the *Pm*1a gene, while Axminster (A) and Axminster/8*Chancellord (A/8*C) do have it.

The *Pm*1aSTS1 functional assay, recently developed to detect the *T. aestivum* lines carrying the *Pm*1a gene [33], was used to verify the presence of the gene in the ‘Victo’ genetic background. As shown in Figure 3 no amplification was obtained from ‘Victo’ genomic DNA; therefore, we can conclude that the powdery mildew-resistant gene identified in ‘Victo’ is not conferred by *Pm*1a.

Allelism analyses would be required to verify whether *Pm*59, *ml*RD30, and Q*Pm.*gb-7A represent different resistance genes or alleles; if this difference will be proved, Q*Pm.*gb-7A would represent a new source of powdery mildew resistance in *T. aestivum*.

Currently, only one powdery mildew resistance QTL, Q*Pm.*umb-7AL, was mapped to the long arm of chromosome 7A [56]. Information available for Q*Pm.*umb-7AL did not allow the identification of a precise physical location. The Q*Pm.*umb-7AL proximal marker (gwm428) did not allow identification of a BLAST match on chromosome 7A. The Q*Pm.*umb-7AL distal marker (cfa2040) was physically mapped on chromosome 7A at 717,078,361 bp. On the basis of these results, the Q*Pm.*umb-7AL was positioned at about 7 Mbp from Q*Pm.*gb-7A, suggesting that Q*Pm.*gb-7A was different from the region previously identified by Lillemo et al. [56].

### 2.4. Identification of QLr.gb-5D, a with Leaf Rust Resistance QTL on Chromosome 5DL

Two QTLs with major effects towards both phenotypic trait (IT and RDS) recorded for leaf rust were mapped to chromosome 5D. Since the map positions of the two QTLs were coincident, even considering that the possibility of linked genes cannot be ruled out, this locus with major effects on leaf rust resistance was designed as a single QTL named Q*Lr*.gb-5D. This region, whose effective allele was contributed by the resistant parent ‘Victo’, explained approximately 59% of phenotypic variance, considering both IT and RDS, while the confidence interval of 2.15 cM delineates a physical region of 2.498 Mbp (from 560,228,250 to 562,726,656 bp; Table 4 and Table 5).

Two additional significant regions towards IT and RDS of isolate VMC03 were localized on chromosome 2BL (Table 4). Additionally, in this case, the map position of the two QTLs was coincident and therefore considered as a single QTL (Q*Lr*.gb-2B). Q*Lr*.gb-2B showed significant values of variance explained (about 16%), and here as well ‘Victo’ contributed the resistance allele. Finally, 6% of the variance was explained by the interactions between the two detected QTLs (Table 4).

Currently, eight *Lr* genes (*Lr*57, *Lr*76, *Lr*70, *Lr*78, *Lr*Ac, *Lr*1, *Lr*Syn137, and *Lr*LB88) have been mapped on chromosome 5D. Of these, *Lr*1 [17], *Lr*Syn137 [57], and *Lr*LB88 [58] were located on the long arm of chromosome 5D.

**Figure 4:**
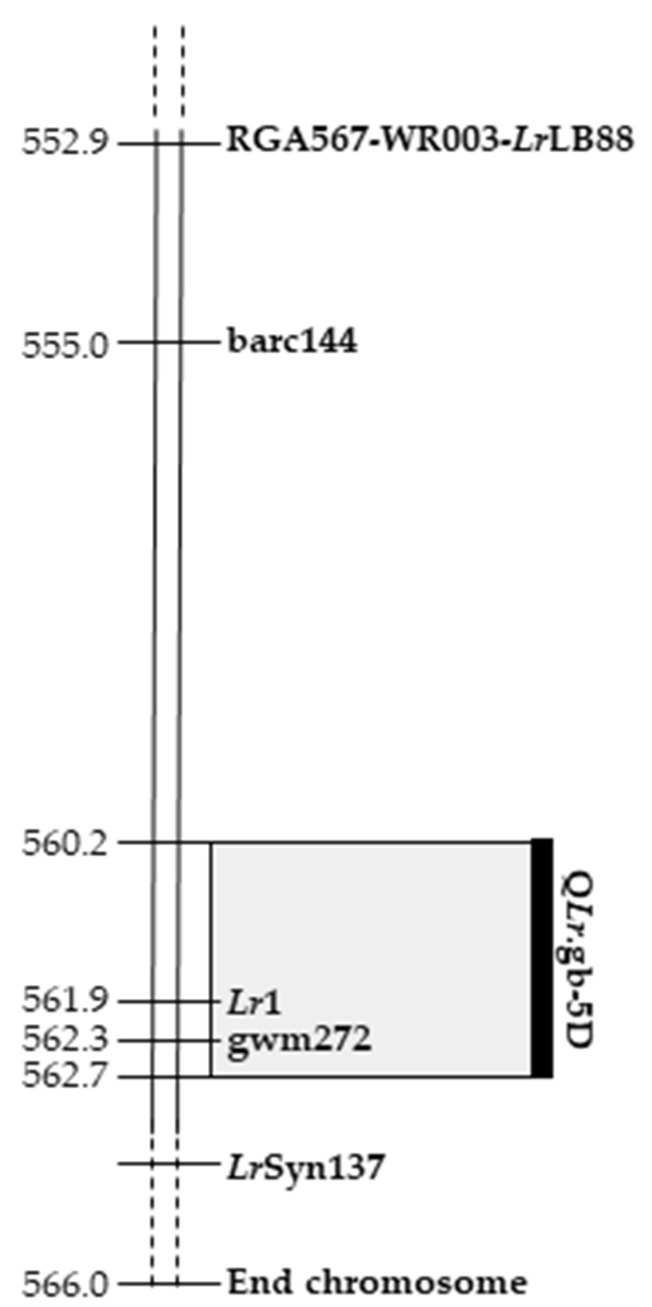
Relationships of Q*Lr*.gb-5D with leaf rust resistance genes (*Lr*1, *Lr*LB88 and *Lr*Syn137) previously mapped on chromosome 5DL. The physical position of molecular markers retrieved from the CS reference genome are reported.

Relationships among Q*Lr*.gb-5D and the other leaf rust genes previously mapped in the surrounding region were analyzed to highlight possible positional relationships (Figure 4).

No common markers were available when the map position of previously mapped *Lr* genes and the one of Q*Lr*.gb-5D were compared. Therefore, analogously to the procedure used for Q*Pm.*gb-7A, the physical positions of markers co-segregating/more associated to the *Lr* genes were retrieved through BLAST search on the CS reference genome.

*Lr*LB88 markers were localized proximally to Q*Lr*.gb-5D, with the gene and the co-segregating functional marker WR003 (552.9 Mbp) being positioned at approximately 7.3 Mbp from Q*Lr*.gb-5D.

*Lr*Syn137 was mapped in distal position to the *Lr*LB88 functional marker WR003 (552.9 Mbp) as well with respect to gwm272 (562,333,785 bp) [57]. Information available for *Lr*Syn137 does not allow the identification of a precise physical location for this locus but suggests that its position is distal with respect to Q*Lr*.gb-5D.

Overall, these comparisons are supporting a Q*Lr*.gb-5D physical position different from *Lr*Syn137 and *Lr*LB88.

For the analysis of co-positional relationships with *Lr*1, the cloned sequence [17] was used to identify its physical region on the CS reference genome. This analysis revealed that the *Lr*1 region in CS spans the sequence between position 561,894,974 to 561,900,201 bp on chromosome 5D. Considering that the Q*Lr*.gb-5D physical interval extends from position 560,228,250 to 562,726,656 bp, the observed relationship raised the possibility that Q*Lr*.gb-5D represents the ‘Victo’ allele of the *Lr*1 gene (Figure 4).

To analyze homology relationships between the *Lr*1 alleles, the *Lr*1 gene was amplified (primer pairs in Table S4) and sequenced in the parental lines ‘Victo’ and ‘Spada’. The putative *Lr*1 ‘Victo’ allele was then used to perform BLAST searches on the 12 genes annotated as disease resistance proteins in the CS physical interval underlying Q*Lr*.gb-5D (TraesCS5D01G559100, TraesCS5D01G559200, TraesCS5D01G560500, TraesCS5D01G561100, TraesCS5D01G561200, TraesCS5D01G561300, TraesCS5D01G561400, TraesCS5D01G563400, TraesCS5D01G563500, TraesCS5D01G563600, TraesCS5D01G563700, and TraesCS5D01G564000). Similarly, the *Lr*1 from wheat cv ‘Glenlea’ (the source of the original resistant allele) [17] was used as a query to BLAST search the 12 CS sequences annotated as disease resistance proteins. The ‘Victo’ sequence showed no significant similarity to nine of these genes, while identity values ranging from 88.07% to 95.73% were found for TraesCS5D01G560500, TraesCS5D01G561300, and TraesCS5D01G561200, with the highest level of identity detected for TraesCS5D01G561200. The same results were obtained using the *Lr*1 from ‘Glenlea’ (Table S5, Supplementary File 1). On the basis of these results, TraesCS5D01G561200 could represent the CS allele of both the *Lr*1 from ‘Glenlea’ and the putative *Lr*1 from ‘Victo’.

The ‘Glenlea’ and ‘Victo’ sequences showed identity of 98.51% (E value 0.0). Fifty-one SNPs and 2 deletions were detected when the ‘Victo’ and ‘Glenlea’ sequences were aligned. Most of these polymorphisms (46 SNPs and 2 deletions) were restricted to a region of 443 bp, from position 4157 to position 4600 bp of the ‘Glenlea’ sequence (GeneBank Acc. N. ABS29034.1; Supplementary File 1).

**Figure 5:**
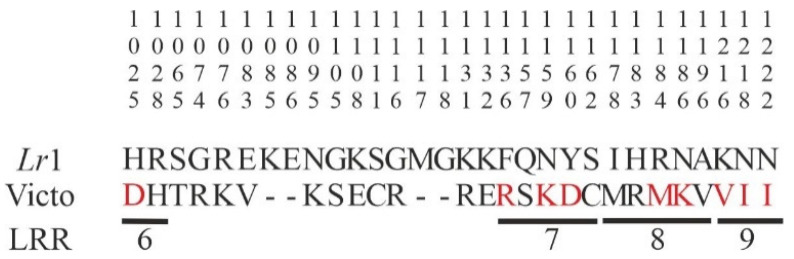
Polymorphisms between the *Lr*1 (GenBank ABS29034.1) and the ‘Victo’ protein sequences are reported from 1025 to 1222 of the ‘Glenlea’ amino acid sequence. The vertical number gives the amino acid positions in the *Lr*1 protein. Deletions are shown as dashes. The polymorphic sites detected in the leucine-rich-repeat (LRR) domain are indicated. In red are highlighted the amino acids that determine modifications in terms of physicochemical properties.

Alignment of the corresponding protein sequences showed 30 differences between the ‘Victo’ (1339 amino acids) and the ‘Glenlea’ *Lr*1 alleles (1344 amino acids). When these polymorphisms were analyzed with respect to the position of protein domains and structural motifs of *Lr*1 (GenBank ABS29034.1), no polymorphisms were identified in the NB-ARC and P-loop_NTPase domains; rather, the variations were restricted to the LRR structural motifs. In more detail, 15 amino acids changes are present in 4 LRR motifs, and 9 of these (at positions 1025, 1136, 1159, 1160, 1184, 1186, 1216, 1218, and 1222 of ‘Glenlea’ amino acid sequence) determine modifications in the physicochemical properties of the protein (Figure 5; Figure S1).

These results may suggest that Q*Lr*.gb-5D represents a new functional *Lr*1 allele.

### 2.5. In Silico Functional Analysis of QPm.gb-7A Candidate Resistance Genes

Analysis of the CS bread wheat genomic sequence underlying the Q*Pm.*gb-7A locus led to the identification of 80 annotated genes: of these, 12 were encoded functions compatible with a possible role in resistance to powdery mildew.

A total of nine sequences were annotated as disease resistance protein (TraesCS7A01G551900, TraesCS7A01G553600, TraesCS7A01G553700, TraesCS7A01G554200, TraesCS7A01G554400, TraesCS7A01G555200, TraesCS7A01G555300, TraesCS7A01G555400, and TraesCS7A01G557700), two as receptor-like protein kinase (TraesCS7A01G550000 and TraesCS7A01G550100), while TraesCS7A01G550200 encoded for a hexosyltransferase that could have a role in resistance as demonstrated for *Lr*67 in barley and wheat [22,59].

**Figure 6:**
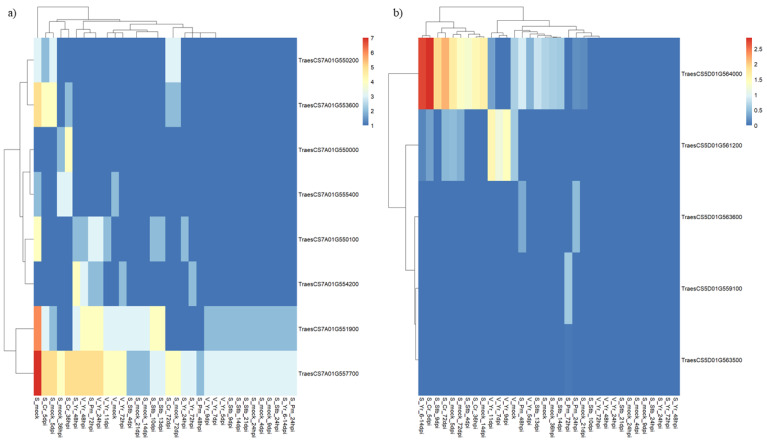
In silico expression analysis of selected candidate genes for powdery mildew (**a**) and leaf rust (**b**) resistance in response to biotic stresses in leaves and shoots. Seedling (S) and vegetative (V) developing stages are considered. Different time points, expressed as hour post inoculation (hpi) and days post inoculation (dpi), are indicated. Different diseases are considered: stripe rust (Yr), powdery mildew (Pm), crown rot (Cr), and septoria tritici blotch (Stb). The control condition is indicated as mock. Transcript abundances are expressed in log2 (tpm). Only genes whose expression profiles were available are reported. Data from Wheat Expression Browser (www.wheat-expression.com; accessed on 7 January 2021).

The in silico ExpVIP platform was used to retrieve the transcriptional profile of the 12 candidate genes listed above in response to biotic stresses in leaves and shoots. No expression was noticed for TraesCS7A01G553700, TraesCS7A01G55440, TraesCS7A01G555200, and TraesCS7A01G555300 (Figure 6; Table S6).

Seven genes (TraesCS7A01G551900, TraesCS7A01G550200, TraesCS7A01G555400, TraesCS7A01G550000, TraesCS7A01G553600, TraesCS7A01G557700, and TraesCS7A01G550100) were constitutively expressed. Three of them (TraesCS7A01G550200, TraesCS7A01G553600, and TraesCS7A01G550000) were found expressed only at seedling stage, while the remaining seem to be active regardless of developmental stages. The last remaining candidate gene (TraesCS7A01G554200) was reported as specifically expressed in response to pathogens in leaves and shoots at all developmental stages considered.

BLAST search against the CS genome of the *Pm*1a cloned sequence (GenBank accession ERZ1467246) identified TraesCS7A01G553600 as the *Pm*1a allele in CS. Considering the result of the absence of *Pm*1aSTS1 amplification (Figure 3), this region/gene, spanning for 2554 bp (from 726.46 Mbp to 726.47 Mbp; coverage 93%, Evalue = 0, and percentage of identity = 76%) can however be excluded from the list of putative candidate genes provided for the Q*Pm.*gb-7A locus.

When the in silico ExpVIP analysis was limited to powdery mildew RNA seq data, an increased expression during a late inoculation phase was observed for TraesCS7A01G550100, TraesCS7A01G554200, TraesCS7A01G557700, and TraesCS7A01G551900.

Expression analysis of cloned powdery mildew resistance genes from literature data indicate that all of them are responsive to pathogen infection. Cross-evidence about the most common expression pattern of cloned *Pm* genes with those of the candidates identified in the Q*Pm.*gb-7A physical interval in CS suggests that the role of candidates for the powdery mildew resistance governed by ‘Victo’ could be assigned to TraesCS7A01G550100, TraesCS7A01G554200 TraesCS7A01G557700, and TraesCS7A01G551900.

### 2.6. In Silico Functional Analysis of QLr.gb-5D Candidate Resistance Genes

The Q*Lr*.gb-5D interval includes 56 annotated genes, and 13 of them encode protein functions compatible with a possible role in resistance to leaf rust. In more detail, 11 genes (TraesCS5D01G559100, TraesCS5D01G559200, TraesCS5D01G560500, TraesCS5D01G561100, TraesCS5D01G561200, TraesCS5D01G561300, TraesCS5D01G561400, TraesCS5D01G563400, TraesCS5D01G563500, TraesCS5D01G563600, and TraesCS5D01G563700) were annotated as disease resistance-related, while TraesCS5D01G563300 and TraesCS5D01G564000 were annotated as receptor-like protein and Pleiotropic drug resistance ABC transporter, respectively. Among them, TraesCS5D01G561200 (currently TraesCS5D02G561200) corresponds to the putative *Lr*1 allele of CS, as discussed above.

Expression data were available only for five candidate genes (TraesCS5D01G561200, TraesCS5D01G564000, TraesCS5D01G563600, TraesCS5D01G559100, and TraesCS5D01G563500). Their transcription profiles in leaves and shoots revealed that two of them (TraesCS5D01G561200 and TraesCS5D01G564000) are expressed at different developing stages irrespective of pathogen presence, while TraesCS5D01G563600, TraesCS5D01G559100, and TraesCS5D01G563500 were induced by pathogen infection at different time points only at seedling stage. For the above-mentioned genes, generally, an increased expression level was related to both early and late infection time points (Figure 6; Table S7).

Although, to our knowledge, there are no data on the transcriptional response of the cloned *Lr* genes to leaf rust infection that could be used to further discriminate the candidates. The five genes in the region (TraesCS5D01G559100, TraesCS5D01G561200 TraesCS5D01G563500, TraesCS5D01G564000, and TraesCS5D01G563600) were identified as responsive to diverse pathogen infections. TraesCS5D01G564000 was responsive to all pathogens considered, and three candidate genes (TraesCS5D01G563600, TraesCS5D01G559100, and TraesCS5D01G563500) were responsive only to powdery mildew infection. Finally, TraesCS5D01G561200 showed a clear induction only upon rust infection, further substantiating it is a potent, transcriptional active *Lr*1 allele.

## 3. Discussion

In the present study, a new high-density wheat genetic map based on the Victo × Spada RIL population was combined with disease scores from two major wheat pathogens, which allowed the identification of three loci controlling wheat resistance to powdery mildew and leaf rust at seedling stage. Linkage analysis identified one QTL (Q*Pm.*gb-7A) located on chromosome 7A for powdery mildew resistance and two QTLs (Q*Lr*.gb-2B and Q*Lr*.gb-5D) on chromosomes 2B and 5D for leaf rust resistance.

### 3.1. A New Genetic Map for Triticum Aestivum

A new high-density genetic linkage map containing 8726 loci was developed in bread wheat using a RIL mapping population derived from the bread wheat cvs ‘Victo’ and ’Spada’. The number of polymorphic SNP markers (9828) between the parental lines is comparable to those detected using the same SNP platform in the RIL populations described by Gao et al. and Zhai et al., with 7514 and 11,646 polymorphic SNPs, respectively [36,60]. Both the order of the markers and the genetic length (3291.5 cM) of the Victo × Spada map are in agreement with those of previously reported maps in hexaploid wheat made with the iSelect 90k array. The number of markers on each genome was uneven since markers for the A (43.14%) and B (50.32%) genomes were more abundant than those for the D genome (6.54%). Consistently with previous studies [35,36,37,38], this finding can be attributed to the low level of polymorphism in the D genome of hexaploid wheat. The D genome is a recent evolutionary addition to the hexaploid wheat genome, and there has been limited gene flow between *A. tauschii* and *T. aestivum*, possibly explaining the low level of polymorphism [61]. Although the average density is high, more than one linkage group was obtained for some chromosomes (2D, 3A, 4A, 4D, 6D, 7A, 7B, and 7D). The presence of large gaps and chromosome regions with low marker density has been described in several bread wheat maps developed using the iSelect 90K array [35,36,37,38,62,63] and could be explained by a lack of polymorphic markers, limited sequence variation, or extended identity by descent between the parents of the mapping population.

### 3.2. Identification of Powdery Mildew Resistance QTL in Hexaploid Wheat

The Q*Pm.*gb-7A resistance locus identified in the present work spans approximately 6.35 Mbp between marker IWA6833 and marker IWA5904 on chromosome 7AL and resides in a genomic region enriched in powdery mildew resistance genes. The physical interval retrieved for Q*Pm.*gb-7A compared with those of previously mapped powdery mildew resistance genes allowed the identification of positional overlapping between Q*Pm.*gb-7A and *Pm*59, *Pm*1, *mlm*80, *ml*IW72, *ml*IW172, *HSM*1, *Pm*G16, *Pm*Tb7A.2, and *mlm*2033 [39,40,41,42,43,44,45,46,47,48,49,50,51,52,53,54,55]. The tight linkage of these genes would suggest that they are likely members of a resistance gene cluster. Clustering of resistance genes is commonly observed in plant genomes and has been described in cloned resistance gene loci [64]. To our knowledge, what clearly differentiated Q*Pm.*gb-7A from *Pm*59, *Pm*1, *ml*IW72, *ml*IW172, *HSM*1, *Pm*G16, *mlm*80, *Pm*Tb7A.2, and *mlm*2033 is the origin of these genes. Indeed *ml*IW72, *ml*IW172, *HSM*1, and *Pm*G16 were identified in *T. dicoccoides*, *mlm*80 and *Pm*Tb7A.2 in *T. boeticum,* and *mlm*2033 in *T. monococcum*. The only three known regions previously identified in *T. aestivum* are represented by *Pm*1, *Pm*59, and *ml*RD30 that were detected in the Canadian wheat *cv* Axminster, in the Afghan wheat landrace PI 181356, and in the breeding line RD30, respectively [48,49,50]. Absence in ‘Victo’ of the *Pm*1aSTS marker diagnostic for the *Pm*1 in the bread wheat line [33] allowed to conclude that Q*Pm.*gb-7A is different from *Pm*1.

Clearly, the telomeric region of chromosome 7A appears to be rich in powdery mildew resistance genes. Allelism analysis would clarify the relationships among these genes. Q*Pm.*gb-7A explained the effective powdery mildew resistance harbored by ‘Victo’ and represents a useful source of disease resistance for powdery mildew resistance breeding in hexaploid wheat, supported by the SNP markers here identified as tightly associated to the resistance locus. Search for candidate genes in the Q*Pm.*gb-7A physical region in CS identified 12 genes encoding proteins with possible disease resistance function against powdery mildew. Consistent with previous transcriptional studies of cloned *Pm* genes, two candidates, TraesCS7A01G550100 and TraesCS7A01G554200, are activated in response to powdery mildew infection. The results here obtained set a solid basis for further molecular isolation of the mildew resistance gene underlying the Q*Pm.*gb-7A.

### 3.3. Identification of Leaf Rust QTLs in Hexaploid Wheat

In this work, a major QTL controlling leaf rust resistance (Q*Lr*.gb-5D) derived from the bread wheat *cv* ‘Victo’ was mapped on the long arm of chromosome 5D. This resistance locus explains nearly 60% of the phenotypic variation for leaf rust resistance conferred by the resistant parent, as evaluated using the IT and RDS scores. This locus represents a useful source of leaf rust resistance in hexaploid wheat, supported by closely associated SNP markers identified in this work.

Literature data indicate that three leaf rust resistance genes are located on the long arm of chromosome 5D: *Lr*1, shown to be present in many wheat *cvs* [10,11] and *A. tauschii* accessions e.g., [65], *Lr*Syn137 from *A. tauschii* [57], and *Lr*RB88 from common wheat [58]. Two molecular markers RGA567 and WR003 have been proposed as functional markers to identify the *Lr*1 gene. Qi et al. [58] described the leaf rust resistance gene *Lr*LB88 on chromosome 5DL as co-segregating with *Lr*1 (WR003 marker) but also showed a reaction pattern to 13 Chinese rust pathotypes that was clearly distinct with respect to *Lr*1 [58]. Mohler et al. [57], using the functional *Lr*1 marker RGA567 that mapped proximal to *Lr*Syn137, provided evidence indicating that *Lr*Syn137 and *Lr*1 are different rust resistance genes [57].

Co-positional relationships on the CS genome sequence were identified for Q*Lr*.gb-5D and the position of the *Lr*1 leaf rust resistance gene. Sequencing of the *Lr*1 allele from ‘Victo’ highlighted strong sequence identity (98.51%) with the *Lr*1 gene cloned from the wheat *cv* ‘Glenlea’.

The polymorphisms identified were concentrated within a region of 443 bp lying in the LRR domain of the encoded NLR protein. Moreover, alignment of the amino acid sequences showed perfect identity until position 1025, after which differences in 26 amino acids were found in the LRR region (from aa 1025 to aa 1222 of the ‘Glenlea’ sequence; Figure 5; Figure S1). None of these amino acid changes is predicted to make the ‘Victo’ *Lr*1 protein unfunctional or truncated.

Analysis of the CS annotated sequences in the Q*Lr*.gb-5D region identified 13 genes encoding proteins with possible roles in resistance to leaf rust. Among these, TraesCS5D01G561200 showed the highest level of sequence identity with *Lr*1 and with the ‘Victo’ allele of *Lr*1. In silico analysis of the expression of this gene using the ExpVIP platform indicated that TraesCS5D01G561200 is responsive to pathogen infection.

Taken together, these results may raise the possibility that Q*Lr*.gb-5D represents a new *Lr*1 allele in the bread wheat *cv* ‘Victo’. Such a hypothesis could be tested in the future using functional mutagenesis assays in ‘Victo’, or by means of the transgenic line expressing the *Lr*1 ‘Victo’ allele.

## 4. Materials and Methods

### 4.1. Genetic Materials

Two bread wheat cultivars, ‘Victo’ (pedigree: NK-79-W-810/W-0010-E//W-1051-A) and ‘Spada’ (pedigree: TREBBO/KANSAS//(TR.TG) PSEUDOCERVINUM), were crossed, and 128 F7–8 RILs were developed by single-seed descent. For DNA extraction, one leaf of each line was ground using the Retsch MM300 Mixer Mill instrument (Newtown, PA, USA), then the Wizard Magnetic 96 DNA Plant System (Promega Italia, Milano, Italy) kit was used for DNA extraction and purification following the manufacturer’s instructions. All the germplasm was developed and maintained by CREA-Research Centre for Genomics and Bioinformatics in Fiorenzuola d’Arda (Piacenza, Italy).

### 4.2. Phenotypic Evaluation

Evaluations for the powdery mildew responses were performed using three different *Blumeria graminis* f. sp. *tritici* (*Bgt*) isolates, two from Switzerland (*Bgt*_96224 and *Bgt*_94202) and one from UK (*Bgt*_JIW2) [66]. These isolates, with differential virulence specificities for many known powdery mildew resistance genes, were first used to evaluate the response of the parental lines. Results of this preliminary screen indicated that two of these isolates (*Bgt*_96224 and *Bgt*_JIW2) provided a suitable level of discrimination of the resistant vs. susceptible responses between the parents. One of these two isolates, *Bgt*_JIW2, was then used to screen the RIL population. Regarding *P. triticina* (*Pt*), two isolates, *Pt*_Jerez05 and *Pt*_VMC03, were tested: both showed a clear discrimination in the resistant/susceptible response of the parents, with ‘Victo’ being resistant, while ‘Spada’ was susceptible at the first, fourth, and fifth leaf stages. The isolate *Pt*_VMC03 was selected to analyze the RIL population. *Pt*_VMC03 is a monosporic isolate derived from a rust population collected at Villamanrique de la Condesa, Sevilla, Spain, in 2003 [67]. For both pathogens, the criterion for choosing the isolates to be used to screen the RILs was based on maximizing the responses at the level of ‘Victo’ and ‘Spada’.

Powdery mildew and leaf rust reactions were evaluated in controlled conditions at seedling stage. The seedling resistance assays for powdery mildew [68] were evaluated using a detached leaf segment method, and six independent replicates were used for each RIL line. The leaf rust experiment consisted of 3 pots (replicates) per RIL and 5 plants per pot, using the procedure reported in Desiderio et al. [67].

The 128 RILs and their parents were evaluated, and the infection type (IT) for each pathogen and disease severity (DS) for leaf rust were recorded. IT was score with visual evaluation of the sporulation intensity and expressed in a scale from 0 (fully resistant) to 9 (fully susceptible). Reactions to leaf rust and powdery mildew (IT) were scored 14 and 10 days after inoculation, respectively [67,68]. DS was visually estimated as the percentage of leaf area covered by rust. To calculate a balanced average, the original disease scores were converted into relative disease severity (RDS) values, by setting the disease value of the susceptible parent ‘Spada’ equal to 100.

### 4.3. Statistical Analysis

Frequency distributions of the phenotypic data were tested for normal distribution to estimate the complexity of genetic control of the traits. Analysis of variance (ANOVA) of disease traits was performed to test the significance of differences between RILs and replications using JMP v. 7 software [69] (SAS Institute, Milano, Italy). The effects of replications and genotypes were accounted in the model. The values of variance obtained from the ANOVA were used to calculate broad sense heritability according to Nyquist and Barker [70]. Correlations between traits were estimated using the Spearman coefficient.

### 4.4. Linkage Analysis

Single-nucleotide polymorphism (SNP) molecular markers were used to analyze the parental lines and the RILs. Genotyping was performed at the Trait Genetics Laboratory (Gatersleben, Germany) with the Infinium iSelect 90K wheat SNP BeadChip array (Illumina Inc., San Diego, CA, USA) carrying 81,587 functional markers [62]. SNP markers with ambiguous SNP calling between parents and/or with a negative hybridization response in most lines were removed from the data set; after this check, 67,799 high-quality SNP markers were retained.

Linkage analysis was performed using the CARTHAGENE software [71] with a LOD score threshold of 9.0, maximum distance of 20 cM, and the Kosambi mapping function to calculate map distances [72]. The obtained linkage groups were assigned to chromosomes by comparing markers of the generated maps to previously published high-density bread wheat maps [35,36,37,38,62,63]. Within each linkage group, the best order of markers and the genetic distances were found using different CARTHAGENE’s functions: build, greedy, flips, and polish. All mapped markers were tested for the expected 1:1 segregation ratio using a chi-square (χ^2^) goodness-of-fit test.

### 4.5. QTL Analysis

QTL mapping was conducted with the R/qtl package of the R statistical computing software [73]. For each trait, an initial QTL scan was performed using simple interval mapping with a 1 cM step [74], and the position of the highest LOD was recorded. A genome-wide significance level of 5% was calculated after 1000 permutations [75], and LOD threshold greater than or equal to 3.2 was used to declare a QTL. Then, the position and the effect of the QTL were determined using the multiple imputation method by executing the “sim.geno” command, followed by the “fitqtl” command [76]. To search additional QTLs, the “addqtl” command was used. If a second QTL was detected, the “fitqtl” was used to test a model containing both QTLs and then the interaction effect. If both QTLs remained significant, the “refineqtl” command was used to re-estimate the QTLs’ positions based on the full model including both loci. QTLs interactions were studied, and the significant locus combinations are reported based on F-measure. The additive effects of QTLs were estimated as half of the difference between the phenotypic values of the respective homozygotes. The confidence interval (CI) of each QTL was determined as proposed by Darvasi and Soller [77].

### 4.6. Analysis of Physical Regions Carrying QTLs Related to Disease

The major QTLs identified in the present study were projected on the *T. aestivum* reference genome sequence (*cv* ‘Chinese Spring’; CS) to define their physical confidence interval [78]. Peak markers and flanking markers corresponding to the confidence intervals (CIs) were located on the reference genome using BLAST search matches of the corresponding SNP flanking sequences. This information was then used to compare the QTLs identified in this work with known genes/QTLs, previously identified in wheat [10,11]. For this purpose, the physical interval of known genes was defined using marker information as a query to perform the BLAST searches against the CS reference genome.

The physical regions underlying the QTLs were inspected to identify candidate genes based on their functional annotation. Moreover, if functional markers were available for known genes, they were tested on the parental lines.

Finally, the ExpVIP platform (Wheat Expression Browser, www.wheat-expression.com) was employed to analyze the transcriptional profile of the candidate genes underlying the identified QTLs, and we assessed their activation in response to biotic stresses using RNA sequencing data available on the platform. In detail, we considered for the analysis leaves and shoots as tissues and seedling and vegetative developing stages. Transcript abundances are expressed in log2 (transcript per million, tpm) [79]. In this work, only the experimental designs addressing responses to pathogens were considered.

## Figures and Tables

**Table 1:** Parental lines reaction responses (expressed as infection type) to different isolates of *Puccinia triticina* (*Pt*) and *B. graminis* (*Bgt*) tested.

	*Pt*_Jerez05	*Pt*_VMC03	*Bgt*_96224	*Bgt*_94202	*Bgt*_JIW2
Victo	0	0	4.3	8	3.1
Spada	9	9	7.8	9	8

**Table 2:** The observed segregation ratio among resistant (R) and susceptible (S) lines.

Trait/Disease	R:S	Ratio	*p* ^1^
IT Leaf Rust	83:38	1:1	4.6 × 10^−5^
IT Powdery Mildew	59:61	1:1	0.797894

^1^*p* value < 0.05 indicating that the observed segregation ratio is significantly different from the expected segregation ratio at 95% level of confidence.

**Table 3:** Distribution of molecular markers in the chromosomes and homeologous groups of the Victo × Spada bread wheat map. Information on Kosambi centiMorgan (cM) length of the maps across the bread wheat chromosomes, and chromosome-based mean inter-locus separation (cM/marker) is also provided.

Chromosome_ Linkage Group	Total Marker	Total Marker (No Co-Segregant)	cM	cM/Marker ^1^	cM/Marker ^2^
1A_1	704	102	159.2	0.23	1.56
1B_1	801	115	186.1	0.23	1.62
1D_1	146	44	100.3	0.69	2.28
2A_1	422	63	167	0.40	2.65
2B_1	841	113	199.3	0.24	1.76
2D_1	21	8	9.7	0.46	1.21
2D_2	40	19	85.8	2.15	4.52
2D_all	61	27	95.5	1.57	3.54
3A_1	383	79	167.6	0.44	2.12
3A_2	68	14	38.4	0.56	2.74
3A_all	451	93	206	0.46	2.22
3B_1	708	116	228.7	0.32	1.97
3D_1	34	6	19.1	0.56	3.18
4A_1	171	60	161.5	0.94	2.69
4A_2	84	16	15.7	0.19	0.98
4A_all	255	76	177.2	0.69	2.33
4B_1	305	62	150.7	0.49	2.43
4D_1	7	6	12.4	1.77	2.07
4D_2	6	6	9.3	1.55	1.55
4D_3	3	3	10.1	3.37	3.37
4D_all	16	15	31.8	1.99	2.12
5A_1	578	146	323.2	0.56	2.21
5B_1	774	139	233	0.30	1.68
5D_1	95	18	104.2	1.10	5.79
6A_1	644	75	157.7	0.24	2.10
6B_1	448	78	197.1	0.44	2.53
6D_1	22	7	22.5	1.02	3.21
6D_2	136	14	14.2	0.10	1.01
6D_all	158	21	36.7	0.23	1.75
7A_1	611	115	198	0.32	1.72
7A_2	99	15	20	0.20	1.33
7A_all	710	130	218	0.31	1.68
7B_1	423	84	131.3	0.31	1.56
7B_2	91	15	49.3	0.54	3.29
7B_all	514	99	180.6	0.35	1.82
7D_1	13	5	12.2	0.94	2.44
7D_2	48	25	107.9	2.25	4.32
7D_all	61	30	120.1	1.97	4.00
Total	8726	1568	3291.5	0.38	2.11
Genome					
A	3764	685	1408.3	0.37	2.06
B	4391	722	1375.5	0.31	1.91
D	571	161	507.7	0.89	3.15
Group					
1	1651	261	445.6	0.27	1.71
2	1324	203	461.8	0.35	2.27
3	1193	215	453.8	0.38	2.11
4	576	153	359.7	0.62	2.35
5	1447	303	660.4	0.46	2.18
6	1250	174	391.5	0.31	2.25
7	1285	259	518.7	0.40	2.00

^1^ Calculated considering all the markers. ^2^ Calculated considering only the no co-segregating markers.

**Table 4:** QTLs and their interactions detected in the Victo × Spada segregating population for powdery mildew and leaf rust resistance. Chromosomes (Chr), peak marker, LOD scores, percentages of phenotypic variance explained (*R*^2^), estimated additive effects, and the confidence interval (CI) were also provided.

QTL	Trait Disease	Chr	Peak Marker	cM	LOD	*R*^2^ (%)	Additive Effect ^1^	CI
Q*Pm.*gb-7A	IT powdery mildew	7A	IWB55071	14.8	60	90	−3.8	14.1–15.5
Q*Lr*.gb-2B	IT leaf rust	2B	IWB32376	113.9	10.5	15.2	−1.16	109–118.1
Q*Lr*.gb-5D	IT leaf rust	5D	IWB11400	104.2	28.1	59.3	−2.43	103.1–105.3
Q*Lr*.gb-2B*Q*Lr*.gb-5D	IT leaf rust				4.74	6.11	0.93	
Q*Lr*.gb-2B	RDS leaf rust	2B	IWB32376	113.9	11.2	16.2	−14.153	109.9–117.8
Q*Lr*.gb-5D	RDS leaf rust	5D	IWB11400	104.2	28.4	59.3	−28.599	103.1–105.3
Q*Lr*.gb-2B*Q*Lr*.gb-5D	RDS leaf rust				5.1	6.51	11.335	

^1^ The negative value indicates that the allele of the gene from ‘Victo’ positively contributed to the resistance or reduced the severity of disease.

**Table 5:** Genetic and physical intervals identified for the major QTLs identified for powdery mildew and leaf rust resistance.

QTL	Chr	Genetic Interval (cM)	Start (cM)	End (cM)	Mbp	Start (bp)	End (bp)
Q*Pm.*gb-7A	7A	1.41	14.1	15.5	6.35	724,084,703	730,433,215
Q*Lr*.gb-5D	5D	2.15	103.1	105.3	2.49	560,228,250	562,726,656

## Data Availability

https://zenodo.org/search?page=1&size=20&q=4534102 (accessed on 11 February 2021).

## Supplementary Materials

The following are available online at https://www.mdpi.com/1422-0067/22/6/3109/s1, Figure S1: Alignment of amino acid sequences of *Lr*1 from ‘Glenlea’ and ‘Victo’, Table S1: Analysis of variance, Table S2: Victo × Spada linkage map, Table S3: Origins of powdery mildew resistance genes previously mapped to the long arm of chromosome 7A, Table S4: Primer pairs developed to amplify the *Lr*1 gene, Table S5: BLAST results obtained using the ‘Victo’ and the cloned *Lr*1 sequences (GeneBank Acc. N. ABS29034.1) as a query towards the Chinese Spring reference genome, Table S6: Transcriptional profile of candidate genes selected for powdery mildew in response to biotic stresses in leaves and shoots retrieve from the ExpVIP platform, Table S7: Transcriptional profile of candidate genes selected for leaf rust in response to biotic stresses in leaves and shoots retrieve from the ExpVIP platform, Supplementary File 1: Nucleotide sequence alignments.
