# Supplementary material for: Characterization of the Resistance to Powdery Mildew and Leaf Rust Carried by the Bread Wheat Cultivar Victo
Source: Int J Mol Sci. 2021 Mar 18;22(6):3109. doi: 10.3390/ijms22063109 (PMC8003046; doi:10.3390/ijms22063109)
Supplement: Supplementary file 1 [file ijms-22-03109-s001.zip › Supplementary_Files/Supplementary File 1.docx]

Supplementary File 1: Nucleotide sequence alignments between *Lr*1 Victo allele and a) TraesCS5D01G560500, b) TraesCS5D01G561200, c) TraesCS5D01G561300 and d) the *Lr*1 Glenlea cloned allele (GeneBank Acc. N. ABS29034.1).

a)

1 130

Lr1_Victo_allele GCCCACACGC CCAAGCGTGA TGGCATCTTC CATATGTTTT ATCGGATGTT TGTTTATCTA CAAAACCTTC CACTCTCCCT AAAAAACCAA GGACTTGGAT GGTTCCGAAA TTTCCATATA AATGCAACCC

TraesCS5D01G560500

Consensus .......... .......... .......... .......... .......... .......... .......... .......... .......... .......... .......... .......... ..........

131 260

Lr1_Victo_allele GTGAAAATGT TGAGATATGT TCTTACACAT GCTCACCGCC AGTGGCAGAT CCGGGACCCA TGCCGGTGGG GCCTAGGCCT AGGGCATGAG AATGATTTAC TTCGTTGACC GCAGCTTATT GAGCACTGTA

TraesCS5D01G560500

Consensus .......... .......... .......... .......... .......... .......... .......... .......... .......... .......... .......... .......... ..........

261 390

Lr1_Victo_allele GCTGCACTGT AGCGCCTGGG GCCTGGGCTT GGCCCAATCC TGGGTCCGCC CCTGCTCACC GCCTAAAGGA GATACAAAAG AAATATTCTA AATGGCAGAA AAATCTTGGG ATTTTGAAAT CTAAATCATG

TraesCS5D01G560500

Consensus .......... .......... .......... .......... .......... .......... .......... .......... .......... .......... .......... .......... ..........

391 520

Lr1_Victo_allele TACTCACTCC GTCCCAAAAT AAGTGTTTCA ACTTTAGTAT AGTTTTGTAC TAAAGCTAAT ACAGAGTTGA GACACTTATT TTGAGACGGG AGGATATGTA TTTTCTGTTA TGTTTCGGAT TGTATCTCGA

TraesCS5D01G560500

Consensus .......... .......... .......... .......... .......... .......... .......... .......... .......... .......... .......... .......... ..........

521 650

Lr1_Victo_allele GAAGAATGTT CTTATATTAT GGAACGGGGA GTCTTATTGT TTGATTAATT ATTAATAAAG CCGACATTCT GTAAAGAAAC CTTTCAGCAG GGGTGTATTC GTAAAAGTTT TGCATTACCG GCTTGGGTCC

TraesCS5D01G560500

Consensus .......... .......... .......... .......... .......... .......... .......... .......... .......... .......... .......... .......... ..........

651 780

Lr1_Victo_allele CAATAATTTG TATCCATAGG TTGGTCAAAC AGCTCAAACC ATACCAACCA AGCAAGCAAG CAATCCCCAC ACCGCTAGAG CTACAGCTTC -CACCGGCGT CAAAAGAAAA CACCAATTAT TGCCCCGGCG

TraesCS5D01G560500 ATGGA GGTGGCTCTC GGCTCGGCGG CCTCGGTCCT CG---GCAAG GTGTGG--AC GACGCT-GTC CGACACCGTG GTGGCGGCGT ACGTGGACAG CCTCCAGCTC GGCCACAACT

Consensus .......... .....ATaGa gggGgCaaaC aGCTCaaacc acaCcaaCCa aG...GCAAG caaTcc..AC aaCGCT.Gac CgACAcCgTc .cacCGGCGT aaaaaGAaAa CacCaAgcac gGCCaCaaCg

781 910

Lr1_Victo_allele ACTCGACGTC CGGCGGCCGG CGGAGTGCAG ATCTACGGCT GTGCGGTGGC GTGGGCAGTT GGGGAAGAGT ACCCTGTCCT TGCTCCCCAC ACGACACCGC TCCCCGGCGG CGTTGACCGA GCTCGGAGCT

TraesCS5D01G560500 CCCAGCAGAT CAGGGATAAG CTGC-TGCAC GCGCAAGGCC -TGCTGCACA ACGCCCAG-- GGGCAGGGGA GCCATAACCC TGG--CCTGC AGG-----GG TTGCTGGAGA AGCTGAGCAG GGACGCCGAC

Consensus aCcaGaaGac CaGcGacaaG CgGa.TGCAc acccAaGGCc .TGCgGcaca acGccCAG.. GGGcAaGaGa aCCaTaaCCc TGc..CCcaC AcG.....Gc TccCcGGaGa aGcTGAcCaa GcaCGcaGac

911 1040

Lr1_Victo_allele GAGGCGAGGC GGCCGGCGGA GTGCAGATGG CGGCGGCTCT CGGCTCGGCG GCCACGCTCC TCGGCAAGGT GTTCACTATG CTGTCCGCCG CCCCGGTGGC GG---CGTAC GTGGACAGCC TGGAGCTCGG

TraesCS5D01G560500 CAGGC-AGAG GACCTGCTG- --GATGAGGT CCACTACTTC CAGATCCATG AC-AGGCTCC ACGGCAC--- --CAACTATG CCA-CCACCA CCCAAGAGGC CGGCCCGGAT CTGGACAGCC TTGTCCACCA

Consensus cAGGC.AGac GaCCgGCgG. ..GaaGAgGg CcaCgaCTcc CaGaTCcacG aC.AcGCTCC aCGGCAa... ..caACTATG Cca.CCaCCa CCCaaGaGGC cG...CGgAc cTGGACAGCC TgGacCaCca

1041 1170

Lr1_Victo_allele CCACAA-CTC CCAGCAGATC AGGGCCAAGC TCGCGCACAC GCGAGGCCTG CTGCACAACG CCCAAGCCCA GGTGAGCGAC GTCGGCCACA ACCCTG--GA CTGCAGGAGC TGCTGCCGGC GCTGAGCAGG

TraesCS5D01G560500 TCAAGCTCTC CACGCCGGTA CTGCTCT--- TCGC-CACAC CCTAGGCA-G CTTAGTCCAG TGCTTTTCT- --TGCTCGCC TACACCCAAG ACCAAGAGGG ATGGAGGTGA TGATGCTGCT GCTGTTGCAC

Consensus cCAaaa.CTC CaaGCaGaTa agGccCa... TCGC.CACAC cCgAGGCa.G CTgaacaaaG ccCaagcCc. ..TGagCGaC gaCacCCAaa ACCaaG..Ga aTGcAGGaGa TGaTGCcGcc GCTGagcaac

1171 1300

Lr1_Victo_allele AACGCCGACG AGGCGGAGGA TCTGCTGGAT GAGCTCCACT ACTT--CCAG ATCCATGACA GGCTCCACGC CACCAACTAC GCCGCCACCC AGGCCAATTT TCTCCGTCAT GCTCGCAATG CTCTCCGCCA

TraesCS5D01G560500 CACGCCG-CG TCACCAAG-A GCACCT---- -TGCTCCACT GCTTTTCTTG CTCCTGTGCA --CCCAAGAG CAAGAGGAAT GCTGCTGCTG TTGC-TGTTG CCGGCGT--T GGTGGTGATG CT-GCTGCTG

Consensus aACGCCG.CG acaCcaAG.A gCacCT.... .aGCTCCACT aCTT..CcaG aTCCaggaCA ..CcCaAcac CAacAacaAc GCcGCcaCcc agGC.aaTTg cCgcCGT..T GcTcGcaATG CT.gCcGCca

1301 1430

Lr1_Victo_allele CACTGCCACC AGCAGCTGGG CCGCATGCTT TTCTTGTTCC TCTGCACAAG ATGATAGTGA TTCTACCAGT GGGGATGATG AGTTACGTTT CCACCGAGTG ATCTTCTCCA GAAAATTCAA GTCGGTGTTA

TraesCS5D01G560500 C--TGCTGCC GGACTCA--- CCAACTCCAA CTCCAACTCT GCTAGTGCTG ATGATGGTGA TAC-ACCG-- ---------- -----CGTTT TGACAGAGTG TTCATGTCCA GAGAAATCAA GTCCGTGTTA

Consensus C..TGCcaCC aGaagCa... CCaaaTcCaa cTCcaacTCc gCTacacaaG ATGATaGTGA TaC.ACCa.. .......... .....CGTTT ccACaGAGTG aTCaTcTCCA GAaAAaTCAA GTCcGTGTTA

1431 1560

Lr1_Victo_allele CAGGACATGC AGACGCACTG TGATTCCGTC TCTGATTTGC TCGGCACTAT CCCAACCAGC AGCAT---GC CAGTTGCTGT ACACC----- -GGCCGCAGA TTGGATCCAC AATTATACAA GATACATTGT

TraesCS5D01G560500 CAGGACATGC AGTCCCACTG TGATTCCGTC TCCAATTTGC TTTGCACTAT CCCAACCAGC AGCAGCAGGG CAGTGGCAGT CGTCCAAGAT CGGCCTCAGA CAGCTTCCAT GATTATACAA GATACATTGT

Consensus CAGGACATGC AGaCcCACTG TGATTCCGTC TCcaATTTGC TcgGCACTAT CCCAACCAGC AGCAg...Gc CAGTgGCaGT acaCC..... .GGCCgCAGA caGcaTCCAc aATTATACAA GATACATTGT

1561 1690

Lr1_Victo_allele ATGGCAGGAG ACACACTTTT GAGGAAACTG TCAACCGTAT CTTCAGCTGC AAACACCCTG ------TTTC TGTTCTTCCT ATAGTTGGTC CAGGGGGTAT TGGAAAGACA ACTTTTGCTC AACATCTGTA

TraesCS5D01G560500 ATGGCAGGAG ACACACTTTT GAGGAAACTG TCAACCGTAT CACCACTGTC ACACACACTG AGACTGTTTC TGTTCTTCCT ATAGTTGGTC CAGGGGGTAT TGGAAAGACA ACTTTCACCA CTCACCTGTA

Consensus ATGGCAGGAG ACACACTTTT GAGGAAACTG TCAACCGTAT CacCAccggC AaACACaCTG ......TTTC TGTTCTTCCT ATAGTTGGTC CAGGGGGTAT TGGAAAGACA ACTTTcaCca aaCAcCTGTA

1691 1820

Lr1_Victo_allele TAATGATGCA AGGACTGAAG AGCACTTCCA AGTCAGGGTC TGGGTGTGTG TATCCACTGA TTTCAATGTG CTTAAGCTCA CCAGGGAGAT CCTTGCCTGC ATACCTGCAA CTGAAGAAGG AGGAAGCAGC

TraesCS5D01G560500 CAATGACGCA AGGACTCAAG ACCACTTCCA AGTCAGGGTT TGGGTATGTG TATCCACTGA TTTCGATGTG CTTAAGCTCA CCAGGGAGAT CCTTGGCTGC ATACTTGCAA CTGAAGGAGC AGGTAGCAGC

Consensus cAATGAcGCA AGGACTcAAG AcCACTTCCA AGTCAGGGTc TGGGTaTGTG TATCCACTGA TTTCaATGTG CTTAAGCTCA CCAGGGAGAT CCTTGcCTGC ATACcTGCAA CTGAAGaAGc AGGaAGCAGC

1821 1950

Lr1_Victo_allele AGTGTTGCAA ATGAAACAAC CAATTTAGAT CATCTTCAGA GATCCATTGT GCGCCGTCTC AAGTCCAAGA GGTTTCTAAT TGTCTTGGAC GATATATGGA AATGTGACAG TCAGGATCAG TGGAAAACCT

TraesCS5D01G560500 GGTGTTGCAA ATGAAACAGC CAATTTAGAC CAGCTTCAGA AATCCATAGC AGAACGTCTC AAGTCCAAGA GGTTTCTAAT TGTCTTGGAT GATATATGGA AATGTGACAG TCAGGATCAG TGGAAAACCC

Consensus aGTGTTGCAA ATGAAACAaC CAATTTAGAc CAgCTTCAGA aATCCATaGc acaaCGTCTC AAGTCCAAGA GGTTTCTAAT TGTCTTGGAc GATATATGGA AATGTGACAG TCAGGATCAG TGGAAAACCc

1951 2080

Lr1_Victo_allele TGTTAGCTCC CTTCACAAAG GGGGAAACCA AAGGAAGCAT GCTACTTGTC ACAACTCGAT TCCCAAAGCT AGCACAAATG ATGGAAACAA TTGATCCACT AGAGCTGCTA GGTTTGGAGT CTAATGACTT

TraesCS5D01G560500 TGGTAGCTCC GTTCACAAAG GGGGAAAGCA AAGGAAGTAT GCTACTTGTC ACAACTCGAT TCCCAAAGGT AGCAGACATG GTGAAAACAG TTGATCCACT AGAGCTACGA GGTTTGGAGT CTAATGACTT

Consensus TGgTAGCTCC cTTCACAAAG GGGGAAAcCA AAGGAAGcAT GCTACTTGTC ACAACTCGAT TCCCAAAGcT AGCAcAaATG aTGaAAACAa TTGATCCACT AGAGCTaCgA GGTTTGGAGT CTAATGACTT

2081 2210

Lr1_Victo_allele CTTCACATTC TTTGAAGCAT GTATATTTGG TGAAGACAAC AAGCCAGAGC ATTTCGAAGA TGAGTTAGCT GGTATTGCAC AAAAAATTGC AGACAAGCTA AAGGGTTCCC CGCTGGCAGC CAAAACAGTT

TraesCS5D01G560500 CTTCACATTC TTTGAAGCAT GTATATTTGG TGAAGAAGAA AAGCCCGAGC ATTACCAAGA TGAGTTTGCT GGTATTGCAC GAAAAATTGC AAACAAGCTA AAGGGTTCCC CGCTTGCAGC CAAAACAGTT

Consensus CTTCACATTC TTTGAAGCAT GTATATTTGG TGAAGAaaAa AAGCCaGAGC ATTaCcAAGA TGAGTTaGCT GGTATTGCAC aAAAAATTGC AaACAAGCTA AAGGGTTCCC CGCTgGCAGC CAAAACAGTT

2211 2340

Lr1_Victo_allele GGTAGACTAT TGCACAAGGA CCTTTCTCAG AAACATTGGA ATGGAGTTCT TGAAAAGCAT CAGTGGCTAA AGCAGCAAAA TAATGATGAT ATCATGCCAT CTTTAAAAAT TAGCTATGAT TGCCTCCCTT

TraesCS5D01G560500 GGTAAGCTAC TGCATAAGCA CCTTTCTCAG GAACATTGGA GTGGAGTTCT TGAAAAGCAT CAGTGGCTAA AGCAGCAAGA CAATGATGAT ATCATGCCAT CTTTAAAGAT CAGCTACAAT TGCCTCCCTT

Consensus GGTAaaCTAc TGCAcAAGcA CCTTTCTCAG aAACATTGGA aTGGAGTTCT TGAAAAGCAT CAGTGGCTAA AGCAGCAAaA cAATGATGAT ATCATGCCAT CTTTAAAaAT cAGCTAcaAT TGCCTCCCTT

2341 2470

Lr1_Victo_allele TTGATCTGAA GAAATGTTTT TCCTATTGTG GCCTTTTCCC CGAAGATCAT TGGTTTACTT CTTCAGAAAT CAATCATTTC TGGGTTGCAG TAGGCATCAT AGACTCTGAT CACCAAGCCG ATAGGAATTA

TraesCS5D01G560500 TTGATCTGAA GAAATGTTTT TCCTATTGTG GCCTTTTCCC TGAAGATTAT CCGTTTACTT CTTCAGAAAT CAATCATTTC TGGGTTGCAA TAGGCATCAT AGACTCTGAT CACCAAGCCA ATAGGAATTA

Consensus TTGATCTGAA GAAATGTTTT TCCTATTGTG GCCTTTTCCC cGAAGATcAT ccGTTTACTT CTTCAGAAAT CAATCATTTC TGGGTTGCAa TAGGCATCAT AGACTCTGAT CACCAAGCCa ATAGGAATTA

2471 2600

Lr1_Victo_allele CCTGGAAGAG CTAGTGGACA ATGGTTTTCT CATGAAGAAG AAAGAGTATT ATTTGGAT-- -GATCGATGC AAACAAAAG- --GAATTTGA TTGCTATGTA ATGCATGATT TAATGCATGA GCTATCTAAG

TraesCS5D01G560500 CCTGGAAGAA CTAGTGGACA ATGGTTTTCT CATGAAGAAG AAAAAGTATT ATTTGGATCA TGATCGATAC AAACAAAAGA AGGAAATTGA TTGCTATGTA ATGCATGATT TAATGCATGA GCTATCTAAG

Consensus CCTGGAAGAa CTAGTGGACA ATGGTTTTCT CATGAAGAAG AAAaAGTATT ATTTGGAT.. .GATCGATaC AAACAAAAG. ..GAAaTTGA TTGCTATGTA ATGCATGATT TAATGCATGA GCTATCTAAG

2601 2730

Lr1_Victo_allele AGTGTTTCTG CACAAGAATG CCTCAATATA AGTGGCTTTG ATTTCAGAGC TGATGCCATC CCGCAATCTG TTCGACACTT ATCTATCAAC ATAGAAGACA GATATGATGC AAATTTTGAG GAAGAAATGT

TraesCS5D01G560500 AGTGTTTCTG CACAAGAATG CCTCAATATA AGTGGCCTAG ATTTCAGAGC TGATGTTGTC CCAAAATCTG TTCGACACTT ATCTATCAAC ATAGAAGACA GATATGATGC AAATTTTGAG CAAGAAATGT

Consensus AGTGTTTCTG CACAAGAATG CCTCAATATA AGTGGCcTaG ATTTCAGAGC TGATGccaTC CCaaAATCTG TTCGACACTT ATCTATCAAC ATAGAAGACA GATATGATGC AAATTTTGAG cAAGAAATGT

2731 2860

Lr1_Victo_allele CTAAACTAAG GGAGAAGATA GACATTGCTA ATGTGCGGAC TTTGATGATT TTTAGAGAAT ATGAAGAAGA AAGAACCGCC AAGATATTGA AAGATAGCTT CAAGGAAATA AATAGTCTGC GTGTCCTATT

TraesCS5D01G560500 GTAAACTGAG GGAGAGGATA GACATTGCTA ATCTGCGAAG TTTGATGACT TTTAGAGGAT ATGAAGAAGA AAGAATCAAC AAGCTTCTAA AAGATAGCTT CAAGGAAATA AATAGTCTGC GTGTCCTATT

Consensus cTAAACTaAG GGAGAaGATA GACATTGCTA ATcTGCGaAc TTTGATGAcT TTTAGAGaAT ATGAAGAAGA AAGAAcCaaC AAGaTacTaA AAGATAGCTT CAAGGAAATA AATAGTCTGC GTGTCCTATT

2861 2990

Lr1_Victo_allele TATAGTGGTG AAGTCTGCAC AATCTTTTCC GGATATGTTT TCAAAACTTA TCCACCTCCA GTACCTCAAA ATTAGTTCAC CTC---ACAT TGACGGGGAA ATGAGGTTAC CTAGTACACT ATCAAGATTT

TraesCS5D01G560500 TACAGTGGTG AAGTCTGCGC AATGTTTTCC ATATAGGTTT TCAAAACTGA TCCACCTCCA GCACCTCAAA ATTAGTTCAT CTTTCTACAG TGACGGGGAA ATGAGTTTGC CTAGTACACT ATCAAGATTT

Consensus TAcAGTGGTG AAGTCTGCaC AATcTTTTCC agATAgGTTT TCAAAACTgA TCCACCTCCA GcACCTCAAA ATTAGTTCAc CTc...ACAg TGACGGGGAA ATGAGgTTaC CTAGTACACT ATCAAGATTT

2991 3120

Lr1_Victo_allele TATCACTTGA AATTCCTGGA CCTAGATGAT TGGCGTGGTA GTTCTGATTT GCCTGAAGAC TTTAGCCACC TTGAGAATTT ACATGATTTC CGTGCTGAAA GTAAACTCCA CTCCAATATT CGCAATGTTG

TraesCS5D01G560500 TATCACTTGA AATTCTTGGA CCTAGATGAT TGGAATGGTC GTTCTGATTT ACCTGCAGAC TTTAGCCACC TTGAGAATTT ACATGATTTC CGTGCTGGAA GTCAACTTCA CTGCAATATT CGCGATGTCG

Consensus TATCACTTGA AATTCcTGGA CCTAGATGAT TGGaaTGGTa GTTCTGATTT aCCTGaAGAC TTTAGCCACC TTGAGAATTT ACATGATTTC CGTGCTGaAA GTaAACTcCA CTcCAATATT CGCaATGTcG

3121 3250

Lr1_Victo_allele GAAAGATGAA GCATCTACAG AGGCTAGAAG AATTCCATGT TAAGAAGGAG AGCATGGGAT TTGAACTGTC AGAACTTGGG CCATTGACAG AGCTTGAAGG AGGACTGACT GTACGTGGTC TTGAACACGT

TraesCS5D01G560500 GAGAGATGAA GCATCTGCAG GAGCTAAAAG AATTCCATGT CAGGAAGGAG AGCATGGGAT TTGAACTGTC AGAACTTGGG GCATTGTCAG AGCTTGAAGA AGAACTGACT GTACGTGGTC TTGAACACGT

Consensus GAaAGATGAA GCATCTaCAG aaGCTAaAAG AATTCCATGT cAaGAAGGAG AGCATGGGAT TTGAACTGTC AGAACTTGGG cCATTGaCAG AGCTTGAAGa AGaACTGACT GTACGTGGTC TTGAACACGT

3251 3380

Lr1_Victo_allele GGCAACCAAG GAGGAAGCTA CTGCAGCCAA ACTGATGTTG AAAAGGAATC TGAAGGAGTT GGAATTACTC TGG---GACA GAGATGGACC AACTACAGAT GCTGATATTC TTGATGCTCT TCAACCACAC

TraesCS5D01G560500 GGCAACCAAG GAGGAGGCTA CTGCAGCCAA ACTGATATTG AAAAGGAATC TGAAGAAATT GGAATTACTC TGGTCAGGCC GAGATGGCCC AACTACAGAT GCTGGTATTC TTGATGCTCT TCAACCACAC

Consensus GGCAACCAAG GAGGAaGCTA CTGCAGCCAA ACTGATaTTG AAAAGGAATC TGAAGaAaTT GGAATTACTC TGG...GaCa GAGATGGaCC AACTACAGAT GCTGaTATTC TTGATGCTCT TCAACCACAC

3381 3510

Lr1_Victo_allele TCTAATCTTA GAGTACTTGC AATTGTAAAT CATGGTGGTA CCGTTGGTCC TAGCTGGTTG TGTCTTGACA TCTGGTTAAC AAGTTTAGAG ACTCTCACTC TAGCAGGCGT ATGTTGGAGC ACTCTCCCGC

TraesCS5D01G560500 TCTAATCTTA GAGTACTTAC AATTGCAAAT CATGGTGGTA TGATTGGTCC TAGCTGGTTG TGTCTTGACA TCTGGTTGAC AAGTCTAGAG ACTCTCACTC TAGAAGGCGT ATCTTGGAGC AACCTCCCAC

Consensus TCTAATCTTA GAGTACTTaC AATTGcAAAT CATGGTGGTA ccaTTGGTCC TAGCTGGTTG TGTCTTGACA TCTGGTTaAC AAGTcTAGAG ACTCTCACTC TAGaAGGCGT ATcTTGGAGC AacCTCCCaC

3511 3640

Lr1_Victo_allele CTTTTGCGAA GCTACCAAAT CTCAAGGGAC TCAAACTGAT GAGAATTTCT GGAATGCATC AGTTTGGGTC TCTATGTGGT GGCACTCCAG GGAAATGTTT TATGCGCTTG AAGACAGTTG AGTTTTATGA

TraesCS5D01G560500 CCTTTGCGAA GCTACCAAAT CTCAAGGGCC TCTATTTGAA TAAAATTTCT GGAATGCATC AGTTTGGGCC TCTATGTGGT GGCGCTCCAG GGAAATGTTT TATGCGCTTG AAGGAAGTTG GGTTTTATGA

Consensus CcTTTGCGAA GCTACCAAAT CTCAAGGGaC TCaAacTGAa gAaAATTTCT GGAATGCATC AGTTTGGGcC TCTATGTGGT GGCaCTCCAG GGAAATGTTT TATGCGCTTG AAGaaAGTTG aGTTTTATGA

3641 3770

Lr1_Victo_allele GATGCCAGAA CTTGCTGAAT GGGTTGTGGA ATCTAATTGC CATTCCTTTC CAAGTCTTGA AGAAATCAGA TGCAGAAATT GTCCCAACCT CCGTGTGATG CCCTTCTCGG AGGTATCTTT CACCAATTTG

TraesCS5D01G560500 GATGCCAGAA CTTGCTGAAT GGGTTGTGGA ACCTAATTGC CATTCGTTTC CAAGTCTTGA AACAATCGAA TGCATCGATT GTCCCAACCT CAGTGTGATG CCCTTCTCTG AGGTGTCTTG CACCAATTTG

Consensus GATGCCAGAA CTTGCTGAAT GGGTTGTGGA AcCTAATTGC CATTCcTTTC CAAGTCTTGA AaaAATCaaA TGCAgaaATT GTCCCAACCT CaGTGTGATG CCCTTCTCgG AGGTaTCTTg CACCAATTTG

3771 3900

Lr1_Victo_allele CGCACACTTT TTGTTTCCAG GTGCCCCAAG ATGTCTCTGC CCTCCATGCC TCACACCTCC ACACTGACAG ATCTGAATGT TGGAATAGGT GATTCAGAAG GGTTGCATTA TGATGGAAAG AAATTGATTG

TraesCS5D01G560500 CGCAGACTTT TTGTTTCTGG GTGCCCCAAG ATGTCTCTGC CGTCCATGCC TCACACATCC ACACTGACAA GTTGTGATGT AGGAAGATGT GATTCCGAAA GGTTGCGTTA TGATGGAAAG AGATTGGCTG

Consensus CGCAcACTTT TTGTTTCcaG GTGCCCCAAG ATGTCTCTGC CcTCCATGCC TCACACaTCC ACACTGACAa aTcggaATGT aGGAAgAgGT GATTCaGAAa GGTTGCaTTA TGATGGAAAG AaATTGacTG

3901 4030

Lr1_Victo_allele TTATAGGGTA TGGCGGTGCT TTGGCCTCCC ACAATCTGGA TACAGTAGAA GATATGATTG TCGAAAGATG CGACGGTTTG TTCCCTGAAG ATTTGGATGG CAGTTTTGTC TTCCGTTCAG TTAAGAATCT

TraesCS5D01G560500 TTAGAGAGTA TGGCGGTGCG TTGGCCTCCC ACAATCTGGA TAAAGTAGAA GATATGATTG TCAACAGATG CGACGGTTTG TTCCCTGAAG ATTTGGATGG CGGTTTTGTC TTCCGTTCAG TTAAGAATCT

Consensus TTAgAGaGTA TGGCGGTGCg TTGGCCTCCC ACAATCTGGA TAaAGTAGAA GATATGATTG TCaAaAGATG CGACGGTTTG TTCCCTGAAG ATTTGGATGG CaGTTTTGTC TTCCGTTCAG TTAAGAATCT

4031 4160

Lr1_Victo_allele CACATTACAT GTATCTCGTC TTACCAGCAG CAAATCATCA TCGTGAAAAG TGTTAAACTG TTTCCCAGCT CTTTCTGTGT TGGTGATAGT TGGCTATGAG GA---ATGTG TAATGCAGTT CCCATCATCC

TraesCS5D01G560500 CACATTACAT GTATCTCATC TTACCAGCAG CAAATCATCA TCGTCAAAAG TGTTAAACTG TTTCCCAGCT CTTTCTGTGT TGCACATAGA TCACTGTGAG GAGGAATGTG TAATGCAGTT CCCATCATCC

Consensus CACATTACAT GTATCTCaTC TTACCAGCAG CAAATCATCA TCGTcAAAAG TGTTAAACTG TTTCCCAGCT CTTTCTGTGT TGcacATAGa TcaCTaTGAG GA...ATGTG TAATGCAGTT CCCATCATCC

4161 4290

Lr1_Victo_allele AGCTCACTGC AGAAACTTAC CTTCTCAGGG TGTAAGGGCC TAGTTCTTGT GCCTGTGGAG AA------TG GAGGAGGAAT TCAGGAGGAC AAGTCATTGC TCCAATCATT AACCATAGTC AGCTGTGGCG

TraesCS5D01G560500 AGCTCACTGC AGAAAGTTAA CTTCTCATGC TGCAAGGGCC TGGTTCTTGT GCCTGTGGAG AAGGAGAATG GAGGAGGAAT TCGGGAGGAC AACTCATTGC TCCAATCATT AACAATATTC AATTGTGGTC

Consensus AGCTCACTGC AGAAAcTTAa CTTCTCAgGc TGcAAGGGCC TaGTTCTTGT GCCTGTGGAG AA......TG GAGGAGGAAT TCaGGAGGAC AAcTCATTGC TCCAATCATT AACaATAgTC AacTGTGGcc

4291 4420

Lr1_Victo_allele AATTGTTCTG TCGGTGGCCA ATGAGA---- --GAATCAGA GACCATTTGC CCTTTCCCTG CTTCCCTGAG GGAACTTGAT GTTTTCCAAG AGCCAAGCAT GAAGTCAATG GCTCTGCTCT CAAACCTCAC

TraesCS5D01G560500 GATTTTTCTG TGGGTGGCCC ATGGGAAAGG GAGAATCAGA GACCATTTGC CCTTTCCCTG CTTCCCTGAG GGAACTTGAT GTCGAAGGAG AGGCAAGCAT GAAGTCAATG GCTCTGCTCT CAAACCTCAC

Consensus aATTgTTCTG TcGGTGGCCa ATGaGA.... ..GAATCAGA GACCATTTGC CCTTTCCCTG CTTCCCTGAG GGAACTTGAT GTcgaacaAG AGcCAAGCAT GAAGTCAATG GCTCTGCTCT CAAACCTCAC

4421 4550

Lr1_Victo_allele GTCTCTCACC ACTCTACAGC TAAACTACTG CAGTAATTTA ACAGTGGATG GATTCAATCC TCTCATCGCA GTCAACCTCA TAGAGCTGCA AGTGCATAGG TGCAACACCT TAGCAGCAGA TATGCTCTCA

TraesCS5D01G560500 GTCTCTCACC ACTCTAAAGC TAGAGGAGTG CGGTAATTTA ACAGTGGATG GATTCAATCC TCTCATCGCA GTCAACCTCA GAGAACTGCA AGTGTGTGGG TGCAACACCT TAGCAGCAGA TATGCTCTCA

Consensus GTCTCTCACC ACTCTAaAGC TAaAcgAcTG CaGTAATTTA ACAGTGGATG GATTCAATCC TCTCATCGCA GTCAACCTCA gAGAaCTGCA AGTGcaTaGG TGCAACACCT TAGCAGCAGA TATGCTCTCA

4551 4680

Lr1_Victo_allele GAGGCGGCCT CTCACTCTCA GAGGGCCAAA TTATT---GC CTGCAGGTTA CATCTCTAGA TTGGAGGTAC TCATCGTGGA TAACATCTGT GGATTGCTTG TTGCTCCTAT TTGCATCCTC CTCGCCCCGG

TraesCS5D01G560500 GAGGTAGCCT TTCA------ GAGGGCCAAA TTATTATTGC CTGCAGGTTA CATCTCTAGA TTGGAGGTAC TCATCACGGA CGACATCTCT GGATTGCTTG TTGCTCCTAT TTGCAATCTC CTCGCCCCAG

Consensus GAGGcaGCCT cTCA...... GAGGGCCAAA TTATT...GC CTGCAGGTTA CATCTCTAGA TTGGAGGTAC TCATCacGGA caACATCTcT GGATTGCTTG TTGCTCCTAT TTGCAacCTC CTCGCCCCaG

4681 4810

Lr1_Victo_allele CCCTCCACAC ACTTGTATT- ----CTGGAT T--------- ---------- ---GATGAAA CGATGGAAAG CTTGACGGAA GAGCAGGAGA AAGCGCTGCA GCTCCTCACC TCCCTCCAGA ATCTAACATT

TraesCS5D01G560500 CCCTCCACAC ACTTGTATTT GGTTCTGATT TTTTTCGAAA AGGATTTGGT TCTGATGGGA GGATGGAATG CTTCACGGAA GAGCAAGAGA AAGCGCTGCA GCTCCTCACC TCCCTCCAGA AACTAAGTTT

Consensus CCCTCCACAC ACTTGTATT. ....CTGaaT T......... .......... ...GATGaaA cGATGGAAaG CTTcACGGAA GAGCAaGAGA AAGCGCTGCA GCTCCTCACC TCCCTCCAGA AaCTAAcaTT

4811 4940

Lr1_Victo_allele TTTCAGATGC AGGGGTCTAC AGTCCCTTCC TCAAGGGTTG CATCGCCTTT CTTCTCTCAA GGAGTTATGT GTCCGTGGGT GTCTAAAAAT CCAATCGTTG CCCAAGGAGG GCCTCCCGCT TTCGCTGAGA

TraesCS5D01G560500 TTACTCCCGC AAGGGTCTGC AGTCCCTTCC TCAAGGGTTA CATCGCCTTT CTTCTCTCAA GGAGTTATGT GTCAGGTACT GTCCAAATAT CCGATCCATG CCCAAGGAGG GCCTCCCAGT TTCGCTGAGA

Consensus TTaCacacGC AaGGGTCTaC AGTCCCTTCC TCAAGGGTTa CATCGCCTTT CTTCTCTCAA GGAGTTATGT GTCaGggacT GTCcAAAaAT CCaATCcaTG CCCAAGGAGG GCCTCCCacT TTCGCTGAGA

4941 5070

Lr1_Victo_allele AGACTAAAGA TGAATTGGCG CAGCGCTGAG ATAAACGAGC AAATTGAGAA AATCAAAAGA AGCAACCCAG ATTTATCCGT CTCGTATTGC TAACTACACC CAAGGTAACA CTTGTCGCCT CCCTATTTTG

TraesCS5D01G560500 AAACTATATA TGACTGGTCG CAGCGCTGAG ATAGAGGAGC AAATTGAGAA AATCAAAAGA ACCAACCCAG ATTTATCCGT CG---AAACA TAACTACACC CAAGGTAACA CTTGTCGCCT CCCTATTTTG

Consensus AaACTAaAgA TGAaTgGgCG CAGCGCTGAG ATAaAcGAGC AAATTGAGAA AATCAAAAGA AcCAACCCAG ATTTATCCGT Cg...Aaaca TAACTACACC CAAGGTAACA CTTGTCGCCT CCCTATTTTG

5071 5200

Lr1_Victo_allele ATTGTTTCTC TATTTCTGAT GAAACGAGGT TTATTCGCCA TCCATATATA TTTCTGCCTC ATCTTAACCA CTGTTGTCAA ATCTTACAGA CTA---GCAG GTTC---CAC TGTTG-CAAT --CAATCTTT

TraesCS5D01G560500 ATTGTTTCTC TATTTCTGAT GAAACGAGGT TTATTCGCCA TCCATGTATA TTTCTGCCTC ATCTTAACCA CTGTTGTCAA ATCATACAGA CTACTAACGG TTGCTCTCCC TGTTGGCGGT TGCCACCTTT

Consensus ATTGTTTCTC TATTTCTGAT GAAACGAGGT TTATTCGCCA TCCATaTATA TTTCTGCCTC ATCTTAACCA CTGTTGTCAA ATCaTACAGA CTA...aCaG gTgC...CaC TGTTG.CaaT ..CaAcCTTT

5201 5330

Lr1_Victo_allele T---GTGCAC A------CAG GTTGTGAAAC ----CTGCAC ATTAATCA-G CAGGTCAGGT GCGAGATACA ACCTACCGAC TCTTCATTCT ------GCAA CGCAACACTA GCTGTGAACT CTTCATTCTG

TraesCS5D01G560500 TCACGGCCTC AATCATGCTG GTGAAGACAC TGATCTCCTC CTCCATCAAG CCGCCCCTCT TCCTCTTCCC ATCAAGCACA TGTACGTGTT CAAAGGGCTT GTCAGGGCCA TCAAGGACTA CCTCCGCGTC

Consensus T...GgcCaC A......CaG GTgaaGAaAC ....CTcCaC aTcaATCA.G CaGccCagcT gCcacaTaCa AcCaAcCaaa TcTaCaTgcT ......GCaa cgCAacaCcA gCaagGAaca CcTCagccTc

5331 5460

Lr1_Victo_allele CTG-----CA TGATCT---G CCGGTTAGTA CCT-----CT CCAC---CTC CAGAACCAAA TT-CCTTTTT TTAGA--AAA GGAGGATATG ACCCCCGGC- ---------- CTC----TGC ATC------T

TraesCS5D01G560500 CGGGGTCTCT GGAACTCGGG CATGAGCGAA CCGAGAGGCT CATCGGAATC CATGGCATGC TTGCCAGTTG CCAGCCCAAA TGAGAAGATG TGCTACATCT GGTTGTAGTT CGCAGATTGC AGCCAAGTGT

Consensus CgG.....Ca gGAaCT...G CagGagaGaA CCg.....CT CaaC...aTC CAgaaCaaaa TT.CCagTTg ccAGa..AAA gGAGaAgATG acCcaCagC. .......... CgC....TGC AgC......T

5461 5590

Lr1_Victo_allele GGGCGAT--- -GCATACGGC CA---CTCCA GAACCA--AA TTCCTGATCC TTCATTTATG ACTGCATTCA TTTCATTTAG -GCCTCCTGA GTAGCTGCGT TATTGTTATA GGAGTTGCCT ATTATTCCA-

TraesCS5D01G560500 GGGGGTTTAC CACTTGTGGC AAATGCTCTT GGGCAAGTAA TGTCCGAGCT AAGGACCGTG GGGGCATGGG AACATATAAG AGACAGCAAG GTTGATTTGG GTTTGAGAGA AGGACATCAG AAAGAAACAT

Consensus GGGcGaT... .aCaTacGGC aA...CTCca GaaCaA..AA TgcCcGAgCc aacaaccaTG acgGCATgca aacaaaTaAG .GaCacCaaa GTaGaTgcGg gaTTGagAgA aGaacagCag AaaaaaaCA.

5591 5720

Lr1_Victo_allele -------GCT --TGTTCTTC ATTGTCT--- -GACCCAAGA ATA----GTT GGTTACCCGA ATT

TraesCS5D01G560500 TAGAGAGGCT AATGCTCAGC TATTACTACA TGAAGCTAGA ATACAAAATG TGTTTCACGT ACTTGGCAAC CTTCTCCAAG GGCTTTGTCT TGGACAGCAA TCGTCTAATC CAGCAATGGA ATGCACTTGG

Consensus .......GCT ..TGcTCagC aaTgaCT... .GAacCaAGA ATA....aTg gGTTaCaCGa AcT....... .......... .......... .......... .......... .......... ..........

5721 5850

Lr1_Victo_allele

TraesCS5D01G560500 ATACATTAAT TCAAGGCATG ATGGTCAAAG GTGCATCAAC TACCTTTTGG GGATGTCCTT TCTTCAGATT CCAGGATCTA CTTCGGTTAG TACCTATAGG CACCATTTCT GGCTGGTTCA TTTTTACCAT

Consensus .......... .......... .......... .......... .......... .......... .......... .......... .......... .......... .......... .......... ..........

5851 5980

Lr1_Victo_allele

TraesCS5D01G560500 CTTTTCTTTT TCCTTTATTT ATAGACAAGG ATGCGCTTTC ATAAATTATA CTCTGTTATG ATTCTAGATT ATACAAATTT TTGAGAATCC ATCGTTTCTA GTAAAAAAAT GTATATGAAA ATTGTTCTGT

Consensus .......... .......... .......... .......... .......... .......... .......... .......... .......... .......... .......... .......... ..........

5981 6110

Lr1_Victo_allele

TraesCS5D01G560500 ATAAGGGCTC CTCAGATGTT GAGCAAGTTG CGATTTTACT AGACCAGTTC AGTGTCTTCT CTCAAGTCCA TTTTAACATT TCCTATTCTT TAGGGATTTT ACATTTCTCT TTGCAATACT ATATTGATAG

Consensus .......... .......... .......... .......... .......... .......... .......... .......... .......... .......... .......... .......... ..........

6111 6240

Lr1_Victo_allele

TraesCS5D01G560500 TAGTTATCTC TGCAGGTTAG TCCAAGTCCA TTGCATTTCA AAGCTCCTCC ACAACTCGTC ATGCATGATT TGGTGCATGA TCTCGCATCA ATAATTGTTG CTGATGAATT CATTGATCTG GATGCTACCA

Consensus .......... .......... .......... .......... .......... .......... .......... .......... .......... .......... .......... .......... ..........

6241 6370

Lr1_Victo_allele

TraesCS5D01G560500 AAAGCACCCA GAGGTTAGAT CTGATGAAGA GAGCCCGTTA CTGCCGACAT GCACAGTTAA CCAACTTCAA GAATGATCCA GAGATTTTCA AATATATTCC ACACAAGCTT AGATCCCTCC ACTTTAGGGA

Consensus .......... .......... .......... .......... .......... .......... .......... .......... .......... .......... .......... .......... ..........

6371 6500

Lr1_Victo_allele

TraesCS5D01G560500 TTTGGTGGGA CTGCAACTCC CAAAAAAGGC ATTTTCTCGG TCCAAGTACA TACGTGTCCT GGACCTAAGT GGACATTCAG CTAATGGCCA ATCTGCTCCA AGTAGCGTGG CACTGCCATC TTCTGTTAAG

Consensus .......... .......... .......... .......... .......... .......... .......... .......... .......... .......... .......... .......... ..........

6501 6630

Lr1_Victo_allele

TraesCS5D01G560500 AAATTGAAGC TGATTAGGTA CCTTGATGCC ACAGGCTTGC CAATAACATC ACTTCCTAAG TATTTTCATG AACTTCAAAA CATGGAAACT CTTATCCTGT CCAATTGCTT GCTTGAAACC TTGCCTGACA

Consensus .......... .......... .......... .......... .......... .......... .......... .......... .......... .......... .......... .......... ..........

6631 6760

Lr1_Victo_allele

TraesCS5D01G560500 ATATTTGTCG CCTCAGCAAA CTTTGCTATT TGGACCTATC TGGCAGTAGC AGCCTCAGTA AGCTAAATGC ATCACTAGGG GAGCTCTCTC AACTCTTCTT CCTCAATCTA TCCGGGTGTT ATATACTCCA

Consensus .......... .......... .......... .......... .......... .......... .......... .......... .......... .......... .......... .......... ..........

6761 6890

Lr1_Victo_allele

TraesCS5D01G560500 AGAGTTGCCT GAATCAATCT GTGAGCTTAG ATGCTTACAC CACCTAGACA TGTCAAATTG TCATAACCTC GAAGAGCTCC CTGAATGTTT TGATCAACTC TTCGAGCTTG AATATTTGAA TCTAAAAAGC

Consensus .......... .......... .......... .......... .......... .......... .......... .......... .......... .......... .......... .......... ..........

6891 7020

Lr1_Victo_allele

TraesCS5D01G560500 TGTCCTAAAC TCCGACAATT ACCAGAGTCA CTATGCAAGT TGTTCAAGCT AAGGTATCTC TATTTGTCAT ACTGTCTGAG TCTCAATGAG CTCCCCTCCT CATTTGGTGA CCTTAAGCTT CAAATACTGC

Consensus .......... .......... .......... .......... .......... .......... .......... .......... .......... .......... .......... .......... ..........

7021 7150

Lr1_Victo_allele

TraesCS5D01G560500 ACATGAATGG TCTTGTACTT ATGAAGGACT GCTCTGATAG CATTGGTGAC ATGACTAGTC TCACCGAGCT GGTGATTGAT AATGCAACTT ATGATTTGCC TGAAAAGGCT CGAGCCATTG AAAAACGTCT

Consensus .......... .......... .......... .......... .......... .......... .......... .......... .......... .......... .......... .......... ..........

7151 7280

Lr1_Victo_allele

TraesCS5D01G560500 AAATCTTGTG GGCACAGTAG AGCACCGTGT ACACGAGATA GAGAGTAGAG GATGCAGCAG TATAGTGGAT CTTGTGGGAT TGACTTGTTC ACAGCTGATG CTTCTAGACC TTCAGAATGT CAGGCAGTCA

Consensus .......... .......... .......... .......... .......... .......... .......... .......... .......... .......... .......... .......... ..........

7281 7410

Lr1_Victo_allele

TraesCS5D01G560500 GAAGATGCAG ACAGAGTCAA ACTGCGTGAT AAATCAGATA TTCAAGTACT AAAACTTCAC TTCGGAAATA AAGGAGGTGA ATCTGTACTG GGCAGGCTCG TTCCTCCTCG GACTCTTGAA GACTTTTCGC

Consensus .......... .......... .......... .......... .......... .......... .......... .......... .......... .......... .......... .......... ..........

7411 7540

Lr1_Victo_allele

TraesCS5D01G560500 TAATTGGGTA TAGGAGCAAT GAATTCCCTG ACTGGATGTT TGACATCTCG TCCTACCTGC CTTTTCTCAG TGAACTGACT CTTGATGGTT TGGAAGCATG TGATTGTCTT CCTCCATTTG GGGCACTACC

Consensus .......... .......... .......... .......... .......... .......... .......... .......... .......... .......... .......... .......... ..........

7541 7670

Lr1_Victo_allele

TraesCS5D01G560500 AAATTTAAGA AGGCTGTGTT TGATAAACAT TCCCAACATT AGGAAAATTG GTAAGGAGTT CTATGGAGAG GGTGGACCTT GTATGAAACT AAGAATACTG CTATTGACGT TGATGGAAAA TTTGGTGGAA

Consensus .......... .......... .......... .......... .......... .......... .......... .......... .......... .......... .......... .......... ..........

7671 7800

Lr1_Victo_allele

TraesCS5D01G560500 TGGTGGACAA CAGAGTCAAG TGAAGAAACA AAAGGGTTTC TGATCCCTAA TTTGCATCAT TTGGAGATAA AGGACTGCCC AAGGTTGAAG TTCCTACCAT ATCCCCCAAG AAGTATGAAT TGGGTTTTGA

Consensus .......... .......... .......... .......... .......... .......... .......... .......... .......... .......... .......... .......... ..........

7801 7930

Lr1_Victo_allele

TraesCS5D01G560500 GCAACAGCGA GACAGTTTTG CCAGAACAAG GATTTGGAAA GCTCTCGTCT TCCATCCGTC CTTCTTTGAT GGTTCTAAAG AGGTGTAGTT TCTCTCAAGA CAAGTGGGAT AGACTTCAAC ACTTTCCCAC

Consensus .......... .......... .......... .......... .......... .......... .......... .......... .......... .......... .......... .......... ..........

7931 8060

Lr1_Victo_allele

TraesCS5D01G560500 CCTTGAGAAA TTTCAGGTAA TCTCGGCCAG TGGATTGAGG ACTTTGCCAG TGGTCATGCG AGGCTTCACC TCTCTCACTG AACTATATTT GAAGTCATTG AAGTTCTTGG AGGCACTCCC GGTATGGTTC

Consensus .......... .......... .......... .......... .......... .......... .......... .......... .......... .......... .......... .......... ..........

8061 8190

Lr1_Victo_allele

TraesCS5D01G560500 GGTGACCTCG CTTCTCTAGT TGAAATTTCA ATCGATGATT GCCCCAACCT GACATGTTTG CCTGAAAGCA TGAAGAACCT CACTGCTCTA AGAAAATTGA CGTTGATAGA GTGCAAAGGC ATGGAAACAT

Consensus .......... .......... .......... .......... .......... .......... .......... .......... .......... .......... .......... .......... ..........

8191 8320

Lr1_Victo_allele

TraesCS5D01G560500 TACCGGCACC GTTGGGACAT TCAACTTCTC TAGAAGAAAT TTTAATCGGC GATTGCCACA ACCTGACATC TTTGCCTGAA AGCATGAAGA ACCTCTCCGC TCTTAAAAAC CTGACGTTGA TAAAGTGCAA

Consensus .......... .......... .......... .......... .......... .......... .......... .......... .......... .......... .......... .......... ..........

8321 8450

Lr1_Victo_allele

TraesCS5D01G560500 ATGCCTGGAA ACATTACCGG GATTGTTGGG ACAGTTGACT TCTTTAGAAG AAATCTTAAT CGGCGATTGC CCCAACCTGA CTTCTTTGCC TGAAAGCATT AAGAACCTCA TCGCTCTTAA AGTACTGAGG

Consensus .......... .......... .......... .......... .......... .......... .......... .......... .......... .......... .......... .......... ..........

8451 8481

Lr1_Victo_allele

TraesCS5D01G560500 TTGACAGAGT GCAAAGGCCT GGAAACATTG C

Consensus .......... .......... .......... .

b)

1 130

Lr1_Victo_allele GCCCACACGC CCAAGCGTGA TGGCATCTTC CATATGTTTT ATCGGATGTT TGTTTATCTA CAAAACCTTC CACTCTCCCT AAAAAACCAA GGACTTGGAT GGTTCCGAAA TTTCCATATA AATGCAACCC

TraesCS5D01G561200

Consensus .......... .......... .......... .......... .......... .......... .......... .......... .......... .......... .......... .......... ..........

131 260

Lr1_Victo_allele GTGAAAATGT TGAGATATGT TCTTACACAT GCTCACCGCC AGTGGCAGAT CCGGGACCCA TGCCGGTGGG GCCTAGGCCT AGGGCATGAG AATGATTTAC TTCGTTGACC GCAGCTTATT GAGCACTGTA

TraesCS5D01G561200

Consensus .......... .......... .......... .......... .......... .......... .......... .......... .......... .......... .......... .......... ..........

261 390

Lr1_Victo_allele GCTGCACTGT AGCGCCTGGG GCCTGGGCTT GGCCCAATCC TGGGTCCGCC CCTGCTCACC GCCTAAAGGA GATACAAAAG AAATATTCTA AATGGCAGAA AAATCTTGGG ATTTTGAAAT CTAAATCATG

TraesCS5D01G561200

Consensus .......... .......... .......... .......... .......... .......... .......... .......... .......... .......... .......... .......... ..........

391 520

Lr1_Victo_allele TACTCACTCC GTCCCAAAAT AAGTGTTTCA ACTTTAGTAT AGTTTTGTAC TAAAGCTAAT ACAGAGTTGA GACACTTATT TTGAGACGGG AGGATATGTA TTTTCTGTTA TGTTTCGGAT TGTATCTCGA

TraesCS5D01G561200

Consensus .......... .......... .......... .......... .......... .......... .......... .......... .......... .......... .......... .......... ..........

521 650

Lr1_Victo_allele GAAGAATGTT CTTATATTAT GGAACGGGGA GTCTTATTGT TTGATTAATT ATTAATAAAG CCGACATTCT GTAAAGAAAC CTTTCAGCAG GGGTGTATTC GTAAAAGTTT TGCATTACCG GCTTGGGTCC

TraesCS5D01G561200

Consensus .......... .......... .......... .......... .......... .......... .......... .......... .......... .......... .......... .......... ..........

651 780

Lr1_Victo_allele CAATAATTTG TATCCATAGG TTGGTCAAAC AGCTCAAACC ATACCAACCA AGCAAGCAAG CAATCCCCAC ACCGCTAGAG CTACAGCTTC CACCGGCGTC AAAAGAAAAC ACCAATTATT GCCCCGGCGA

TraesCS5D01G561200

Consensus .......... .......... .......... .......... .......... .......... .......... .......... .......... .......... .......... .......... ..........

781 910

Lr1_Victo_allele CTCGACGTCC GGCGGCCGGC GGAGTGCAGA TCTACGGCTG TGCGGTGGCG TGGGCAGTTG GGGAAGAGTA CCCTGTCCTT GCTCCCCACA CGACACCGCT CCCCGGCGGC GTTGACCGAG CTCGGAGCTG

TraesCS5D01G561200

Consensus .......... .......... .......... .......... .......... .......... .......... .......... .......... .......... .......... .......... ..........

911 1040

Lr1_Victo_allele AGGCGAGGCG GCCGGCGGAG TGCAGATGGC GGCGGCTCTC GGCTCGGCGG CCACGCTCCT CGGCAAGGTG TTCACTATGC TGTCCGCCGC CCCGGTGGCG GCGTACGTGG ACAGCCTGGA GCTCGGCCAC

TraesCS5D01G561200 ATGGC GGCGGCTCTC GGCTCGGCGG CCACGCTCCT CGGCAAGGTG TTCACTATGC TGTCCGCCGC CCCGGTGGCG GCGTACGTGG ACAGCCTGGA GCTCGGCCAC

Consensus .......... .......... .....ATGGC GGCGGCTCTC GGCTCGGCGG CCACGCTCCT CGGCAAGGTG TTCACTATGC TGTCCGCCGC CCCGGTGGCG GCGTACGTGG ACAGCCTGGA GCTCGGCCAC

1041 1170

Lr1_Victo_allele AACTCCCAGC AGATCAGGGC CAAGCTCGCG CACACGCGAG GCCTGCTGCA CAACGCCCAA GCCCAGGTGA GCGACGTCGG CCACAACCCT GGACTGCAGG AGCTGCTGCC GGCGCTGAGC AGGAACGCCG

TraesCS5D01G561200 AACTCCCAGC AGATCAGGGC CAAGCTCGCG CACACGCGAG GCCTGCTGCA CAACGCCCAA GCCCAGGTGA GCGACGTCGG CCACAACCCT GGACTGCAGG AGCTGCTGCC GGCGCTGAGC AGGAACGCCG

Consensus AACTCCCAGC AGATCAGGGC CAAGCTCGCG CACACGCGAG GCCTGCTGCA CAACGCCCAA GCCCAGGTGA GCGACGTCGG CCACAACCCT GGACTGCAGG AGCTGCTGCC GGCGCTGAGC AGGAACGCCG

1171 1300

Lr1_Victo_allele ACGAGGCGGA GGATCTGCTG GATGAGCTCC ACTACTTCCA GATCCATGAC AGGCTCCACG CCACCAACTA CGCCGCCACC CAGGCCAATT TTCTCCGTCA TGCTCGCAAT GCTCTCCGCC ACACTGCCAC

TraesCS5D01G561200 ACGAGGCGGA GGATCTGCTG GATGAGCTCC ACTACTTCCA GATCCATGAC AGGCTCCACG CCACCAACTA CGCCGCCACC CAGGCCAATT TTCTCCGTCA TGCTCGCAAT GCTCTCCGCC ACACTGCCAC

Consensus ACGAGGCGGA GGATCTGCTG GATGAGCTCC ACTACTTCCA GATCCATGAC AGGCTCCACG CCACCAACTA CGCCGCCACC CAGGCCAATT TTCTCCGTCA TGCTCGCAAT GCTCTCCGCC ACACTGCCAC

1301 1430

Lr1_Victo_allele CAGCAGCTGG GCCGCATGCT TTTCTTGTTC CTCTGCACAA GATGATAGTG ATTCTACCAG TGGGGATGAT GAGTTACGTT TCCACCGAGT GATCTTCTCC AGAAAATTCA AGTCGGTGTT ACAGGACATG

TraesCS5D01G561200 CAGCAGCTGG GCCGCATGCT TTTCTTGTTC CTCTGCACAA GATGATAGTG ATTCTACCAG TGGGGATGAT GAGTTACGTT TCCACCGAGT GATCTTCTCC AGAAAATTCA AGTCGGTGTT ACAGGACATG

Consensus CAGCAGCTGG GCCGCATGCT TTTCTTGTTC CTCTGCACAA GATGATAGTG ATTCTACCAG TGGGGATGAT GAGTTACGTT TCCACCGAGT GATCTTCTCC AGAAAATTCA AGTCGGTGTT ACAGGACATG

1431 1560

Lr1_Victo_allele CAGACGCACT GTGATTCCGT CTCTGATTTG CTCGGCACTA TCCCAACCAG CAGCATGCCA GTTGCTGTAC ACCGGCCGCA GATTGGATCC ACAATTATAC AAGATACATT GTATGGCAGG AGACACACTT

TraesCS5D01G561200 CAGACGCACT GTGATTCCGT CTCTGATTTG CTTGGCAATA TCCCAAGCAA CAGCATGCCA GTTGCTGTAC ACCGGCCGCA GATTGGATCC ACAATTATAC AAGATACATT GTATGGCAGG AGACACACTT

Consensus CAGACGCACT GTGATTCCGT CTCTGATTTG CTcGGCAaTA TCCCAAcCAa CAGCATGCCA GTTGCTGTAC ACCGGCCGCA GATTGGATCC ACAATTATAC AAGATACATT GTATGGCAGG AGACACACTT

1561 1690

Lr1_Victo_allele TTGAGGAAAC TGTCAACCGT ATCTTCAGCT GCAAACACCC TGTTTCTGTT CTTCCTATAG TTGGTCCAGG GGGTATTGGA AAGACAACTT TTGCTCAACA TCTGTATAAT GATGCAAGGA CTGAAGAGCA

TraesCS5D01G561200 TTGAGGAAAC TGTCAACCGT ATCATCAGCT GCAAACACCC TGTTTCTGTT CTTCCTATAG TTGGTCCAGG GGGTATTGGA AAGACAACTT TTGCTCAACA TCTGTATAAT GATGCAAGGA CTGAAGAGCA

Consensus TTGAGGAAAC TGTCAACCGT ATCaTCAGCT GCAAACACCC TGTTTCTGTT CTTCCTATAG TTGGTCCAGG GGGTATTGGA AAGACAACTT TTGCTCAACA TCTGTATAAT GATGCAAGGA CTGAAGAGCA

1691 1820

Lr1_Victo_allele CTTCCAAGTC AGGGTCTGGG TGTGTGTATC CACTGATTTC AATGTGCTTA AGCTCACCAG GGAGATCCTT GCCTGCATAC CTGCAACTGA AGAAGGAGGA AGCAGCAGTG TTGCAAATGA AACAACCAAT

TraesCS5D01G561200 CTTCCAAGTC AGGGTCTGGG TGTGTGTATC CACTGATTTC AATGTGCTTA AGCTCACCAG GGAGATCCTT GCCTGCATAC CTGCAACTGA AGAAGGAGGA AGCAGCAGTG TTGCAAATGA AACAACCAAT

Consensus CTTCCAAGTC AGGGTCTGGG TGTGTGTATC CACTGATTTC AATGTGCTTA AGCTCACCAG GGAGATCCTT GCCTGCATAC CTGCAACTGA AGAAGGAGGA AGCAGCAGTG TTGCAAATGA AACAACCAAT

1821 1950

Lr1_Victo_allele TTAGATCATC TTCAGAGATC CATTGTGCGC CGTCTCAAGT CCAAGAGGTT TCTAATTGTC TTGGACGATA TATGGAAATG TGACAGTCAG GATCAGTGGA AAACCTTGTT AGCTCCCTTC ACAAAGGGGG

TraesCS5D01G561200 TTAGATCATC TTCAGAGATC CATTGTGCGC CGTCTCAAGT CCAAGAGGTT TCTAATTGTC TTGGACGATA TATGGAAATG TGACAGTCAG GATCAGTGGA AAACCTTGTT AGCTCCCTTC ACAAAGGGGG

Consensus TTAGATCATC TTCAGAGATC CATTGTGCGC CGTCTCAAGT CCAAGAGGTT TCTAATTGTC TTGGACGATA TATGGAAATG TGACAGTCAG GATCAGTGGA AAACCTTGTT AGCTCCCTTC ACAAAGGGGG

1951 2080

Lr1_Victo_allele AAACCAAAGG AAGCATGCTA CTTGTCACAA CTCGATTCCC AAAGCTAGCA CAAATGATGG AAACAATTGA TCCACTAGAG CTGCTAGGTT TGGAGTCTAA TGACTTCTTC ACATTCTTTG AAGCATGTAT

TraesCS5D01G561200 AAACCAAAGG AAGCATGCTA CTTGTCACAA CTCGATTCCC AAAGCTAGCA CAAATGATGG AAACAATTGA TCCACTAGAG CTGCTAGGTT TGGAGTCTAA TGACTTCTTC ACATTCTTTG AAGCATGTAT

Consensus AAACCAAAGG AAGCATGCTA CTTGTCACAA CTCGATTCCC AAAGCTAGCA CAAATGATGG AAACAATTGA TCCACTAGAG CTGCTAGGTT TGGAGTCTAA TGACTTCTTC ACATTCTTTG AAGCATGTAT

2081 2210

Lr1_Victo_allele ATTTGGTGAA GACAACAAGC CAGAGCATTT CGAAGATGAG TTAGCTGGTA TTGCACAAAA AATTGCAGAC AAGCTAAAGG GTTCCCCGCT GGCAGCCAAA ACAGTTGGTA GACTATTGCA CAAGGACCTT

TraesCS5D01G561200 ATTTGGTGAA GACAACAAGC CAGAGCATTT CGAAGATGAG TTAGCTGGTA TTGCACAAAA AATTGCAGAC AAGCTAAAGG GTTCCCCGCT GGCAGCCAAA ACAGTTGGTA GACTATTGCA CAAGGACCTT

Consensus ATTTGGTGAA GACAACAAGC CAGAGCATTT CGAAGATGAG TTAGCTGGTA TTGCACAAAA AATTGCAGAC AAGCTAAAGG GTTCCCCGCT GGCAGCCAAA ACAGTTGGTA GACTATTGCA CAAGGACCTT

2211 2340

Lr1_Victo_allele TCTCAGAAAC ATTGGAATGG AGTTCTTGAA AAGCATCAGT GGCTAAAGCA GCAAAATAAT GATGATATCA TGCCATCTTT AAAAATTAGC TATGATTGCC TCCCTTTTGA TCTGAAGAAA TGTTTTTCCT

TraesCS5D01G561200 TCTCAGAAAC ATTGGAATGG AGTTCTTGAA AAGGGTGAGT GGCTAAAGCA GCAAAATAAG GATGATATCA TGCCATCTTT AAAGATTAGC TATGATTGCC TCCCTTTTGA TCTGAAGAAA TGCTTTTCCT

Consensus TCTCAGAAAC ATTGGAATGG AGTTCTTGAA AAGcaTcAGT GGCTAAAGCA GCAAAATAAg GATGATATCA TGCCATCTTT AAAaATTAGC TATGATTGCC TCCCTTTTGA TCTGAAGAAA TGcTTTTCCT

2341 2470

Lr1_Victo_allele ATTGTGGCCT TTTCCCCGAA GATCATTGGT TTACTTCTTC AGAAATCAAT CATTTCTGGG TTGCAGTAGG CATCATAGAC TCTGATCACC AAGCCGATAG GAATTACCTG GAAGAGCTAG TGGACAATGG

TraesCS5D01G561200 ATTGTGGCCT TTTCCCTGAA GATCATAGAT TCACTTCTTC AGAAATCAAT CATTTCTGGG TTGCAATAGG CATCATAGAC TCTAATCACC AAGGCGATAG GAATTACTTG GAAGAACTAG TGGACAATGG

Consensus ATTGTGGCCT TTTCCCcGAA GATCATaGaT TcACTTCTTC AGAAATCAAT CATTTCTGGG TTGCAaTAGG CATCATAGAC TCTaATCACC AAGcCGATAG GAATTACcTG GAAGAaCTAG TGGACAATGG

2471 2600

Lr1_Victo_allele TTTTCTCATG AAGAAGAAAG AGTATTATTT GGATGATCGA TGCAAACAAA AGGAATTTGA TTGCTATGTA ATGCATGATT TAATGCATGA GCTATCTAAG AGTGTTTCTG CACAAGAATG CCTCAATATA

TraesCS5D01G561200 TTTTCTCATG AAG------G AATTCGATTG GCGTGGTCAA TGC------- ---------- -TGGTATGTA ATGCATGATT TAATGCATGA GCTATCTAAC AGTGTTTCTG CACAAGAATG CCTCAACATA

Consensus TTTTCTCATG AAG......G AaTacgATTg GcaTGaTCaA TGC....... .......... .TGcTATGTA ATGCATGATT TAATGCATGA GCTATCTAAc AGTGTTTCTG CACAAGAATG CCTCAAcATA

2601 2730

Lr1_Victo_allele AGTGGCTTTG ATTTCAGAGC TGATGCCATC CCGCAATCTG TTCGACACTT ATCTATCAAC ATAGAAGACA GATATGATGC AAATTTTGAG GAAGAAATGT CTAAACTAAG GGAGAAGATA GACATTGCTA

TraesCS5D01G561200 AGTGGCTTAG ATTTCAGTGC TGATGCCATC CCGCGATCTG TCCGACACTT ATCTATCAAT GTAGAAGACA GATATGATGC AAATTTTGAG GAAGAAATGT CTAAATTAAG GGAGAAGATA GACATTGCTA

Consensus AGTGGCTTaG ATTTCAGaGC TGATGCCATC CCGCaATCTG TcCGACACTT ATCTATCAAc aTAGAAGACA GATATGATGC AAATTTTGAG GAAGAAATGT CTAAAcTAAG GGAGAAGATA GACATTGCTA

2731 2860

Lr1_Victo_allele ATGTGCGGAC TTTGATGATT TTTAGAGAAT ATGAAGAAGA AAGAACCGCC AAGATATTGA AAGATAGCTT CAAGGAAATA AATAGTCTGC GTGTCCTATT TATAGTGGTG AAGTCTGCAC AATCTTTTCC

TraesCS5D01G561200 ATGTGCGGAC TTTGATGATT TTTAGAGAAT ATGAAGAAGA AAGAACCGCC AAGATATTGA AAGATAGCTT CAAGGAAATA AATAGTCTGC GTGTCCTATT TATAGTGGTG AAGTCTGCAC AATCTTTTCT

Consensus ATGTGCGGAC TTTGATGATT TTTAGAGAAT ATGAAGAAGA AAGAACCGCC AAGATATTGA AAGATAGCTT CAAGGAAATA AATAGTCTGC GTGTCCTATT TATAGTGGTG AAGTCTGCAC AATCTTTTCc

2861 2990

Lr1_Victo_allele GGATATGTTT TCAAAACTTA TCCACCTCCA GTACCTCAAA ATTAGTTCAC CTCACATTGA CGGGGAAATG AGGTTACCTA GTACACTATC AAGATTTTAT CACTTGAAAT TCCTGGACCT AGATGATTGG

TraesCS5D01G561200 GGATATGTTT TCAAAACTTA TCCACCTCCA GTACCTCAAA ATTAGTTCAC CTCACATTGA CAGGGAAATG AGGTTACCTA GTACACTATC AAGATTTTAT CACTTGAAAT TCCTGGACCT AGATGATTGG

Consensus GGATATGTTT TCAAAACTTA TCCACCTCCA GTACCTCAAA ATTAGTTCAC CTCACATTGA CaGGGAAATG AGGTTACCTA GTACACTATC AAGATTTTAT CACTTGAAAT TCCTGGACCT AGATGATTGG

2991 3120

Lr1_Victo_allele CGTGGTAGTT CTGATTTGCC TGAAGACTTT AGCCACCTTG AGAATTTACA TGATTTCCGT GCTGAAAGTA AACTCCACTC CAATATTCGC AATGTTGGAA AGATGAAGCA TCTACAGAGG CTAGAAGAAT

TraesCS5D01G561200 AATGGTCGTT CTGATTTGCC TGAAGACTTT AGCCACCTTG AGAATTTACA TGATTTCCGT GCTGGAAGTG AACTCCACTC CAATATTCGC AATGTTGGAA AGATGAAGCA TCTACAGAGG CTAAAAGTAT

Consensus aaTGGTaGTT CTGATTTGCC TGAAGACTTT AGCCACCTTG AGAATTTACA TGATTTCCGT GCTGaAAGTa AACTCCACTC CAATATTCGC AATGTTGGAA AGATGAAGCA TCTACAGAGG CTAaAAGaAT

3121 3250

Lr1_Victo_allele TCCATGTTAA GAAGGAGAGC ATGGGATTTG AACTGTCAGA ACTTGGGCCA TTGACAGAGC TTGAAGGAGG ACTGACTGTA CGTGGTCTTG AACACGTGGC AACCAAGGAG GAAGCTACTG CAGCCAAACT

TraesCS5D01G561200 TCCATGTCAG GAAGGAGAGC ATGGGATTTG AACTGACAGA ACTTGGGGCA TTGACAGAGC TTGAAGGAGG ACTGATTATA CGTGGTCTTG AACACGTGGC AACCAAGGAG GAAGCTACTG CAGCCAAACT

Consensus TCCATGTcAa GAAGGAGAGC ATGGGATTTG AACTGaCAGA ACTTGGGcCA TTGACAGAGC TTGAAGGAGG ACTGAcTaTA CGTGGTCTTG AACACGTGGC AACCAAGGAG GAAGCTACTG CAGCCAAACT

3251 3380

Lr1_Victo_allele GATGTTGAAA AGGAATCTGA AGGAGTTGGA ATTACTCTGG GACAGAGATG GACCAACTAC AGATGCTGAT ATTCTTGATG CTCTTCAACC ACACTCTAAT CTTAGAGTAC TTGCAATTGT AAATCATGGT

TraesCS5D01G561200 GGTGTTGAAA AGGAACCTGA AGGAGTTGGA ATTACTCTGG GACAGAGATC AACCAACTAC AGATGCTGAT ATTCTTGATG CTCTTCAACC ACACTCTAAT CTTAGAGTAC TTACAATTGC AAATCATGGT

Consensus GaTGTTGAAA AGGAAcCTGA AGGAGTTGGA ATTACTCTGG GACAGAGATc aACCAACTAC AGATGCTGAT ATTCTTGATG CTCTTCAACC ACACTCTAAT CTTAGAGTAC TTaCAATTGc AAATCATGGT

3381 3510

Lr1_Victo_allele GGTACCGTTG GTCCTAGCTG GTTGTGTCTT GACATCTGGT TAACAAGTTT AGAGACTCTC ACTCTAGCAG GCGTATGTTG GAGCACTCTC CCGCCTTTTG CGAAGCTACC AAATCTCAAG GGACTCAAAC

TraesCS5D01G561200 GGTATGATTG GTCCTAGCTG GTTGTGTCTT GACATCTGGT TGACAAGTCT AGAGACTCTC ACTCTAGAAG GCGTATCTTG GAGCAACCTC CCACCTTTTG GGAAGCTACC AAATCTCAAG GGCCTCTATT

Consensus GGTAccaTTG GTCCTAGCTG GTTGTGTCTT GACATCTGGT TaACAAGTcT AGAGACTCTC ACTCTAGaAG GCGTATcTTG GAGCAacCTC CCaCCTTTTG cGAAGCTACC AAATCTCAAG GGaCTCaAac

3511 3640

Lr1_Victo_allele TGATGAGAAT TTCTGGAATG CATCAGTTTG GGTCTCTATG TGGTGGCACT CCAGGGAAAT GTTTTATGCG CTTGAAGACA GTTGAGTTTT ATGAGATGCC AGAACTTGCT GAATGGGTTG TGGAATCTAA

TraesCS5D01G561200 TGAATAAAAT TTCTGGAATG CATCAGTTTG GGCCTCTATG TGGTGGCGCT CCAGGGAAAT GTTTTATGCG CTTGAAGAAA GTTGGGTTTT ATGAGATGCC AGAACTTGCT GAATGGGTTG TGGAACCTAA

Consensus TGAagAaAAT TTCTGGAATG CATCAGTTTG GGcCTCTATG TGGTGGCaCT CCAGGGAAAT GTTTTATGCG CTTGAAGAaA GTTGaGTTTT ATGAGATGCC AGAACTTGCT GAATGGGTTG TGGAAcCTAA

3641 3770

Lr1_Victo_allele TTGCCATTCC TTTCCAAGTC TTGAAGAAAT CAGATGCAGA AATTGTCCCA ACCTCCGTGT GATGCCCTTC TCGGAGGTAT CTTTCACCAA TTTGCGCACA CTTTTTGTTT CCAGGTGCCC CAAGATGTCT

TraesCS5D01G561200 TTGCCATTCC TTTCCAAGTC TTGAAGAAAT CGAGTGCTTC AGTTGTCCCA ACCTCCGTGT GATGCCCTTC TCTGAGGTAT CTTGCACCAA TTTGCGCAGT CTTTATGTTT TTGGGTGCCC CAAGATGTCT

Consensus TTGCCATTCC TTTCCAAGTC TTGAAGAAAT CaaaTGCaga AaTTGTCCCA ACCTCCGTGT GATGCCCTTC TCgGAGGTAT CTTgCACCAA TTTGCGCAca CTTTaTGTTT ccaGGTGCCC CAAGATGTCT

3771 3900

Lr1_Victo_allele CTGCCCTCCA TGCCTCACAC CTCCACACTG ACAGATCTGA ATGTTGGAAT AGGTGATTCA GAAGGGTTGC ATTATGATGG AAAGAAATTG ATTGTTATAG GGTATGGCGG TGCTTTGGCC TCCCACAATC

TraesCS5D01G561200 CTGCCCACCA TGCCTCACAC CTCCACACTG ACAGATTTGG TTGTTGGAAT AGGTGATTCA GAAGGGTTGC GTTATGATGG AAAGAAATTG GTTGTTAGTG GGTATGGCGG TGCTTTGGCC TCCCACAATC

Consensus CTGCCCaCCA TGCCTCACAC CTCCACACTG ACAGATcTGa aTGTTGGAAT AGGTGATTCA GAAGGGTTGC aTTATGATGG AAAGAAATTG aTTGTTAgaG GGTATGGCGG TGCTTTGGCC TCCCACAATC

3901 4030

Lr1_Victo_allele TGGATACAGT AGAAGATATG ATTGTCGAAA GATGCGACGG TTTGTTCCCT GAAGATTTGG ATGGCAGTTT TGTCTTCCGT TCAGTTAAGA ATCTCACATT ACATGTATCT CGTCTTACCA GCAGCAAATC

TraesCS5D01G561200 TGGATAAAGT AG

Consensus TGGATAaAGT AG........ .......... .......... .......... .......... .......... .......... .......... .......... .......... .......... ..........

4031 4160

Lr1_Victo_allele ATCATCGTGA AAAGTGTTAA ACTGTTTCCC AGCTCTTTCT GTGTTGGTGA TAGTTGGCTA TGAGGAATGT GTAATGCAGT TCCCATCATC CAGCTCACTG CAGAAACTTA CCTTCTCAGG GTGTAAGGGC

TraesCS5D01G561200

Consensus .......... .......... .......... .......... .......... .......... .......... .......... .......... .......... .......... .......... ..........

4161 4290

Lr1_Victo_allele CTAGTTCTTG TGCCTGTGGA GAATGGAGGA GGAATTCAGG AGGACAAGTC ATTGCTCCAA TCATTAACCA TAGTCAGCTG TGGCGAATTG TTCTGTCGGT GGCCAATGAG AGAATCAGAG ACCATTTGCC

TraesCS5D01G561200

Consensus .......... .......... .......... .......... .......... .......... .......... .......... .......... .......... .......... .......... ..........

4291 4420

Lr1_Victo_allele CTTTCCCTGC TTCCCTGAGG GAACTTGATG TTTTCCAAGA GCCAAGCATG AAGTCAATGG CTCTGCTCTC AAACCTCACG TCTCTCACCA CTCTACAGCT AAACTACTGC AGTAATTTAA CAGTGGATGG

TraesCS5D01G561200

Consensus .......... .......... .......... .......... .......... .......... .......... .......... .......... .......... .......... .......... ..........

4421 4550

Lr1_Victo_allele ATTCAATCCT CTCATCGCAG TCAACCTCAT AGAGCTGCAA GTGCATAGGT GCAACACCTT AGCAGCAGAT ATGCTCTCAG AGGCGGCCTC TCACTCTCAG AGGGCCAAAT TATTGCCTGC AGGTTACATC

TraesCS5D01G561200

Consensus .......... .......... .......... .......... .......... .......... .......... .......... .......... .......... .......... .......... ..........

4551 4680

Lr1_Victo_allele TCTAGATTGG AGGTACTCAT CGTGGATAAC ATCTGTGGAT TGCTTGTTGC TCCTATTTGC ATCCTCCTCG CCCCGGCCCT CCACACACTT GTATTCTGGA TTGATGAAAC GATGGAAAGC TTGACGGAAG

TraesCS5D01G561200

Consensus .......... .......... .......... .......... .......... .......... .......... .......... .......... .......... .......... .......... ..........

4681 4810

Lr1_Victo_allele AGCAGGAGAA AGCGCTGCAG CTCCTCACCT CCCTCCAGAA TCTAACATTT TTCAGATGCA GGGGTCTACA GTCCCTTCCT CAAGGGTTGC ATCGCCTTTC TTCTCTCAAG GAGTTATGTG TCCGTGGGTG

TraesCS5D01G561200

Consensus .......... .......... .......... .......... .......... .......... .......... .......... .......... .......... .......... .......... ..........

4811 4940

Lr1_Victo_allele TCTAAAAATC CAATCGTTGC CCAAGGAGGG CCTCCCGCTT TCGCTGAGAA GACTAAAGAT GAATTGGCGC AGCGCTGAGA TAAACGAGCA AATTGAGAAA ATCAAAAGAA GCAACCCAGA TTTATCCGTC

TraesCS5D01G561200

Consensus .......... .......... .......... .......... .......... .......... .......... .......... .......... .......... .......... .......... ..........

4941 5070

Lr1_Victo_allele TCGTATTGCT AACTACACCC AAGGTAACAC TTGTCGCCTC CCTATTTTGA TTGTTTCTCT ATTTCTGATG AAACGAGGTT TATTCGCCAT CCATATATAT TTCTGCCTCA TCTTAACCAC TGTTGTCAAA

TraesCS5D01G561200

Consensus .......... .......... .......... .......... .......... .......... .......... .......... .......... .......... .......... .......... ..........

5071 5200

Lr1_Victo_allele TCTTACAGAC TAGCAGGTTC CACTGTTGCA ATCAATCTTT TGTGCACACA GGTTGTGAAA CCTGCACATT AATCAGCAGG TCAGGTGCGA GATACAACCT ACCGACTCTT CATTCTGCAA CGCAACACTA

TraesCS5D01G561200

Consensus .......... .......... .......... .......... .......... .......... .......... .......... .......... .......... .......... .......... ..........

5201 5330

Lr1_Victo_allele GCTGTGAACT CTTCATTCTG CTGCATGATC TGCCGGTTAG TACCTCTCCA CCTCCAGAAC CAAATTCCTT TTTTTAGAAA AGGAGGATAT GACCCCCGGC CTCTGCATCT GGGCGATGCA TACGGCCACT

TraesCS5D01G561200

Consensus .......... .......... .......... .......... .......... .......... .......... .......... .......... .......... .......... .......... ..........

5331 5460

Lr1_Victo_allele CCAGAACCAA ATTCCTGATC CTTCATTTAT GACTGCATTC ATTTCATTTA GGCCTCCTGA GTAGCTGCGT TATTGTTATA GGAGTTGCCT ATTATTCCAG CTTGTTCTTC ATTGTCTGAC CCAAGAATAG

TraesCS5D01G561200

Consensus .......... .......... .......... .......... .......... .......... .......... .......... .......... .......... .......... .......... ..........

5461 5475

Lr1_Victo_allele TTGGTTACCC GAATT

TraesCS5D01G561200

Consensus .......... .....

c)

1 130

Lr1_Victo_allele GCCCACACGC CCAAGCGTGA TGGCATCTTC CATATGTTTT ATCGGATGTT TGTTTATCTA CAAAACCTTC CACTCTCCCT AAAAAACCAA GGACTTGGAT GGTTCCGAAA TTTCCATATA AATGCAACCC

TraesCS5D01G561300

Consensus .......... .......... .......... .......... .......... .......... .......... .......... .......... .......... .......... .......... ..........

131 260

Lr1_Victo_allele GTGAAAATGT TGAGATATGT TCTTACACAT GCTCACCGCC AGTGGCAGAT CCGGGACCCA TGCCGGTGGG GCCTAGGCCT AGGGCATGAG AATGATTTAC TTCGTTGACC GCAGCTTATT GAGCACTGTA

TraesCS5D01G561300

Consensus .......... .......... .......... .......... .......... .......... .......... .......... .......... .......... .......... .......... ..........

261 390

Lr1_Victo_allele GCTGCACTGT AGCGCCTGGG GCCTGGGCTT GGCCCAATCC TGGGTCCGCC CCTGCTCACC GCCTAAAGGA GATACAAAAG AAATATTCTA AATGGCAGAA AAATCTTGGG ATTTTGAAAT CTAAATCATG

TraesCS5D01G561300

Consensus .......... .......... .......... .......... .......... .......... .......... .......... .......... .......... .......... .......... ..........

391 520

Lr1_Victo_allele TACTCACTCC GTCCCAAAAT AAGTGTTTCA ACTTTAGTAT AGTTTTGTAC TAAAGCTAAT ACAGAGTTGA GACACTTATT TTGAGACGGG AGGATATGTA TTTTCTGTTA TGTTTCGGAT TGTATCTCGA

TraesCS5D01G561300

Consensus .......... .......... .......... .......... .......... .......... .......... .......... .......... .......... .......... .......... ..........

521 650

Lr1_Victo_allele GAAGAATGTT CTTATATTAT GGAACGGGGA GTCTTATTGT TTGATTAATT ATTAATAAAG CCGACATTCT GTAAAGAAAC CTTTCAGCAG GGGTGTATTC GTAAAAGTTT TGCATTACCG GCTTGGGTCC

TraesCS5D01G561300

Consensus .......... .......... .......... .......... .......... .......... .......... .......... .......... .......... .......... .......... ..........

651 780

Lr1_Victo_allele CAATAATTTG TATCCATAGG TTGGTCAAAC AGCTCAAACC ATACCAACCA AGCAAGCAAG CAATCCCCAC ACCGCTAGAG CTACAGCTTC CACCGGCGTC AAAAGAAAAC ACCAATTATT GCCCCGGCGA

TraesCS5D01G561300

Consensus .......... .......... .......... .......... .......... .......... .......... .......... .......... .......... .......... .......... ..........

781 910

Lr1_Victo_allele CTCGACGTCC GGCGGCCGGC GGAGTGCAGA TCTACGGCTG TGCGGTGGCG TGGGCAGTTG GGGAAGAGTA CCCTGTCCTT GCTCCCCACA CGACACCGCT CCCCGGCGGC GTTGACCGAG CTCGGAGCTG

TraesCS5D01G561300 TA CACAAGTC-- GCCAGCTGCT -GCTTCCTCT CCACCACTAC ----TCCG-G CAAGGCA---

Consensus .......... .......... .......... .......... .......... .......... ........TA CaCaagcC.. GCcacCcaCa .GacaCCgCT CCaCcaCgaC ....aCCG.G CaaGGaa...

911 1040

Lr1_Victo_allele AGGCGAGGCG GCCGGCGGAG TGCAGATGGC GGCGGCTCTC GGCTCGGCGG CCACGCTCCT CGGCAAGGTG TTCACTATGC TGTCCGCCGC CCCGGTGGCG GCGTACGTGG ACAGCCTGGA GCTCGGCCAC

TraesCS5D01G561300 AGGCGGAGTG TCTGTAGAG- ---AGATGGA GGTGGCTCTC GGCTCGGCGG CCTCGCTCCT CGGCAAGCTG TTGAGGACGC TGTCCGACAG CCTGGTGGCG GTTTACGTGG ACAGCCTTCA GCTCGGCCAC

Consensus AGGCGaaGcG gCcGgaGaa. ...AGATGGa GGcGGCTCTC GGCTCGGCGG CCaCGCTCCT CGGCAAGcTG TTcAcgAcGC TGTCCGaCac CCcGGTGGCG GcgTACGTGG ACAGCCTgcA GCTCGGCCAC

1041 1170

Lr1_Victo_allele AACTCCCAGC AGATCAGGGC CAAGCTCGCG CACACGCGAG GCCTGCTGCA CAACGCCCAA GCCCAGGTGA GCGACGTCGG CCACAACCCT GGACTGCAGG AGCTGCTGCC GGCGCTGAGC AGGAACGCCG

TraesCS5D01G561300 AACTCGGAGC AGATCAAGGA CAAGCTGCTG CACGCGCAAG GCCTCCTGCA CAACGCCCAG GGGCAGGGGA GC-------- -CATAACCCT GGCCTGCAGG GGTTGCTGGA GAAGCTGAGC AGGGACGCCG

Consensus AACTCccAGC AGATCAaGGa CAAGCTcccG CACaCGCaAG GCCTcCTGCA CAACGCCCAa GccCAGGgGA GC........ .CAcAACCCT GGaCTGCAGG aGcTGCTGca GaaGCTGAGC AGGaACGCCG

1171 1300

Lr1_Victo_allele ACGAGGCGGA GGATCTGCTG GATGAGCTCC ACTACTTCCA GATCCATGAC AGGCTCCACG CCACCAACTA CGCCGCCACC CA---GGCCA ATTTT----- -CTC---CGT CATGCTCGCA ATGC------

TraesCS5D01G561300 ACCAGGCAGA GGACCTGCTG GATGTGCTCC ACTACTTCCA GATCCATGAC AGGCTCCACG GCACCAACTA CGCCGCCATC CAAGAGGCCG GCGTTGATGG CCTCAGGCAT CAAGCTCTCC ATGCCGGTAC

Consensus ACcAGGCaGA GGAcCTGCTG GATGaGCTCC ACTACTTCCA GATCCATGAC AGGCTCCACG cCACCAACTA CGCCGCCAcC CA...GGCCa acgTT..... .CTC...CaT CAaGCTCgCa ATGC......

1301 1430

Lr1_Victo_allele ---TCTCCGC CACAC--TGC ---------- --------CA CCAGCAGC-T GGGCCGC--- ---------- -------ATG CTTTTCTTGT T--CCTCTGC ACAAGA---- ---------- ---------T

TraesCS5D01G561300 TGCTCTTCGC CACACCTTGC TCCACTTCTT TTCTTGCTCG CCTACACCCA AGACCAAGAG GGATGGTAGT GATGATGATG CTGCTCTTGT CGGCCGCGTC ACCAAAAAAT CCAAAACCAA CTCTGCTAGT

Consensus ...TCTcCGC CACAC..TGC .......... ........Ca CCaaCAcC.a aGaCCaa... .......... .......ATG CTgcTCTTGT c..CCgCggC ACaAaA.... .......... .........T

1431 1560

Lr1_Victo_allele GATAGTGATT CTACCAGTGG GGATGATGAG TTACGTTTCC ACCGAGTGAT CTTCTCCAGA AAATTCAAGT CGGTGTTACA GGACATGCAG ACGCACTGTG ATTCCGTCTC TGATTTGCTC ----------

TraesCS5D01G561300 GCTAGTGCTA GTGCTGATGG TGATGATATG CTGCATTTCG ACAGAGTGTC CATGTCCAGC AAGATCAAGT CCCTGTTACA GGGCATGCAG TCCCACTGTG ATTCCGTCTC CAATTTGCTC CGCATCGGCA

Consensus GaTAGTGaTa cTaCcaaTGG gGATGATaaG cTaCaTTTCc ACaGAGTGac CaTcTCCAGa AAaaTCAAGT CccTGTTACA GGaCATGCAG aCcCACTGTG ATTCCGTCTC caATTTGCTC ..........

1561 1690

Lr1_Victo_allele --GGCACTAT CCCAACCAGC AGCA------ TGCCAGTTGC TGTACACCGG CCGCAGATTG GATCCACAAT TATACAAGAT ACATTGTATG GCAGGAGACA CACTTTTGAG GAAACTGTCA ACCGTATCTT

TraesCS5D01G561300 TCGGCAGTAT CCCAAGCAAC AGCACGGCAT TGTCAGTCGT CCTACATCGG CCTCAGACTG CTTCCATGAT TATACAAGAC ACATTGTATG GCAGGAGAGA CGTTTTTGAG GAAACTGTCA ATCGTATCAC

Consensus ..GGCAcTAT CCCAAcCAaC AGCA...... TGcCAGTcGc ccTACAcCGG CCgCAGAcTG caTCCAcaAT TATACAAGAc ACATTGTATG GCAGGAGAcA CacTTTTGAG GAAACTGTCA AcCGTATCac

1691 1820

Lr1_Victo_allele CA-------- GCTGCAAACA CCC------- ---TGTTTCT GTTCTTCCTA TAGTTGGTCC AGGGGGTATT GGAAAGACAA CTTTTGCTCA ACATCTGTAT AATGATGCAA GGACTGAAGA GCACTTCCAA

TraesCS5D01G561300 CACTCTTATA GTTGGTACCA CACAGACTGA GACTGTTTCT GTTCTTCCTA TAGTTGGTCC AGGGGGTATT GGAAAGACAA CTTTCACCAC TCACCTGTAC AATGACGCAA GGACTCAAGA CCACTTCCAA

Consensus CA........ GcTGcaAaCA CaC....... ...TGTTTCT GTTCTTCCTA TAGTTGGTCC AGGGGGTATT GGAAAGACAA CTTTcaCcaa aCAcCTGTAc AATGAcGCAA GGACTcAAGA cCACTTCCAA

1821 1950

Lr1_Victo_allele GTCAGGGTCT GGGTGTGTGT ATCCACTGAT TTCAATGTGC TTAAGCTCAC CAGGGAGATC CTTGCCTGCA TACCTGCAAC TGAAGAAGGA GGAAGCAGCA GTGTTGCAAA TGAAACAACC AATTTAGATC

TraesCS5D01G561300 GTCAGGGTGT GGGTATGTGT ATCCACTGAT TTCAATGTGC TTAAGCTCAC CAGGGAGATC CTTGGCTGCA TACCTGCAAC TGAAGGAGCA GGAAGCAGCG GTGTTGCAAA TGAAACAGCC AATTTAGACC

Consensus GTCAGGGTcT GGGTaTGTGT ATCCACTGAT TTCAATGTGC TTAAGCTCAC CAGGGAGATC CTTGcCTGCA TACCTGCAAC TGAAGaAGcA GGAAGCAGCa GTGTTGCAAA TGAAACAaCC AATTTAGAcC

1951 2080

Lr1_Victo_allele ATCTTCAGAG ATCCATTGTG CGCCGTCTCA AGTCCAAGAG GTTTCTAATT GTCTTGGACG ATATATGGAA ATGTGACAGT CAGGATCAGT GGAAAACCTT GTTAGCTCCC TTCACAAAGG GGGAAACCAA

TraesCS5D01G561300 AGCTTCAGAA ATCCATAGCA GAACGTCTCA AGTCCAAGAG GTTTCTAATT GTCTTGGATG ATATATGGAA ATGTGACAGT CAGGATCAGT GGAAAACCCT GGTAGCTCCG TTCACAAAGG GGGAAAGCAA

Consensus AgCTTCAGAa ATCCATaGca caaCGTCTCA AGTCCAAGAG GTTTCTAATT GTCTTGGAcG ATATATGGAA ATGTGACAGT CAGGATCAGT GGAAAACCcT GgTAGCTCCc TTCACAAAGG GGGAAAcCAA

2081 2210

Lr1_Victo_allele AGGAAGCATG CTACTTGTCA CAACTCGATT CCCAAAGCTA GCACAAATGA TGGAAACAAT TGATCCACTA GAGCTGCTAG GTTTGGAGTC TAATGACTTC TTCACATTCT TTGAAGCATG TATATTTGGT

TraesCS5D01G561300 AGGAAGTATG CTACTTGTCA CAACTCGATT CCCAAAGGTA GCAGACATGG TGAAAACAGT TGATCCACTA GAGCTGCGAG GTTTGGAGTC TAATGACTTC TTCACATTCT TTGAAGCATG TATATTTGGT

Consensus AGGAAGcATG CTACTTGTCA CAACTCGATT CCCAAAGcTA GCAcAaATGa TGaAAACAaT TGATCCACTA GAGCTGCgAG GTTTGGAGTC TAATGACTTC TTCACATTCT TTGAAGCATG TATATTTGGT

2211 2340

Lr1_Victo_allele GAAGACAACA AGCCAGAGCA TTTCGAAGAT GAGTTAGCTG GTATTGCACA AAAAATTGCA GACAAGCTAA AGGGTTCCCC GCTGGCAGCC AAAACAGTTG GTAGACTATT GCACAAGGAC CTTTCTCAGA

TraesCS5D01G561300 GAAGAAGAAA AGCCCGAGCA TTACCAAGAT GAGTTTGCTG GTATTGCACG AAAAATTGCA AACAAGCTAA AGGGTTCCCC GCTCGCAGCC AAAACAGTTG GTAGACTATT GCAGAAGGAC CTTTCTGAGG

Consensus GAAGAaaAaA AGCCaGAGCA TTaCcAAGAT GAGTTaGCTG GTATTGCACa AAAAATTGCA aACAAGCTAA AGGGTTCCCC GCTcGCAGCC AAAACAGTTG GTAGACTATT GCAcAAGGAC CTTTCTcAGa

2341 2470

Lr1_Victo_allele AACATTGGAA TGGAGTTCTT GAAAAGCATC AGTGGCTAAA GCAGCAAAAT AATGATGATA TCATGCCATC TTTAAAAATT AGCTATGATT GCCTCCCTTT TGATCTGAAG AAATGTTTTT CCTATTGTGG

TraesCS5D01G561300 AACATTGGCA TGGAGTTCTT GAAAAGCATC AGTGGCTAAA GCAGCAAGAA AATGATGATA TCATGCAATC TTTAAAGATT AGCTACGATT ACCTCCCATT TGATCTGAAG AAATGCTTTT CCTATTGTGG

Consensus AACATTGGaA TGGAGTTCTT GAAAAGCATC AGTGGCTAAA GCAGCAAaAa AATGATGATA TCATGCaATC TTTAAAaATT AGCTAcGATT aCCTCCCaTT TGATCTGAAG AAATGcTTTT CCTATTGTGG

2471 2600

Lr1_Victo_allele CCTTTTCCCC GAAGATCATT GGTTTACTTC TTCAGAAATC AATCATTTCT GGGTTGCAGT AGGCATCATA GACTCTGATC ACCAAGCCGA TAGGAATTAC CTGGAAGAGC TAGTGGACAA TGGTTTTCTC

TraesCS5D01G561300 CCTTTTCCCT GAAGATCATA GGTTTACTTC TTCAGAAATC AATCGTTTCT GGGTGGCAAC AGGCATCATA GACTCTGATC ATCAAGCCGA TAGGAATCAC ATGGAAGAAC TAGTGGACAA TGGTTTTCTC

Consensus CCTTTTCCCc GAAGATCATa GGTTTACTTC TTCAGAAATC AATCaTTTCT GGGTgGCAac AGGCATCATA GACTCTGATC AcCAAGCCGA TAGGAATcAC aTGGAAGAaC TAGTGGACAA TGGTTTTCTC

2601 2730

Lr1_Victo_allele ATGAAGAAGA AAGAGTATTA TTTGGATGAT CGATGCAAAC AAAAGGAATT TGATTGCTAT GTAATGCATG ATTTAATGCA TGAGCTATCT AAGAGTGTTT CTGCACAAGA ATGCCTCAAT ATAAGTGGCT

TraesCS5D01G561300 ATGAAGCAAT TCGA------ TTGGCGTGAT CGATG----- ---------- ---GTGGTAT GTAATGCATG ATTTAATGCA TGAGCTATCT AAGAGTGTTT CTGCACAAGA ATGCCACAAT ATAAGTGGCT

Consensus ATGAAGaAaa aaGA...... TTgGcaTGAT CGATG..... .......... ...gTGcTAT GTAATGCATG ATTTAATGCA TGAGCTATCT AAGAGTGTTT CTGCACAAGA ATGCCaCAAT ATAAGTGGCT

2731 2860

Lr1_Victo_allele TTGATTTCAG AGCTGATGCC ATCCCGCAAT CTGTTCGACA CTTATCTATC AACATAGAAG ACAGATATGA TGCAAATTTT GAGGAAGAAA TGTCTAAACT AAGGGAGAAG ATAGACATTG CTAATGTGCG

TraesCS5D01G561300 TTGATTTCAG AGCTGATGCC ATCTCGCAAT CTGTTCGGCA CTTATCTATC AACATAGAAG ACAGATATGA TGCAAATTTT GAGAAAGAAA TGTGTAAACT AAGGGAGAGG ATAGACATTG CTAATCTGCG

Consensus TTGATTTCAG AGCTGATGCC ATCcCGCAAT CTGTTCGaCA CTTATCTATC AACATAGAAG ACAGATATGA TGCAAATTTT GAGaAAGAAA TGTcTAAACT AAGGGAGAaG ATAGACATTG CTAATcTGCG

2861 2990

Lr1_Victo_allele GACTTTGATG ATTTTTAGAG AATATGAAGA AGAAAGAACC GCCAAGATAT TGAAAGATAG CTTCAAGGAA ATAAATAGTC TGCGTGTCCT ATTTATAGTG GTGAAGTCTG CACAATCTTT TCCGGATATG

TraesCS5D01G561300 GACTTTGATG ATTTGTAGAA GATATGAAGA AGAAAGAATT GCCAAGATTT TGAAAGATAG CTTCAAGGAA ATAAATAGTC TGCGTGTCCT ATTTATAGCA GTGAGTACTC CAGAATCTTT TCCATATAGG

Consensus GACTTTGATG ATTTgTAGAa aATATGAAGA AGAAAGAAcc GCCAAGATaT TGAAAGATAG CTTCAAGGAA ATAAATAGTC TGCGTGTCCT ATTTATAGca GTGAagaCTc CAcAATCTTT TCCagATAgG

2991 3120

Lr1_Victo_allele TTTTCAAAAC TTATCCACCT CCAGTACCTC AAAATTAGTT CACCTCACAT TGACGGGGAA ATGAGGTTAC CTAGTACACT ATCAAGATTT TATCACTTGA AATTCCTGGA CCTAGATGAT TGGCGTGGTA

TraesCS5D01G561300 TTTTCAAAAC TGATCCACCT CCAGTACCTC AAAATTAGTT CATCTTACAA AGACGGTGAA ATCAGTTTAC CTAGTACACT ATCAAGATTT TATCACTTGA AATTCCTGGA CCTAGATGAT TGGAGTGGTC

Consensus TTTTCAAAAC TgATCCACCT CCAGTACCTC AAAATTAGTT CAcCTcACAa aGACGGgGAA ATcAGgTTAC CTAGTACACT ATCAAGATTT TATCACTTGA AATTCCTGGA CCTAGATGAT TGGaGTGGTa

3121 3250

Lr1_Victo_allele GTTCTGATTT GCCTGAAGAC TTTAGCCACC TTGAGAATTT ACATGATTTC CGTGCTGAAA GTAAACTCCA CTCCAATATT CGCAATGTTG GAAAGATGAA GCATCTACAG AGGCTAGAAG AATTCCATGT

TraesCS5D01G561300 GTACTGATTT ACCTGAAGAC TTCAGCCACC TTGAGAATTT ACATGATTTC CGTGCTGGAA GTGAACTCCA CTCCAATATT CGCAATGTTG GAAAGATGAA GCATCTGCAG GAGCTAAAAG TATTCCATGT

Consensus GTaCTGATTT aCCTGAAGAC TTcAGCCACC TTGAGAATTT ACATGATTTC CGTGCTGaAA GTaAACTCCA CTCCAATATT CGCAATGTTG GAAAGATGAA GCATCTaCAG aaGCTAaAAG aATTCCATGT

3251 3380

Lr1_Victo_allele TAAGAAGGAG AGCATGGGAT TTGAACTGTC AGAACTTGGG CCATTGACAG AGCTTGAAGG AGGACTGACT GTACGTGGTC TTGAACACGT GGCAACCAAG GAGGAAGCTA CTGCAGCCAA ACTGATGTTG

TraesCS5D01G561300 CAGGAAGGAG AGCATGGGAT TTGAACTGAC AGAACTTGGG GCATTGCCAG AGCTTGAAGG AGGACTGATT ATACGTGGTC TTGAACACGT GGCAACCAAG GAGGAAGCTA CTGCAGCCAA ACTGATGTTG

Consensus cAaGAAGGAG AGCATGGGAT TTGAACTGaC AGAACTTGGG cCATTGaCAG AGCTTGAAGG AGGACTGAcT aTACGTGGTC TTGAACACGT GGCAACCAAG GAGGAAGCTA CTGCAGCCAA ACTGATGTTG

3381 3510

Lr1_Victo_allele AAAAGGAATC TGAAGGAGTT GGAATTACTC TGGGACAGAG ATGGACCAAC TACAGATGCT GATATTCTTG ATGCTCTTCA ACCACACTCT AATCTTAGAG TACTTGCAAT TGTAAATCAT GGTGGTACCG

TraesCS5D01G561300 AAAAGGAACC TGAAGGAGTT GGAATTACTC TGGGGCAGAG ATGGACCAAC TACAGATGAT GATATTCTTG ATGCTCTTCA ACCACACTCT AATCTTAGAG TACTTACAAT TGCAAATCAT GGTGGTATGA

Consensus AAAAGGAAcC TGAAGGAGTT GGAATTACTC TGGGaCAGAG ATGGACCAAC TACAGATGaT GATATTCTTG ATGCTCTTCA ACCACACTCT AATCTTAGAG TACTTaCAAT TGcAAATCAT GGTGGTAcca

3511 3640

Lr1_Victo_allele TTGGTCCTAG CTGGTTGTGT CTTGACATCT GGTTAACAAG TTTAGAGACT CTCACTCTAG CAGGCGTATG TTGGAGCACT CTCCCGCCTT TTGCGAAGCT ACCAAATCTC AAGGGACTCA AACTGATGAG

TraesCS5D01G561300 TTGGTCCTAG CTGGTTGTGT CTTGACATCT GGTTGACAAG TCTAGAGACT CTCACTCTAG AAGGCGTATC TTGGAGCAAC CTCCCACCCT TTGCGAAGCT ACCAAATCTC AAGGGCCTCT ATTTGAATAA

Consensus TTGGTCCTAG CTGGTTGTGT CTTGACATCT GGTTaACAAG TcTAGAGACT CTCACTCTAG aAGGCGTATc TTGGAGCAac CTCCCaCCcT TTGCGAAGCT ACCAAATCTC AAGGGaCTCa AacTGAagAa

3641 3770

Lr1_Victo_allele AATTTCTGGA ATGCATCAGT TTGGGTCTCT ATGTGGTGGC ACTCCAGGGA AATGTTTTAT GCGCTTGAAG ACAGTTGAGT TTTATGAGAT GCCAGAACTT GCTGAATGGG TTGTGGAATC TAATTGCCAT

TraesCS5D01G561300 AATTTCTGGA ATGCATCAGT TTGGGCCTCT ATGTGGTGGC GCTCCAGGGA AATGTTTTAT GCGCTTGAAG GAAGTTGGGT TTTATGAGAT GCCAGAACTT GCTGAATGGG TTGTGCTACC TAATTGCCAT

Consensus AATTTCTGGA ATGCATCAGT TTGGGcCTCT ATGTGGTGGC aCTCCAGGGA AATGTTTTAT GCGCTTGAAG aaAGTTGaGT TTTATGAGAT GCCAGAACTT GCTGAATGGG TTGTGcaAcC TAATTGCCAT

3771 3900

Lr1_Victo_allele TCCTTTCCAA GTCTTGAAGA AATCAGATGC AGAAATTGTC CCAACCTCCG TGTGATGCCC TTCTCGGAGG TATCTTTCAC CAATTTGCGC ACACTTTTTG TTTCCAGGTG CCCCAAGATG TCTCTGCCCT

TraesCS5D01G561300 TCCTTTCCAA GTCTTGAAGA AATCGAATGC GTCGATTGTC CCAACCTCCG TGTGATGCCC TTCTCTGAGG TATCTTGCAC CAATTTGCGC AGACTTTTTG TTTCTGGGTG CCCCAAGATG TCTCTGCCGT

Consensus TCCTTTCCAA GTCTTGAAGA AATCaaATGC agaaATTGTC CCAACCTCCG TGTGATGCCC TTCTCgGAGG TATCTTgCAC CAATTTGCGC AcACTTTTTG TTTCcaGGTG CCCCAAGATG TCTCTGCCcT

3901 4030

Lr1_Victo_allele CCATGCCTCA CACCTCCACA CTGACAGATC TGAATGTTGG AATAGGTG-- -------ATT CAGAAG---G GTTGCATTAT GATGGAAAGA AATTGA---- --TTGTTATA GGGTATGGCG GTGCTTTGGC

TraesCS5D01G561300 CCATGCCTCA CACATCCACA CTGACAGATT TGGTTGTTAA AAGAGATAGA ACATATAATT CAGAAACGTT GTTGTCTTAT GATGGAAAGG AATTGGAATT GGTTGTTAGT GGGTATGGCG GTGCTTTGGC

Consensus CCATGCCTCA CACaTCCACA CTGACAGATc TGaaTGTTaa AAgAGaTa.. .......ATT CAGAAa...g GTTGcaTTAT GATGGAAAGa AATTGa.... ..TTGTTAga GGGTATGGCG GTGCTTTGGC

4031 4160

Lr1_Victo_allele CTCCCACAAT CTGGATACAG TAGAAGATAT G--------- ---------- ---------- ---------- ---------- ---------- -------ATT GTCGAAAGAT GCGACGGTTT GTTCCCTGAA

TraesCS5D01G561300 CTACCACAAT CTGGATAAAG TAGAAGATAT GGATATTGAA AATGCATCGC ACATATCATT GACAGACATC GAAAAGTTTA AATCCCTAAC AAAAGTAACT GTCGGAAGAT GCGACGGTTT ATTCCCTGAA

Consensus CTaCCACAAT CTGGATAaAG TAGAAGATAT G......... .......... .......... .......... .......... .......... .......AcT GTCGaAAGAT GCGACGGTTT aTTCCCTGAA

4161 4290

Lr1_Victo_allele GATTTGGATG GCAGTTTTGT CTTCCGTTCA GTTAAGAATC TCACATTACA TGTATCTCGT CTTACCAGCA GCAAATCATC ATCGTGAAAA GTGTTAAACT GTTTCCCAGC TCTTTCTGTG TTGGTGATAG

TraesCS5D01G561300 GAGCTGGATG GCGGTTTTGT CTTCCCTTCA GTTGAGAGTC TCCAATTACA TGTATCTCAT CTTGCCAGCA ---AATCATC ATCTTCAAAA GTGTTAAACT GTTTCCCAGC TCTTTCTGTG TTGCACATAG

Consensus GAgcTGGATG GCaGTTTTGT CTTCCcTTCA GTTaAGAaTC TCaaATTACA TGTATCTCaT CTTaCCAGCA ...AATCATC ATCgTcAAAA GTGTTAAACT GTTTCCCAGC TCTTTCTGTG TTGcacATAG

4291 4420

Lr1_Victo_allele TTGGCTATGA GGA---ATGT GTAATGCAGT TCCCATCATC CAGCTCACTG CAGAAACTTA CCTTCTCAGG GTGTAAGGGC CTAGTTCTTG TGCCTGTGGA GAA------T GGAGGAGGAA TTCAGGAGGA

TraesCS5D01G561300 ATCACTGTGA GGAGGAATGT GTAATGCAGT TCCCATCATC CAGCTCACTG CAGAAAGTTA ACTTCTCATA CTGCAAGGGC CTGATTCTTG TGCCTCTGGA GAAGGAGAAT GGAGGAGGAA CTCAGGAGGA

Consensus aTcaCTaTGA GGA...ATGT GTAATGCAGT TCCCATCATC CAGCTCACTG CAGAAAcTTA aCTTCTCAga cTGcAAGGGC CTaaTTCTTG TGCCTcTGGA GAA......T GGAGGAGGAA cTCAGGAGGA

4421 4550

Lr1_Victo_allele CAAGTCATTG CTCCAATCAT TAACCATAGT CAGCTGTGGC GAATTGTTCT GTCGGTGGCC ------AATG AGAGAATCAG AGACCATTTG CCCTTTCCCT GCTTCCCTGA GGGAACTTGA TGTTTTCCAA

TraesCS5D01G561300 CAACTCATTG CTCCAATCAT TAACAATAAA GGGATGTGGC AAACTCTTCT CCCGTTGGCC CATGGGAATG GGAGAATCAG AGACCATTTG CCCTTTCCCT GCTTCCCTGA AGAAACTTGA TGTCGAAGGA

Consensus CAAcTCATTG CTCCAATCAT TAACaATAaa caGaTGTGGC aAAcTcTTCT ccCGgTGGCC ......AATG aGAGAATCAG AGACCATTTG CCCTTTCCCT GCTTCCCTGA aGaAACTTGA TGTcgaacaA

4551 4680

Lr1_Victo_allele GAGCCAAGCA TGAAGTCAAT GGCTCTGCTC TCAAACCTCA CGTCTCTCAC CACTCTACAG CTAAACTACT GCAGTAATTT AACAGTGGAT GGATTCAATC CTCTCATCGC AGTCAACCTC ATAGAGCTGC

TraesCS5D01G561300 GAGCCAAGCA TGAAGTCAAT GGCTCTGCTC TCAAACCTCA CGTCTCTCAC CACTCTAAAG CTAGAGGAGT GCGGTAATTT AACAGTGGAT GGATTCAATC CTCTCATCGC AGTCAACCTC AGAGAACTGC

Consensus GAGCCAAGCA TGAAGTCAAT GGCTCTGCTC TCAAACCTCA CGTCTCTCAC CACTCTAaAG CTAaAcgAcT GCaGTAATTT AACAGTGGAT GGATTCAATC CTCTCATCGC AGTCAACCTC AgAGAaCTGC

4681 4810

Lr1_Victo_allele AAGTGCATAG GTGCAACACC TTAGCAGCAG ATATGCTCTC AGAGGCGGCC TCTCACTCTC AGAGGGCCAA ATTATT---G CCTGCAGGTT ACATCTCTAG ATTGGAGGTA CTCATCGTGG ATAACATCTG

TraesCS5D01G561300 AAGTGTGTGG GTGCAACACC TTAGCAGCAG ATATGCTCTC AGAGGTGGCC TCTCACTCTC AGAGGGCCAA ATTATTATTG CCTGTAGGTT ACATCAGT-- ---------- ------GTGG ATAACATCTC

Consensus AAGTGcaTaG GTGCAACACC TTAGCAGCAG ATATGCTCTC AGAGGcGGCC TCTCACTCTC AGAGGGCCAA ATTATT...G CCTGcAGGTT ACATCacT.. .......... ......GTGG ATAACATCTc

4811 4940

Lr1_Victo_allele TGGATTGCTT GTTGCTCCTA TTTGCATCCT CCTCGCCCCG GCCCTCCACA CACTTGTATT CTGGATTG-- -ATGAAACGA TGGAAAGCTT GACGGAAGAG CAGGAGAAAG CGCTGCAGCT CCTCACCTCC

TraesCS5D01G561300 TGGATTGCTT GTTGCTCCTA TTTGCAGCCT CCTCGCCCCG GCCCTCCACA CACTTGAATT CAAGTATGCT GATGAGACCA TGGAAAGGTT GACGGAAGAG CAAGAGAAAG CGCTGCAGCT CCTCACCTCC

Consensus TGGATTGCTT GTTGCTCCTA TTTGCAgCCT CCTCGCCCCG GCCCTCCACA CACTTGaATT CaaGaaTG.. .ATGAaACcA TGGAAAGcTT GACGGAAGAG CAaGAGAAAG CGCTGCAGCT CCTCACCTCC

4941 5070

Lr1_Victo_allele CTCCAGAATC TAACATTTTT CAGATGCAGG GGTCTACAGT CCCTTCCTCA AGGGTTGCAT CGCCTTTCTT CTCTCAAGGA GTTATGTGTC CGTGGGTGTC TAAAAATCCA ATCGTTGCCC AAGGAGGGCC

TraesCS5D01G561300 CTCCAGACAC TAAGTTTTTA CTCCTGCAAG GGTCTGCAGT CCCTTCCTCA AGGGTTACAT CGCCTTTCTT CTCTCAAGGA GTTACATGTC ATGTACTGTC CAAATATCCG ATCGATGCCC AAGGAGGGCC

Consensus CTCCAGAaaC TAAcaTTTTa CacaTGCAaG GGTCTaCAGT CCCTTCCTCA AGGGTTaCAT CGCCTTTCTT CTCTCAAGGA GTTAcaTGTC agggacTGTC cAAAaATCCa ATCGaTGCCC AAGGAGGGCC

5071 5200

Lr1_Victo_allele TCCCGCTTTC GCTGAGAAGA CTAAAGATGA ATTGGCGCAG CGCTGAGATA AACGAGCAAA TTGAGAAAAT CAAAAGAAGC AACCCAGATT TATCCGTCTC GTATTGCTAA CTACACCCAA GGTAACACTT

TraesCS5D01G561300 TCCCGGTTTC GCTGAGAAAA CTATATATGA GTGATCGCAG CGCTGAGATA GATGAACAAA TTGAGAAAAT CAAAAGAACC AACCCAGATT TATCCGTCG- --AAACATAA CTACACCCAA GGTAACACTT

Consensus TCCCGcTTTC GCTGAGAAaA CTAaAgATGA aTgagCGCAG CGCTGAGATA aAcGAaCAAA TTGAGAAAAT CAAAAGAAcC AACCCAGATT TATCCGTCg. ..AaacaTAA CTACACCCAA GGTAACACTT

5201 5330

Lr1_Victo_allele GTCGCCTCCC TATTTTGATT GTTTCTCTAT TTCTGATGAA ACGAGGTTTA TTCGCCATCC ATATATATTT CTGCCTCATC TTAACCACTG TTGTCAAATC TTACAGACTA GCAG--GTTC CACTGTTGCA

TraesCS5D01G561300 GTCGCCTCCC TATTTTATTT TATTTTCTAT TTCTGATGAA GTGAGGTTTA TTTGCCATAT TTCTGT---T GTGCCTCATC TTAACCACTG TTGTCAAATC ATACAGACTA CTAGCGGTTC CACTC---CA

Consensus GTCGCCTCCC TATTTTaaTT gaTTcTCTAT TTCTGATGAA acGAGGTTTA TTcGCCATac aTaTaT...T cTGCCTCATC TTAACCACTG TTGTCAAATC aTACAGACTA ccAG..GTTC CACTc...CA

5331 5460

Lr1_Victo_allele ATCAATCTTT TGTGCACACA GGTTGTGAAA CCTGCACATT AATCAGCAGG TCAGGTGCGA GATACAACCT ACCGACTCTT CATTCTGCAA CGCAACACTA GCTGTGAACT CTTCATTCTG CTGCATGATC

TraesCS5D01G561300 CTCCACCACT GTTGCAATCA ATCTTTTATG CACAC

Consensus aTCaAcCacT ggTGCAaaCA agcTgTgAaa CacaC..... .......... .......... .......... .......... .......... .......... .......... .......... ..........

5461 5590

Lr1_Victo_allele TGCCGGTTAG TACCTCTCCA CCTCCAGAAC CAAATTCCTT TTTTTAGAAA AGGAGGATAT GACCCCCGGC CTCTGCATCT GGGCGATGCA TACGGCCACT CCAGAACCAA ATTCCTGATC CTTCATTTAT

TraesCS5D01G561300

Consensus .......... .......... .......... .......... .......... .......... .......... .......... .......... .......... .......... .......... ..........

5591 5705

Lr1_Victo_allele GACTGCATTC ATTTCATTTA GGCCTCCTGA GTAGCTGCGT TATTGTTATA GGAGTTGCCT ATTATTCCAG CTTGTTCTTC ATTGTCTGAC CCAAGAATAG TTGGTTACCC GAATT

TraesCS5D01G561300

Consensus .......... .......... .......... .......... .......... .......... .......... .......... .......... .......... .......... .....

d)

1 130

Lr1_Victo_allele GCCCACACGC CCAAGCGTGA TGGCATCTTC CATATGTTTT ATCGGATGTT TGTTTATCTA CAAAACCTTC CACTCTCCCT AAAAAACCAA GGACTTGGAT GGTTCCGAAA TTTCCATATA AATGCAACCC

Lr1_Glenlea_allele GCCCACACGC CCAAGCGTGA TGGCATCTTC CATATGTTTT ATCGGATGTT TGTTTATCTA CAAAACCTTC CACTCTCCCT AAAAAACCAA GGACTTGGAT GGTTCCGAAA TTTCCATATA AATGCAACCC

Consensus GCCCACACGC CCAAGCGTGA TGGCATCTTC CATATGTTTT ATCGGATGTT TGTTTATCTA CAAAACCTTC CACTCTCCCT AAAAAACCAA GGACTTGGAT GGTTCCGAAA TTTCCATATA AATGCAACCC

131 260

Lr1_Victo_allele GTGAAAATGT TGAGATATGT TCTTACACAT GCTCACCGCC AGTGGCAGAT CCGGGACCCA TGCCGGTGGG GCCTAGGCCT AGGGCATGAG AATGATTTAC TTCGTTGACC GCAGCTTATT GAGCACTGTA

Lr1_Glenlea_allele GTGAAAATGT TGAGATATGT TCTTACACAT GCTCACCGCC AGTGGCAGAT CCGGGACCCA TGCCGGTGGG GCCTAGGCCT AGGGCATGAG AATGATTTAC TTCGTTGACC GCAGCTTATT GAGCACTGTA

Consensus GTGAAAATGT TGAGATATGT TCTTACACAT GCTCACCGCC AGTGGCAGAT CCGGGACCCA TGCCGGTGGG GCCTAGGCCT AGGGCATGAG AATGATTTAC TTCGTTGACC GCAGCTTATT GAGCACTGTA

261 390

Lr1_Victo_allele GCTGCACTGT AGCGCCTGGG GCCTGGGCTT GGCCCAATCC TGGGTCCGCC CCTGCTCACC GCCTAAAGGA GATACAAAAG AAATATTCTA AATGGCAGAA AAATCTTGGG ATTTTGAAAT CTAAATCATG

Lr1_Glenlea_allele GCTGCACTGT AGCGCCTGGG GCCTGGGCTT GGCCCAATCC TGGGTCCGCC CCTGCTCACC GCCTAAAGGA GATACAAAAG AAATATTCTA AATGGCAGAA AAATCTTGGG ATTTTGAAAT CTAAATCATG

Consensus GCTGCACTGT AGCGCCTGGG GCCTGGGCTT GGCCCAATCC TGGGTCCGCC CCTGCTCACC GCCTAAAGGA GATACAAAAG AAATATTCTA AATGGCAGAA AAATCTTGGG ATTTTGAAAT CTAAATCATG

391 520

Lr1_Victo_allele TACTCACTCC GTCCCAAAAT AAGTGTTTCA ACTTTAGTAT AGTTTTGTAC TAAAGCTAAT ACAGAGTTGA GACACTTATT TTGAGACGGG AGGATATGTA TTTTCTGTTA TGTTTCGGAT TGTATCTCGA

Lr1_Glenlea_allele TACTCACTCC GTCCCAAAAT AAGTGTTTCA ACTTTAGTAT AGTTTTGTAC TAAAGCTAAT ACAGAGTTGA GACACTTATT TTGAGACGGG AGGATATGTA TTTTCTGTTA TGTTTCGGAT TGTATCTCGA

Consensus TACTCACTCC GTCCCAAAAT AAGTGTTTCA ACTTTAGTAT AGTTTTGTAC TAAAGCTAAT ACAGAGTTGA GACACTTATT TTGAGACGGG AGGATATGTA TTTTCTGTTA TGTTTCGGAT TGTATCTCGA

521 650

Lr1_Victo_allele GAAGAATGTT CTTATATTAT GGAACGGGGA GTCTTATTGT TTGATTAATT ATTAATAAAG CCGACATTCT GTAAAGAAAC CTTTCAGCAG GGGTGTATTC GTAAAAGTTT TGCATTACCG GCTTGGGTCC

Lr1_Glenlea_allele GAAGAATGTT CTTATATTAT GGAACGGGGA GTCTTATTGT TTGATTAATT ATTAATAAAG CCGACATTCT GTAAAGAAAC CTTTCAGCAG GGGTGTATTC GTAAAAGTTT TGCATTACCG GCTTGGGTCC

Consensus GAAGAATGTT CTTATATTAT GGAACGGGGA GTCTTATTGT TTGATTAATT ATTAATAAAG CCGACATTCT GTAAAGAAAC CTTTCAGCAG GGGTGTATTC GTAAAAGTTT TGCATTACCG GCTTGGGTCC

651 780

Lr1_Victo_allele CAATAATTTG TATCCATAGG TTGGTCAAAC AGCTCAAACC ATACCAACCA AGCAAGCAAG CAATCCCCAC ACCGCTAGAG CTACAGCTTC CACCGGCGTC AAAAGAAAAC ACCAATTATT GCCCCGGCGA

Lr1_Glenlea_allele CAATAATTTG TATCCATAGG TTGGTCAAAC AGCTCAAACC ATACCAACCA AGCAAGCAAG CAATCCCCAC ACCGCTAGAG CTACAGCTTC CACCGGCGTC AAAAGAAAAC ACCAATTATT GCCCCGGCGA

Consensus CAATAATTTG TATCCATAGG TTGGTCAAAC AGCTCAAACC ATACCAACCA AGCAAGCAAG CAATCCCCAC ACCGCTAGAG CTACAGCTTC CACCGGCGTC AAAAGAAAAC ACCAATTATT GCCCCGGCGA

781 910

Lr1_Victo_allele CTCGACGTCC GGCGGCCGGC GGAGTGCAGA TCTACGGCTG TGCGGTGGCG TGGGCAGTTG GGGAAGAGTA CCCTGTCCTT GCTCCCCACA CGACACCGCT CCCCGGCGGC GTTGACCGAG CTCGGAGCTG

Lr1_Glenlea_allele CTCGACGTCC GGCGGCCGGC GGAGTGCAGA TCTACGGCTG TGCGGTGGCG TGGGCAGTTG GGGAAGAGTA CCCTGTCCTT GCTCCCCACA CGACACCGCT CCCCGGCGGC GTTGACCGAG CTCGGAGCTG

Consensus CTCGACGTCC GGCGGCCGGC GGAGTGCAGA TCTACGGCTG TGCGGTGGCG TGGGCAGTTG GGGAAGAGTA CCCTGTCCTT GCTCCCCACA CGACACCGCT CCCCGGCGGC GTTGACCGAG CTCGGAGCTG

911 1040

Lr1_Victo_allele AGGCGAGGCG GCCGGCGGAG TGCAGATGGC GGCGGCTCTC GGCTCGGCGG CCACGCTCCT CGGCAAGGTG TTCACTATGC TGTCCGCCGC CCCGGTGGCG GCGTACGTGG ACAGCCTGGA GCTCGGCCAC

Lr1_Glenlea_allele AGGCGAGGCG GCCGGCGGAG TGCAGATGGC GGCGGCTCTC GGCTCGGCGG CCACGCTCCT CGGCAAGGTG TTCACTATGC TGTCCGCCGC CCCGGTGGCG GCGTACGTGG ACAGCCTGGA GCTCGGCCAC

Consensus AGGCGAGGCG GCCGGCGGAG TGCAGATGGC GGCGGCTCTC GGCTCGGCGG CCACGCTCCT CGGCAAGGTG TTCACTATGC TGTCCGCCGC CCCGGTGGCG GCGTACGTGG ACAGCCTGGA GCTCGGCCAC

1041 1170

Lr1_Victo_allele AACTCCCAGC AGATCAGGGC CAAGCTCGCG CACACGCGAG GCCTGCTGCA CAACGCCCAA GCCCAGGTGA GCGACGTCGG CCACAACCCT GGACTGCAGG AGCTGCTGCC GGCGCTGAGC AGGAACGCCG

Lr1_Glenlea_allele AACTCCCAGC AGATCAGGGC CAAGCTCGCG CACACGCGAG GCCTGCTGCA CAACGCCCAA GCCCAGGTGA GCGACGTCGG CCACAACCCT GGACTGCAGG AGCTGCTGCC GGCGCTGAGC AGGAACGCCG

Consensus AACTCCCAGC AGATCAGGGC CAAGCTCGCG CACACGCGAG GCCTGCTGCA CAACGCCCAA GCCCAGGTGA GCGACGTCGG CCACAACCCT GGACTGCAGG AGCTGCTGCC GGCGCTGAGC AGGAACGCCG

1171 1300

Lr1_Victo_allele ACGAGGCGGA GGATCTGCTG GATGAGCTCC ACTACTTCCA GATCCATGAC AGGCTCCACG CCACCAACTA CGCCGCCACC CAGGCCAATT TTCTCCGTCA TGCTCGCAAT GCTCTCCGCC ACACTGCCAC

Lr1_Glenlea_allele ACGAGGCGGA GGATCTGCTG GATGAGCTCC ACTACTTCCA GATCCATGAC AGGCTCCACG CCACCAACTA CGCCGCCACC CAGGCCAATT TTCTCCGTCA TGCTCGCAAT GCTCTCCGCC ACACTGCCAC

Consensus ACGAGGCGGA GGATCTGCTG GATGAGCTCC ACTACTTCCA GATCCATGAC AGGCTCCACG CCACCAACTA CGCCGCCACC CAGGCCAATT TTCTCCGTCA TGCTCGCAAT GCTCTCCGCC ACACTGCCAC

1301 1430

Lr1_Victo_allele CAGCAGCTGG GCCGCATGCT TTTCTTGTTC CTCTGCACAA GATGATAGTG ATTCTACCAG TGGGGATGAT GAGTTACGTT TCCACCGAGT GATCTTCTCC AGAAAATTCA AGTCGGTGTT ACAGGACATG

Lr1_Glenlea_allele CAGCAGCTGG GCCGCATGCT TTTCTTGTTC CTCTGCACAA GATGATAGTG ATTCTACCAG TGGGGATGAT GAGTTACGTT TCCACCGAGT GATCTTCTCC AGAAAATTCA AGTCGGTGTT ACAGGACATG

Consensus CAGCAGCTGG GCCGCATGCT TTTCTTGTTC CTCTGCACAA GATGATAGTG ATTCTACCAG TGGGGATGAT GAGTTACGTT TCCACCGAGT GATCTTCTCC AGAAAATTCA AGTCGGTGTT ACAGGACATG

1431 1560

Lr1_Victo_allele CAGACGCACT GTGATTCCGT CTCTGATTTG CTCGGCACTA TCCCAACCAG CAGCATGCCA GTTGCTGTAC ACCGGCCGCA GATTGGATCC ACAATTATAC AAGATACATT GTATGGCAGG AGACACACTT

Lr1_Glenlea_allele CAGACGCACT GTGATTCCGT CTCTGATTTG CTCGGCACTA TCCCAACCAG CAGCATGCCA GTTGCTGTAC ACCGGCCGCA GATTGGATCC ACAATTATAC AAGATACATT GTATGGCAGG AGACACACTT

Consensus CAGACGCACT GTGATTCCGT CTCTGATTTG CTCGGCACTA TCCCAACCAG CAGCATGCCA GTTGCTGTAC ACCGGCCGCA GATTGGATCC ACAATTATAC AAGATACATT GTATGGCAGG AGACACACTT

1561 1690

Lr1_Victo_allele TTGAGGAAAC TGTCAACCGT ATCTTCAGCT GCAAACACCC TGTTTCTGTT CTTCCTATAG TTGGTCCAGG GGGTATTGGA AAGACAACTT TTGCTCAACA TCTGTATAAT GATGCAAGGA CTGAAGAGCA

Lr1_Glenlea_allele TTGAGGAAAC TGTCAACCGT ATCTTCAGCT GCAAACACCC TGTTTCTGTT CTTCCTATAG TTGGTCCAGG GGGTATTGGA AAGACAACTT TTGCTCAACA TCTGTATAAT GATGCAAGGA CTGAAGAGCA

Consensus TTGAGGAAAC TGTCAACCGT ATCTTCAGCT GCAAACACCC TGTTTCTGTT CTTCCTATAG TTGGTCCAGG GGGTATTGGA AAGACAACTT TTGCTCAACA TCTGTATAAT GATGCAAGGA CTGAAGAGCA

1691 1820

Lr1_Victo_allele CTTCCAAGTC AGGGTCTGGG TGTGTGTATC CACTGATTTC AATGTGCTTA AGCTCACCAG GGAGATCCTT GCCTGCATAC CTGCAACTGA AGAAGGAGGA AGCAGCAGTG TTGCAAATGA AACAACCAAT

Lr1_Glenlea_allele CTTCCAAGTC AGGGTCTGGG TGTGTGTATC CACTGATTTC AATGTGCTTA AGCTCACCAG GGAGATCCTT GCCTGCATAC CTGCAACTGA AGAAGGAGGA AGCAGCAGTG TTGCAAATGA AACAACCAAT

Consensus CTTCCAAGTC AGGGTCTGGG TGTGTGTATC CACTGATTTC AATGTGCTTA AGCTCACCAG GGAGATCCTT GCCTGCATAC CTGCAACTGA AGAAGGAGGA AGCAGCAGTG TTGCAAATGA AACAACCAAT

1821 1950

Lr1_Victo_allele TTAGATCATC TTCAGAGATC CATTGTGCGC CGTCTCAAGT CCAAGAGGTT TCTAATTGTC TTGGACGATA TATGGAAATG TGACAGTCAG GATCAGTGGA AAACCTTGTT AGCTCCCTTC ACAAAGGGGG

Lr1_Glenlea_allele TTAGATCATC TTCAGAGATC CATTGTGCGC CGTCTCAAGT CCAAGAGGTT TCTAATTGTC TTGGACGATA TATGGAAATG TGACAGTCAG GATCAGTGGA AAACCTTGTT AGCTCCCTTC ACAAAGGGGG

Consensus TTAGATCATC TTCAGAGATC CATTGTGCGC CGTCTCAAGT CCAAGAGGTT TCTAATTGTC TTGGACGATA TATGGAAATG TGACAGTCAG GATCAGTGGA AAACCTTGTT AGCTCCCTTC ACAAAGGGGG

1951 2080

Lr1_Victo_allele AAACCAAAGG AAGCATGCTA CTTGTCACAA CTCGATTCCC AAAGCTAGCA CAAATGATGG AAACAATTGA TCCACTAGAG CTGCTAGGTT TGGAGTCTAA TGACTTCTTC ACATTCTTTG AAGCATGTAT

Lr1_Glenlea_allele AAACCAAAGG AAGCATGCTA CTTGTCACAA CTCGATTTCC AAAGCTAGCA CAAATGATGG AAACAATTGA TCCACTAGAG CTGCTAGGTT TGGAGTCTAA TGACTTCTTC ACATTCTTTG AAGCATGTAT

Consensus AAACCAAAGG AAGCATGCTA CTTGTCACAA CTCGATTcCC AAAGCTAGCA CAAATGATGG AAACAATTGA TCCACTAGAG CTGCTAGGTT TGGAGTCTAA TGACTTCTTC ACATTCTTTG AAGCATGTAT

2081 2210

Lr1_Victo_allele ATTTGGTGAA GACAACAAGC CAGAGCATTT CGAAGATGAG TTAGCTGGTA TTGCACAAAA AATTGCAGAC AAGCTAAAGG GTTCCCCGCT GGCAGCCAAA ACAGTTGGTA GACTATTGCA CAAGGACCTT

Lr1_Glenlea_allele ATTTGGTGAA GACAACAAGC CAGAGCATTT CGAAGATGAG TTAGCTGGTA TTGCACAAAA AATTGCAGAC AAGCTAAAGG GTTCCCCGCT GGCAGCCAAA ACAGTTGGTA GACTATTGCA CAAGGACCTT

Consensus ATTTGGTGAA GACAACAAGC CAGAGCATTT CGAAGATGAG TTAGCTGGTA TTGCACAAAA AATTGCAGAC AAGCTAAAGG GTTCCCCGCT GGCAGCCAAA ACAGTTGGTA GACTATTGCA CAAGGACCTT

2211 2340

Lr1_Victo_allele TCTCAGAAAC ATTGGAATGG AGTTCTTGAA AAGCATCAGT GGCTAAAGCA GCAAAATAAT GATGATATCA TGCCATCTTT AAAAATTAGC TATGATTGCC TCCCTTTTGA TCTGAAGAAA TGTTTTTCCT

Lr1_Glenlea_allele TCTCAGAAAC ATTGGAATGG AGTTCTTGAA AAGCATCAGT GGCTAAAGCA GCAAAATAAT GATGATATCA TGCCATCTTT AAAAATTAGC TATGATTGCC TCCCTTTTGA TCTGAAGAAA TGTTTTTCCT

Consensus TCTCAGAAAC ATTGGAATGG AGTTCTTGAA AAGCATCAGT GGCTAAAGCA GCAAAATAAT GATGATATCA TGCCATCTTT AAAAATTAGC TATGATTGCC TCCCTTTTGA TCTGAAGAAA TGTTTTTCCT

2341 2470

Lr1_Victo_allele ATTGTGGCCT TTTCCCCGAA GATCATTGGT TTACTTCTTC AGAAATCAAT CATTTCTGGG TTGCAGTAGG CATCATAGAC TCTGATCACC AAGCCGATAG GAATTACCTG GAAGAGCTAG TGGACAATGG

Lr1_Glenlea_allele ATTGTGGCCT TTTCCCCGAA GATCATTGGT TTACTTCTTC AGAAATCAAT CATTTCTGGG TTGCAGTAGG CATCATAGAC TCTGATCACC AAGCCGATAG GAATTACCTG GAAGAGCTAG TGGACAATGG

Consensus ATTGTGGCCT TTTCCCCGAA GATCATTGGT TTACTTCTTC AGAAATCAAT CATTTCTGGG TTGCAGTAGG CATCATAGAC TCTGATCACC AAGCCGATAG GAATTACCTG GAAGAGCTAG TGGACAATGG

2471 2600

Lr1_Victo_allele TTTTCTCATG AAGAAGAAAG AGTATTATTT GGATGATCGA TGCAAACAAA AGGAATTTGA TTGCTATGTA ATGCATGATT TAATGCATGA GCTATCTAAG AGTGTTTCTG CACAAGAATG CCTCAATATA

Lr1_Glenlea_allele TTTTCTCATG AAGAAGAAAG AGTATTATTT GGATGATCGA TGCAAACAAA AGGAATTTGA TTGCTATGTA ATGCATGATT TAATGCATGA GCTATCTAAG AGTGTTTCTG CACAAGAATG CCTCAATATA

Consensus TTTTCTCATG AAGAAGAAAG AGTATTATTT GGATGATCGA TGCAAACAAA AGGAATTTGA TTGCTATGTA ATGCATGATT TAATGCATGA GCTATCTAAG AGTGTTTCTG CACAAGAATG CCTCAATATA

2601 2730

Lr1_Victo_allele AGTGGCTTTG ATTTCAGAGC TGATGCCATC CCGCAATCTG TTCGACACTT ATCTATCAAC ATAGAAGACA GATATGATGC AAATTTTGAG GAAGAAATGT CTAAACTAAG GGAGAAGATA GACATTGCTA

Lr1_Glenlea_allele AGTGGCTTTG ATTTCAGAGC TGATGCCATC CCGCAATCTG TTCGACACTT ATCTATCAAC ATAGAAGACA GATATGATGC AAATTTTGAG GAAGAAATGT CTAAACTAAG GGAGAAGATA GACATTGCTA

Consensus AGTGGCTTTG ATTTCAGAGC TGATGCCATC CCGCAATCTG TTCGACACTT ATCTATCAAC ATAGAAGACA GATATGATGC AAATTTTGAG GAAGAAATGT CTAAACTAAG GGAGAAGATA GACATTGCTA

2731 2860

Lr1_Victo_allele ATGTGCGGAC TTTGATGATT TTTAGAGAAT ATGAAGAAGA AAGAACCGCC AAGATATTGA AAGATAGCTT CAAGGAAATA AATAGTCTGC GTGTCCTATT TATAGTGGTG AAGTCTGCAC AATCTTTTCC

Lr1_Glenlea_allele ATGTGCGGAC TTTGATGATT TTTAGAGAAT ATGAAGAAGA AAGAACCGCC AAGATATTGA AAGATAGCTT CAAGGAAATA AATAGTCTGC GTGTCCTATT TATAGTGGTG AAGTCTGCAC AATCTTTTCC

Consensus ATGTGCGGAC TTTGATGATT TTTAGAGAAT ATGAAGAAGA AAGAACCGCC AAGATATTGA AAGATAGCTT CAAGGAAATA AATAGTCTGC GTGTCCTATT TATAGTGGTG AAGTCTGCAC AATCTTTTCC

2861 2990

Lr1_Victo_allele GGATATGTTT TCAAAACTTA TCCACCTCCA GTACCTCAAA ATTAGTTCAC CTCACATTGA CGGGGAAATG AGGTTACCTA GTACACTATC AAGATTTTAT CACTTGAAAT TCCTGGACCT AGATGATTGG

Lr1_Glenlea_allele GGATATGTTT TCAAAACTTA TCCACCTCCA GTACCTCAAA ATTAGTTCAC CTCACATTGA CGGGGAAATG AGGTTACCTA GTACACTATC AAGATTTTAT CACTTGAAAT TCCTGGACCT AGATGATTGG

Consensus GGATATGTTT TCAAAACTTA TCCACCTCCA GTACCTCAAA ATTAGTTCAC CTCACATTGA CGGGGAAATG AGGTTACCTA GTACACTATC AAGATTTTAT CACTTGAAAT TCCTGGACCT AGATGATTGG

2991 3120

Lr1_Victo_allele CGTGGTAGTT CTGATTTGCC TGAAGACTTT AGCCACCTTG AGAATTTACA TGATTTCCGT GCTGAAAGTA AACTCCACTC CAATATTCGC AATGTTGGAA AGATGAAGCA TCTACAGAGG CTAGAAGAAT

Lr1_Glenlea_allele CGTGGTAGTT CTGATTTGCC TGAAGACTTT AGCCACCTTG AGAATTTACA TGATTTCCGT GCTGAAAGTA AACTCCACTC CAATATTCGC AATGTTGGAA AGATGAAGCA TCTACAGAGG CTAGAAGAAT

Consensus CGTGGTAGTT CTGATTTGCC TGAAGACTTT AGCCACCTTG AGAATTTACA TGATTTCCGT GCTGAAAGTA AACTCCACTC CAATATTCGC AATGTTGGAA AGATGAAGCA TCTACAGAGG CTAGAAGAAT

3121 3250

Lr1_Victo_allele TCCATGTTAA GAAGGAGAGC ATGGGATTTG AACTGTCAGA ACTTGGGCCA TTGACAGAGC TTGAAGGAGG ACTGACTGTA CGTGGTCTTG AACACGTGGC AACCAAGGAG GAAGCTACTG CAGCCAAACT

Lr1_Glenlea_allele TCCATGTTAA GAAGGAGAGC ATGGGATTTG AACTGTCAGA ACTTGGGCCA TTGACAGAGC TTGAAGGAGG ACTGACTGTA CGTGGTCTTG AACACGTGGC AACCAAGGAG GAAGCTACTG CAGCCAAACT

Consensus TCCATGTTAA GAAGGAGAGC ATGGGATTTG AACTGTCAGA ACTTGGGCCA TTGACAGAGC TTGAAGGAGG ACTGACTGTA CGTGGTCTTG AACACGTGGC AACCAAGGAG GAAGCTACTG CAGCCAAACT

3251 3380

Lr1_Victo_allele GATGTTGAAA AGGAATCTGA AGGAGTTGGA ATTACTCTGG GACAGAGA-- ----TGGACC AACTACAGAT GCTGATATTC TTGATGCTCT TCAACCACAC TCTAATCTTA GAGTACTTGC AATTGTAAAT

Lr1_Glenlea_allele GATGTTGAAA AGGAATCTGA AGCAGTTGGA ATTACTCTGG GACAGAGACC TTGGTGGACC AACTACAGAT GCTGATATTC TTGATGCTCT TCAACCACAC TCTAATCTTA GAGTACTTGC AATTGTAAAT

Consensus GATGTTGAAA AGGAATCTGA AGcAGTTGGA ATTACTCTGG GACAGAGA.. ....TGGACC AACTACAGAT GCTGATATTC TTGATGCTCT TCAACCACAC TCTAATCTTA GAGTACTTGC AATTGTAAAT

3381 3510

Lr1_Victo_allele CATGGTGGTA CCGTTGGTCC TAGCTGGTTG TGTCTTGACA TCTGGTTAAC AAGTTTAGAG ACTCTCACTC TAGCAGGCGT ATGTTGGAGC ACTCTCCCGC CTTTTGCGAA GCTACCAAAT CTCAAGGGAC

Lr1_Glenlea_allele CATGGTGGTA CCGTTGGTCC TAGCTGGTTG TGTCTTGACA TCTGGTTAAC AAGTTTAGAG ACTCTCACTC TAGCAGGCGT ATGTTGGAGC ACTCTCCCGC CTTTTGCGAA GCTACCAAAT CTCAAGGGAC

Consensus CATGGTGGTA CCGTTGGTCC TAGCTGGTTG TGTCTTGACA TCTGGTTAAC AAGTTTAGAG ACTCTCACTC TAGCAGGCGT ATGTTGGAGC ACTCTCCCGC CTTTTGCGAA GCTACCAAAT CTCAAGGGAC

3511 3640

Lr1_Victo_allele TCAAACTGAT GAGAATTTCT GGAATGCATC AGTTTGGGTC TCTATGTGGT GGCACTCCAG GGAAATGTTT TATGCGCTTG AAGACAGTTG AGTTTTATGA GATGCCAGAA CTTGCTGAAT GGGTTGTGGA

Lr1_Glenlea_allele TCAAACTGAT GAGAATTTCT GGAATGCATC AGTTTGGGTC TCTATGTGGT GGCACTCCAG GGAAATGTTT TATGCGCTTG AAGACAGTTG AGTTTTATGA GATGCCAGAA CTTGCTGAAT GGGTTGTGGA

Consensus TCAAACTGAT GAGAATTTCT GGAATGCATC AGTTTGGGTC TCTATGTGGT GGCACTCCAG GGAAATGTTT TATGCGCTTG AAGACAGTTG AGTTTTATGA GATGCCAGAA CTTGCTGAAT GGGTTGTGGA

3641 3770

Lr1_Victo_allele ATCTAATTGC CATTCCTTTC CAAGTCTTGA AGAAATCAGA TGCAGAAATT GTCCCAACCT CCGTGTGATG CCCTTCTCGG AGGTATCTTT CACCAATTTG CGCACACTTT TTGTTTCCAG GTGCCCCAAG

Lr1_Glenlea_allele ATCTAATTGC CATTCCTTTC CAAGTCTTGA AGAAATCAGA TGCAGAAATT GTCCCAACCT CCGTGTGATG CCCTTCTCGG AGGTATCTTT CACCAATTTG CGCACACTTT TTGTTTCCAG GTGCCCCAAG

Consensus ATCTAATTGC CATTCCTTTC CAAGTCTTGA AGAAATCAGA TGCAGAAATT GTCCCAACCT CCGTGTGATG CCCTTCTCGG AGGTATCTTT CACCAATTTG CGCACACTTT TTGTTTCCAG GTGCCCCAAG

3771 3900

Lr1_Victo_allele ATGTCTCTGC CCTCCATGCC TCACACCTCC ACACTGACAG ATCTGAATGT TGGAATAGGT GATTCAGAAG GGTTGCATTA TGATGGAAAG AAATTGATTG TTATAGGGTA TGGCGGTGCT TTGGCCTCCC

Lr1_Glenlea_allele ATGTCTCTGC CCTCCATGCC TCACACCTCC ACACTGACAG ATCTGAATGT TGGAATAGGT GATTCAGAAG GGTTGCATTA TGATGGAAAG AAATTGATTG TTATAGGGTA TGGCGGTGCT TTGGCCTCCC

Consensus ATGTCTCTGC CCTCCATGCC TCACACCTCC ACACTGACAG ATCTGAATGT TGGAATAGGT GATTCAGAAG GGTTGCATTA TGATGGAAAG AAATTGATTG TTATAGGGTA TGGCGGTGCT TTGGCCTCCC

3901 4030

Lr1_Victo_allele ACAATCTGGA TACAGTAGAA GATATGATTG TCGAAAGATG CGACGGTTTG TTCCCTGAAG ATTTGGATGG CAGTTTTGTC TTCCGTTCAG TTAAGAATCT CACATTACAT GTATCTCGTC TTACCAGCAG

Lr1_Glenlea_allele ACAATCTGGA TACAGTAGAA GATATGATTG TCGAAAGATG CGACGGTTTG TTCCCTGAAG ATTTGGATGG CAGTTTTGTC TTCCGTTCAG TTAAGAATCT CACATTACAT GTATCTCGTC TTACCAGCAG

Consensus ACAATCTGGA TACAGTAGAA GATATGATTG TCGAAAGATG CGACGGTTTG TTCCCTGAAG ATTTGGATGG CAGTTTTGTC TTCCGTTCAG TTAAGAATCT CACATTACAT GTATCTCGTC TTACCAGCAG

4031 4160

Lr1_Victo_allele CAAATCATCA TCGTGAAAAG TGTTAAACTG TTTCCCAGCT CTTTCTGTGT TGGTGATAGT TGGCTATGAG GAATGTGTAA TGCAGTTCCC ATCATCCAGC TCACTGCAGA AACTTACCTT CTCAGGGTGT

Lr1_Glenlea_allele CAAATCATCA TCGTCAAAAG TGTTAAACTG TTTCCCAGCT CTTTCTGTGT TGGTGATAGT TGGCTATGAG GAATGTGTAA TGCAGTTCCC ATCATCCAGC TCACTGCAGA AACTTACCTT CTCAGGCTGC

Consensus CAAATCATCA TCGTcAAAAG TGTTAAACTG TTTCCCAGCT CTTTCTGTGT TGGTGATAGT TGGCTATGAG GAATGTGTAA TGCAGTTCCC ATCATCCAGC TCACTGCAGA AACTTACCTT CTCAGGcTGc

4161 4290

Lr1_Victo_allele AAGGGCCTAG TTCTTGTGCC TGTGGAGAA- -----TGGAG GAGGAATTCA GGAGGACAAG TCATTGCTCC AATCATTAAC CATAGTCAGC TGTGGCGAAT TGTTCTGTCG GTGGCC---- --AATGAGAG

Lr1_Glenlea_allele CGGGGCCTGG TTCTTGTGCC TGAGGAGAAG GAGAATGGAG GAGGAATTCA GGAGGACAAC TCATTGCTCC AATCATTAAC AATAGTGGGA TGTGGCAAAC TCTTCTCTCG TTGGCCCATG GGAATGGGAG

Consensus aaGGGCCTaG TTCTTGTGCC TGaGGAGAA. .....TGGAG GAGGAATTCA GGAGGACAAc TCATTGCTCC AATCATTAAC aATAGTcaGa TGTGGCaAAc TcTTCTcTCG gTGGCC.... ..AATGaGAG

4291 4420

Lr1_Victo_allele AATCAGAGAC CATTTGCCCT TTCCCTGCTT CCCTGAGGGA ACTTGATGTT TTCCAAGAGC CAAGCATGAA GTCAATGGCT CTGCTCTCAA ACCTCACGTC TCTCACCACT CTACAGCTAA ACTACTGCAG

Lr1_Glenlea_allele AATCAGAGAC CATTTGCCCT TTCCCTGCTT CCCTGAAGAA ACTTGATGTT TTCCAAGAGC CAAGCATGAA GTCAATGGCT CTGCTCTCAA ACCTCACGTC TCTCACCACT CTACAGCTAA ACTACTGCAG

Consensus AATCAGAGAC CATTTGCCCT TTCCCTGCTT CCCTGAaGaA ACTTGATGTT TTCCAAGAGC CAAGCATGAA GTCAATGGCT CTGCTCTCAA ACCTCACGTC TCTCACCACT CTACAGCTAA ACTACTGCAG

4421 4550

Lr1_Victo_allele TAATTTAACA GTGGATGGAT TCAATCCTCT CATCGCAGTC AACCTCATAG AGCTGCAAGT GCATAGGTGC AACACCTTAG CAGCAGATAT GCTCTCAGAG GCGGCCTCTC ACTCTCAGAG GGCCAAATTA

Lr1_Glenlea_allele TAATTTAACA GTGGATGGAT TCAATCCTCT CATCGCAGTC AACCTCATAG AGCTGCAAGT GCATAGGTGC AACACCTTAG CAGCAGATAT GCTCTCAGAG GCGGCCTCTC ACTCTCAGAG GGCCAAATTA

Consensus TAATTTAACA GTGGATGGAT TCAATCCTCT CATCGCAGTC AACCTCATAG AGCTGCAAGT GCATAGGTGC AACACCTTAG CAGCAGATAT GCTCTCAGAG GCGGCCTCTC ACTCTCAGAG GGCCAAATTA

4551 4680

Lr1_Victo_allele TTGCCTGCAG GTTACATCTC TAGATTGGAG GTACTCATCG TGGATAACAT CTGTGGATTG CTTGTTGCTC CTATTTGCAT CCTCCTCGCC CCGGCCCTCC ACACACTTGT ATTCTGGATT GATGAAACGA

Lr1_Glenlea_allele TTGCCTGCAG GTTACATCTC TAGATTGGAG AAACTCAATG TGGATAACAA CTGTGGATTG CTTGTTGCTC CTATTTGCAA CCTCCTCGCC CCGGCCCTCC ACACACTTGT ATTCTGGATT GATGAAACGA

Consensus TTGCCTGCAG GTTACATCTC TAGATTGGAG aaACTCAacG TGGATAACAa CTGTGGATTG CTTGTTGCTC CTATTTGCAa CCTCCTCGCC CCGGCCCTCC ACACACTTGT ATTCTGGATT GATGAAACGA

4681 4810

Lr1_Victo_allele TGGAAAGCTT GACGGAAGAG CAGGAGAAAG CGCTGCAGCT CCTCACCTCC CTCCAGAATC TAACATTTTT CAGATGCAGG GGTCTACAGT CCCTTCCTCA AGGGTTGCAT CGCCTTTCTT CTCTCAAGGA

Lr1_Glenlea_allele TGGAAAGCTT GACGGAAGAG CAAGAGAAAG CGCTGCAGCT CCTCACCTCC CTCCAGAATC TAACATTTTT CAGATGCAGG GGTCTACAGT CCCTTCCTCA AGGGTTGCAT CGCCTTTCTT CTCTCAAGGA

Consensus TGGAAAGCTT GACGGAAGAG CAaGAGAAAG CGCTGCAGCT CCTCACCTCC CTCCAGAATC TAACATTTTT CAGATGCAGG GGTCTACAGT CCCTTCCTCA AGGGTTGCAT CGCCTTTCTT CTCTCAAGGA

4811 4940

Lr1_Victo_allele GTTATGTGTC CGTGGGTGTC TAAAAATCCA ATCGTTGCCC AAGGAGGGCC TCCCGCTTTC GCTGAGAAGA CTAAAGATGA ATTGGCGCAG CGCTGAGATA AACGAGCAAA TTGAGAAAAT CAAAAGAAGC

Lr1_Glenlea_allele GTTATGTGTC CGTGGGTGTC TAAAAATCCA ATCGTTGCCC AAGGAGGGCC TCCCGCTTTC GCTGAGAAGA CTAAAGATGA ATTGGCGCAG CGCTGAGATA AACGAGCAAA TTGAGAAAAT CAAAAGAAGC

Consensus GTTATGTGTC CGTGGGTGTC TAAAAATCCA ATCGTTGCCC AAGGAGGGCC TCCCGCTTTC GCTGAGAAGA CTAAAGATGA ATTGGCGCAG CGCTGAGATA AACGAGCAAA TTGAGAAAAT CAAAAGAAGC

4941 5070

Lr1_Victo_allele AACCCAGATT TATCCGTCTC GTATTGCTAA CTACACCCAA GGTAACACTT GTCGCCTCCC TATTTTGATT GTTTCTCTAT TTCTGATGAA ACGAGGTTTA TTCGCCATCC ATATATATTT CTGCCTCATC

Lr1_Glenlea_allele AACCCAGATT TATCCGTCTC GTATTGCTAA CTACACCCAA GGTAACACTT GTCGCCTCCC TATTTTGATT GTTTCTCTAT TTCTGATGAA ACGAGGTTTA TTCGCCATCC ATATATATTT CTGCCTCATC

Consensus AACCCAGATT TATCCGTCTC GTATTGCTAA CTACACCCAA GGTAACACTT GTCGCCTCCC TATTTTGATT GTTTCTCTAT TTCTGATGAA ACGAGGTTTA TTCGCCATCC ATATATATTT CTGCCTCATC

5071 5200

Lr1_Victo_allele TTAACCACTG TTGTCAAATC TTACAGACTA GCAGGTTCCA CTGTTGCAAT CAATCTTTTG TGCACACAGG TTGTGAAACC TGCACATTAA TCAGCAGGTC AGGTGCGAGA TACAACCTAC CGACTCTTCA

Lr1_Glenlea_allele TTAACCACTG TTGTCAAATC TTACAGACTA GCAGGTTCCA CTGTTGCAAT CAATCTTTTG TGCACACAGG TTGTGAAACC TGCACATTAA TCAGCAGGTC AGGTGCGAGA TACAACCTAC CGACTCTTCA

Consensus TTAACCACTG TTGTCAAATC TTACAGACTA GCAGGTTCCA CTGTTGCAAT CAATCTTTTG TGCACACAGG TTGTGAAACC TGCACATTAA TCAGCAGGTC AGGTGCGAGA TACAACCTAC CGACTCTTCA

5201 5330

Lr1_Victo_allele TTCTGCAACG CAACACTAGC TGTGAACTCT TCATTCTGCT GCATGATCTG CCGGTTAGTA CCTCTCCACC TCCAGAACCA AATTCCTTTT TTTAGAAAAG GAGGATATGA CCCCCGGCCT CTGCATCTGG

Lr1_Glenlea_allele TTCTGCAACG CAACACTAGC TGTGAACTCT TCATTCTGCT GCATGATCTG CCGGTTAGTA CCTCTCCACC TCCAGAACCA AATTCCTTTT TTTAGAAAAG GAGGATATGA CCCCCGGCCT CTGCATCTGG

Consensus TTCTGCAACG CAACACTAGC TGTGAACTCT TCATTCTGCT GCATGATCTG CCGGTTAGTA CCTCTCCACC TCCAGAACCA AATTCCTTTT TTTAGAAAAG GAGGATATGA CCCCCGGCCT CTGCATCTGG

5331 5460

Lr1_Victo_allele GCGATGCATA CGGCCACTCC AGAACCAAAT TCCTGATCCT TCATTTATGA CTGCATTCAT TTCATTTAGG CCTCCTGAGT AGCTGCGTTA TTGTTATAGG AGTTGCCTAT TATTCCAGCT TGTTCTTCAT

Lr1_Glenlea_allele GCGATGCATA CGGCCACTCC AGAACCAAAT TCCTGATCCT TCATTTATGA CTGCATTCAT TTCATTTAGG CCTCCTGAGT AGCTGCGTTA TTGTTATAGG AGTTGCCTAT TATTCCAGCT TGTTCTTCAT

Consensus GCGATGCATA CGGCCACTCC AGAACCAAAT TCCTGATCCT TCATTTATGA CTGCATTCAT TTCATTTAGG CCTCCTGAGT AGCTGCGTTA TTGTTATAGG AGTTGCCTAT TATTCCAGCT TGTTCTTCAT

5461 5493

Lr1_Victo_allele TGTCTGACCC AAGAATAGTT GGTTACCCGA ATT

Lr1_Glenlea_allele TGTCTGACCC AAGAATAGTT GGTTACCCGA ATT

Consensus TGTCTGACCC AAGAATAGTT GGTTACCCGA ATT
