# Supplementary figures and images for: Characterization of the Resistance to Powdery Mildew and Leaf Rust Carried by the Bread Wheat Cultivar Victo
Source: Int J Mol Sci. 2021 Mar 18;22(6):3109. doi: 10.3390/ijms22063109 (PMC8003046; doi:10.3390/ijms22063109)

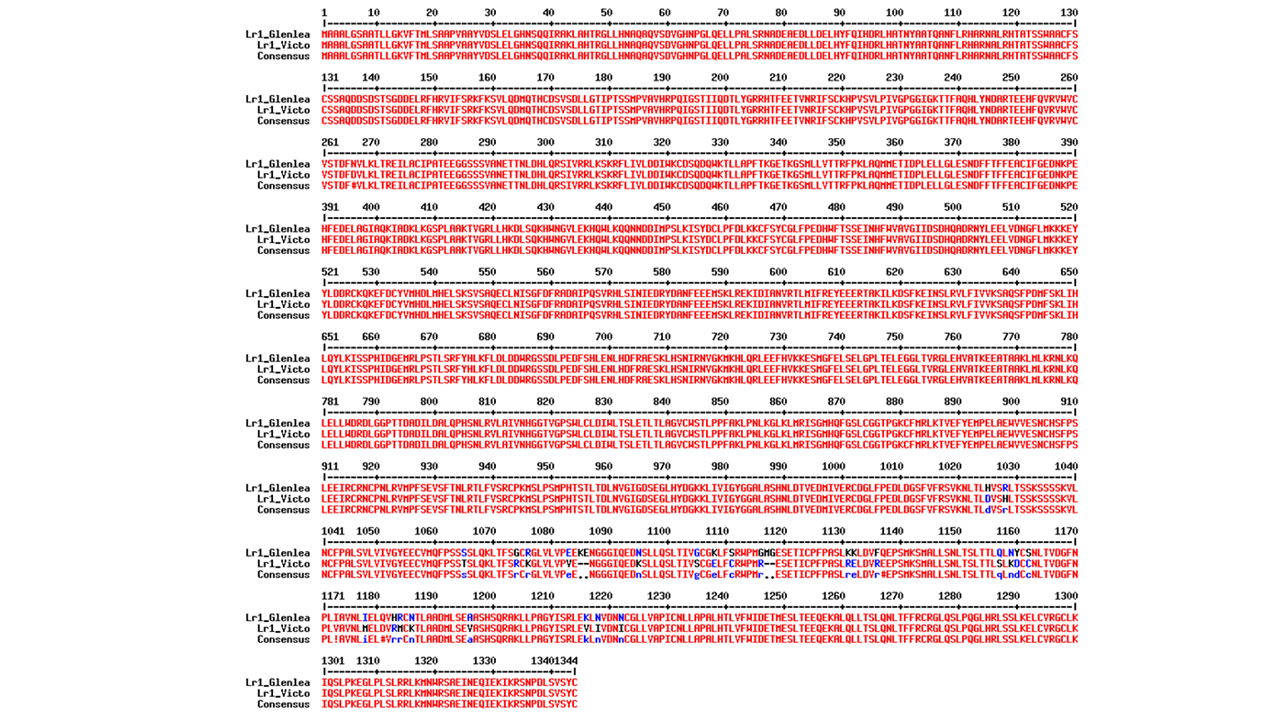

Supplement: Supplementary file 1 [file ijms-22-03109-s001.zip › Supplementary_Files/Figure S1.tif]
